# Patient decision support interventions for candidates considering elective surgeries: a systematic review and meta-analysis

**DOI:** 10.1097/JS9.0000000000000302

**Published:** 2023-04-10

**Authors:** Ling Jie Cheng, Nick Bansback, Meixia Liao, Vivien Xi Wu, Wenru Wang, Gabriel Ka Po Liu, Hwee Weng Dennis Hey, Nan Luo

**Affiliations:** a Saw Swee Hock School of Public Health, National University of Singapore, Singapore; b School of Population and Public Health, University of British Columbia, Vancouver, British Columbia, Canada; c Alice Lee Centre for Nursing Studies, Yong Loo Lin School of Medicine, National University of Singapore, Singapore; d Department of Orthopaedic Surgery, Yong Loo Lin School of Medicine, National University of Singapore, Singapore; e Department of Orthopaedic Surgery, National University Hospital, National University Health System, Singapore

**Keywords:** decisional conflict, elective surgery, meta-analysis, patient decision support interventions, systematic review, treatment choice

## Abstract

The increase in elective surgeries and varied postoperative patient outcomes has boosted the use of patient decision support interventions (PDSIs). However, evidence on the effectiveness of PDSIs are not updated. This systematic review aims to summarize the effects of PDSIs for surgical candidates considering elective surgeries and to identify their moderators with an emphasis on the type of targeted surgery.

**Design:**

Systematic review and meta-analysis.

**Methods:**

We searched eight electronic databases for randomized controlled trials evaluating PDSIs among elective surgical candidates. We documented the effects on invasive treatment choice, decision-making–related outcomes, patient-reported outcomes, and healthcare resource use. The Cochrane Risk of Bias Tool version 2 and Grading of Recommendations, Assessment, Development, and Evaluations were adopted to rate the risk of bias of individual trials and certainty of evidence, respectively. STATA 16 software was used to conduct the meta-analysis.

**Results:**

Fifty-eight trials comprising 14 981 adults from 11 countries were included. Overall, PDSIs had no effect on invasive treatment choice (risk ratio=0.97; 95% CI: 0.90, 1.04), consultation time (mean difference=0.04 min; 95% CI: −0.17, 0.24), or patient-reported outcomes, but had a beneficial effect on decisional conflict (Hedges’ *g*=−0.29; 95% CI: −0.41, −0.16), disease and treatment knowledge (Hedges’ *g*=0.32; 95% CI: 0.15, 0.49), decision-making preparedness (Hedges’ *g*=0.22; 95% CI: 0.09, 0.34), and decision quality (risk ratio=1.98; 95% CI: 1.15, 3.39). Treatment choice varied with surgery type and self-guided PDSIs had a greater effect on disease and treatment knowledge enhancement than clinician-delivered PDSIs.

**Conclusions:**

This review has demonstrated that PDSIs targeting individuals considering elective surgeries had benefited their decision-making by reducing decisional conflict and increasing disease and treatment knowledge, decision-making preparedness, and decision quality. These findings may be used to guide the development and evaluation of new PDSIs for elective surgical care.

## Introduction

Highlights

- Patient decision support interventions (PDSIs) had no effect on invasive treatment choice, consultation time, or patient-reported outcomes (PROs).
- PDSIs had a beneficial effect on decisional conflict, disease and treatment knowledge, decision-making preparedness, and decision quality.
- Treatment choice varied with surgery type.
- Self-guided PDSIs had a greater effect on disease and treatment knowledge enhancement than clinician-delivered PDSIs.

The global increase in elective surgeries^1^,^2^ and varied postoperative patient outcomes are growing public health concerns^3^. Undesirable patient outcomes such as postoperative complications^3^ and decreased health-related quality of life (HRQoL)^4^ are common. Therefore, surgical decisions should be ‘preference-sensitive’^5^, namely, guided by patient preferences when several options are available or patient outcomes are uncertain.

PDSIs have been used to enhance the preference-sensitive nature of clinical decision-making. PDSIs present evidence-based information to patients about a health condition, treatment options, and the associated benefits and risks, and implicitly or explicitly clarify the value patients place on the treatment benefits and risks^5^. The primary goal of PDSIs is to enhance decision-making quality and facilitate patient engagement during consultations^6^. PDSIs may also assist patients by increasing their knowledge of the available options and outcomes, thereby equipping them with more realistic expectations^6^.

With the increasing availability of validated PDSIs for elective surgical candidates faced with a treatment decision^7^–^9^, it is essential to investigate the effects for various types of elective surgery. In general, PDSIs have been found to improve disease and treatment knowledge^5^,^8^,^9^, satisfaction with decision-making^9^, decision quality^8^, and reduce decision conflict^5^,^8^,^9^. However, these reviews included different types of PDSIs^8^,^9^, study designs^8^,^9^, and elective surgery is quite unique.

Knops *et al.* 7 concluded that PDSIs increased knowledge and decreased decision conflict, but had no effect on anxiety and postoperative HRQoL. However, the review is outdated^7^. Therefore, we updated this review with the aims of (1) summarizing the effects of PDSIs on invasive treatment choice, decision-making–related outcomes, PROs, and healthcare resource utilization outcomes for patients considering elective surgery, and (2) identifying the moderators of PDSIs effects, with an emphasis on the type of targeted surgery.

## Methods

### Protocol and registration

This systematic review and meta-analysis were conducted according to the Preferred Reporting Items for Systematic Review and Meta-Analysis (PRISMA) guidelines (Supplementary Table 1, Supplemental Digital Content 1, http://links.lww.com/JS9/A238)^10^. The protocol was registered in the International Prospective Register of Systematic Reviews database (PROSPERO Number: CRD42021273767).

### Eligibility criteria

The eligibility criteria is illustrated in Supplementary Table 2 (Supplemental Digital Content 1, http://links.lww.com/JS9/A238). We considered all types of randomized controlled trials (RCTs), including both published and unpublished trials. The trials had to enroll surgical candidates who were contemplating elective surgeries, defined as a surgical procedure that is scheduled in advance because it does not involve a medical emergency. The included trials must have assessed one or more PDSIs, defined as tools designed to inform patients and clinicians of their elective treatment options, surgical or both surgical and nonsurgical, among which none is the undisputable choice to all patients. They could take the form of computer software or physical tools. Comparators had to include an active control group, a standard care group, or a waitlist group. Outcomes had to include invasive treatment choices, decision-making–related outcomes, PROs, and outcomes related to healthcare resource use. The trials were limited to studies published in the English language, but not publication period. We excluded trials in which individuals had cognitive impairment or psychiatric disease.

### Information sources and search strategy

A three-step comprehensive search strategy was employed from inception to 30 August 2021, under the guidance of an experienced librarian team. First, we searched eight databases (search engines) (index and key terms provided in Supplementary Table 3, Supplemental Digital Content 1, http://links.lww.com/JS9/A238): Cumulative Index to Nursing and Allied Health Literature (EBSCO), Cochrane Central Register of Controlled Trials (Ovid), Excerpta Medica Database (Elsevier), ProQuest Dissertations and Theses (ProQuest), PsycINFO (Ovid), MEDLINE (PubMed), Scopus (Elsevier), and Web of Science (Clarivate). Second, we examined various clinical trial registries (Supplementary Table 4, Supplemental Digital Content 1, http://links.lww.com/JS9/A238) for relevant ongoing and unpublished trials. Third, we conducted a manual search on the reference lists of the related primary studies or systematic reviews. In addition, we searched specialized journals and grey literature databases for potential trials. The EndNote X20 software was used to manage the references and excluded duplicates^11^.

### Study selection and data extraction

**Figure 1:**
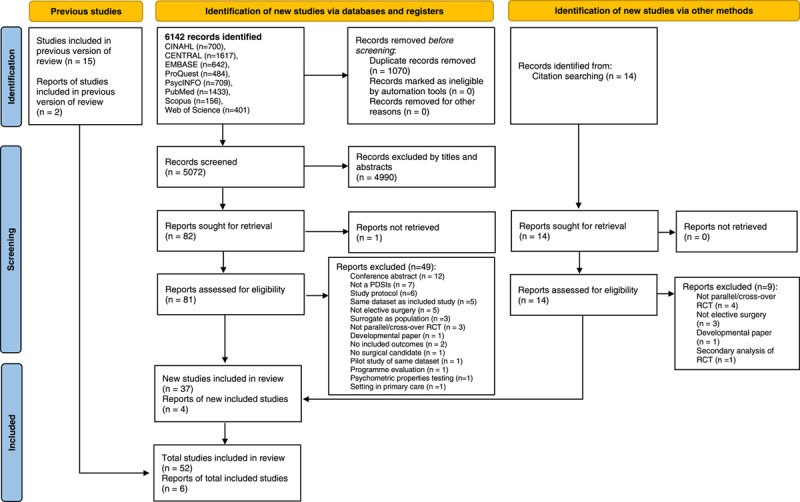
Preferred Reporting Items for Systematic Review and Meta-Analysis (PRISMA) 2020 flow diagram for updated systematic reviews. PDSI, patient decision support intervention; RCT, randomized controlled trial.

Study selection was graphically illustrated using the PRISMA 2020 flow diagram (Fig. 1). Two reviewers (L.J.C. and M.X.L.) independently screened the titles and abstracts to identify their relevance. When multiple reports of the same study were identified, studies were collated. The potential full texts were selected based on the eligibility criteria, and the reasons for inclusion/exclusion were recorded (Supplementary Table 5, Supplemental Digital Content 1, http://links.lww.com/JS9/A238). Any disagreements were resolved by a third reviewer (N.L.).

A standardized data extraction form was developed using the Cochrane Handbook for Systematic Reviews of Interventions^12^. Three essential components were included: trial characteristics, PDSIs characteristics, and key outcomes related to decision-making, PROs, and healthcare resource use. Based on a reporting guide^13^,^14^, the following characteristics of PDSIs were extracted: aim, element, platform, co-intervention, duration, media format, use of PROs data, artificial intelligence embodiment, value consideration, theoretical framework, communication, facilitator, survey administration methods at various time points, and assessment intervals.

Invasive treatment choice included the actual choice of invasive treatment implemented; if not specified, the participant’s preferred option was used as a surrogate measure^7^. Decision-making–related outcomes (measures) included decisional conflict (different versions of Decisional Conflict Scale^15^), satisfaction with decision-making (effective decision subscale of Decisional Conflict Scale and self-developed patient satisfaction surveys), disease and treatment knowledge (self-developed questionnaires), decisional regret (Decision Regret Scale^16^), Preparedness For Decision-Making (Rochester Participatory Decision-Making Scale^17^ and Preparedness For Decision-Making Scale^18^), decision quality (Decision Quality Instrument^19^), Shared Decision-Making (CollaboRATE^20^ and Nine-Item Shared Decision-Making Questionnaire^21^), Decision Self-Efficacy (Self-Efficacy For Managing Chronic Diseases Six-Item^22^ and Decision Self-Efficacy Scale^23^), and outcome expectations. PROs included HRQoL (general or condition-specific), physical health, mental health, depression, anxiety, and perceived stress. Outcomes related to healthcare resource use included consultation time. Detailed definitions for all extracted outcomes and the measures used are listed in Supplementary Table 6 (Supplemental Digital Content 1, http://links.lww.com/JS9/A238).

The data extraction form was piloted on ten trials to ensure that the items were accurate and appropriate. Two independent reviewers (L.J.C. and M.X.L.) retrieved data from 58 trials following item confirmation. If trials reported data on the median, range, interquartile range, and SE, data conversion was utilized to compute the mean and SD using recommended formulae^12^,^24^,^25^. When data was questionable, insufficient, or missing, trial authors were contacted via email and asked to provide additional unpublished details or results.

### Quality of patient decision support interventions design

International Patient Decision Aid Standards instrument short-form (IPDASi-SF) was used to assess the quality of PDSIs^26^. The IPDASi-SF contains 16 items addressing seven dimensions related to the information about the options, probabilities, values, development, disclosure of funding, decision aid evaluation, and evidence used^26^. The IPDASi-SF score ranged from 0 to 16, with a higher score indicating better PDSIs quality.

### Risk of bias in individual studies

The Cochrane risk of bias (RoB 2.0)^27^ tool was used to assess the risk of bias in the selected randomised trials on five domains of bias: bias arising from the randomization process, bias due to deviations from the intended intervention, bias due to missing outcome data, bias in the measurement of the outcome, and bias in the selection of the reported result. Two reviewers (L.J.C. and M.X.L.) independently responded to each of the signaling questions with (1) yes, (2) probably yes, (3) probably no, (4) no, or (5) no information. The RoB 2.0 algorithmic tool rates the risk of bias as (1) low risk of bias, (2) some concerns, or (3) high risk of bias for each domain.

### Statistical analysis

The *Meta* command procedures in Stata 16 software were used to perform a meta-analysis and subgroup analysis^28^,^29^. *Z*-statistics with a significance level of *P*-value less than 0.05 was used to assess the overall effect^30^. A minimum of two studies was needed to perform a meta-analysis^31^. A weighted risk ratio (RR) (dichotomous data), standardized effect size, or mean difference (continuous data) was calculated for each outcome measure^32^. As most of the trials used a small sample size, the pooled effect sizes of outcomes (continuous data) were assessed using Hedges’ *g* 33. The effect magnitude was classified as small (≥0.2), medium (≥0.5), large (≥0.8), or very large (≥1.2)^34^. We used the DerSimonian and Laird procedure to estimate the variability between studies for random-effects meta-analysis^35^.

Statistical heterogeneity was assessed using *I* ^2^ statistics and Cochran’s *Q* test, with a *P*-value less than 0.10 indicating evidence of heterogeneity^36^. The degree of heterogeneity using overlapping intervals for *I* ^2^ was set as 0–40% (might not be important), 30–60% (moderate), 50–90% (substantial), and 75–100% (considerable)^37^. The source of heterogeneity was investigated using subgroup analysis^37^. For each outcome measure, whenever 10 or more trials were available, separate subgroup analysis was performed to evaluate five moderators coded into categorical variables including: types of elective surgery, patient-reported outcome measures–based PDSIs (yes/no), mode of delivery (self-guided/clinician-administered), value consideration (yes/no), and use of theoretical framework (yes/no).

### Reporting bias assessment

We used Begg’s test^38^, Egger’s regression test^39^, the asymmetry of the funnel plot^40^, and the trim-and-fill approach^40^ to examine publication bias in meta-analyses with 10 or more trials^41^. Egger regression and Begg’s test were performed using Stata 16 software^28^, with a *P*-value more than 0.05 indicating no small-study effects existed. We identified the possibility of heterogeneity in effect sizes across studies, limiting the conclusions drawn from Egger’s tests and the funnel plot^42^,^43^. We also applied the Copas selection model, using the *metafor* package in R software^44^, to account for selection bias according to funnel plot asymmetry^45^,^46^.

### Certainty assessment

GRADEpro 3.6 software^47^ was used to assess the certainty of evidence using the Grading of Recommendations Assessment, Development, and Evaluation (GRADE) approach^48^. The GRADE assessment focuses on five factors: methodological limitations, imprecision, inconsistency, indirectness, and publication bias^49^. When issues were detected in the five factors, the evidence was downgraded. The certainty of the evidence was graded as high, moderate, low, and very low.

## Results

### Study selection

Figure 1 illustrates the study selection results. We identified 6142 records and eliminated 1070 duplicates. In addition, citation searching yielded 14 records and 15 studies, as well as two reports, from a previous review^7^. After two reviewers screened independently, 4990 records were excluded based on their titles and abstracts. Eighty-two full-text articles were selected, and 50 were excluded for several reasons (Supplementary Table 5, Supplemental Digital Content 1, http://links.lww.com/JS9/A238). Finally, 58 trials^50^–^107^ were included in the review, with 52 unique trials and six reports linked to the unique trials (Supplementary Table 7, Supplemental Digital Content 1, http://links.lww.com/JS9/A238). The inter-rater agreement of the two reviewers was consistent (κ=0.87, *P*<0.001).

### Trial characteristics

The characteristics of trials, published between 1995 and 2021, are summarized in Table 1. The sample size ranged from 16 to 1485 participants, with a mean age between 23.6 and 72.0 years. The attrition rate varied between 0% and 44.8%. Of these, 52 trials (89.7%) compared PDSIs with usual care, with the remaining six trials comparing different PDSIs. Thirty-two trials (55.2%) were conducted in the USA, with seven in Canada (12.1%), six in The Netherlands (10.3%), three in Australia (5.2%), three in the UK (5.2%), two in Finland (3.4%), and only one in China, Germany, Hong Kong, Spain, and Turkey. Fifty-two trials (89.7%) utilized a two-arm RCT design, two trials used a three-arm RCT and a cluster RCT design, and one trial used a stepped wedge trial design. Most of the trials studied PDSIs developed for patients with neoplasms (number of trials, *k*=25, 43.1%), diseases of the musculoskeletal system (*k*=18, 31.0%), or diseases of the genitourinary system (*k*=8, 13.8%). Most of the PDSIs evaluated involved decisions about elective gynecology and obstetrics surgery (*k*=21, 36.2%) or orthopedic surgery (*k*=14, 24.1%).

**Table 1:** Characteristics of selected randomized controlled trials

References/country	Design	Nature of population	Types of elective surgery	Age (mean±SD)	Sample size	Sex (female/male)	Interventions/mode of delivery	Comparisons (comparator)	Outcomes (measures)	Attrition rates (%)	ITT/MDM	Protocol/registration/grant
Allen *et al.* 50/USA	2-arm RCT	Hip and knee osteoarthritis	Total hip or knee joint replacement	61.8±11.7	T: 155 I: 80 C: 75	94/61	Video-based/self-guided	Active control (computer-based)	Decision conflict (DCS); Knowledge (K-DQI); Preparedness for decision-making; Outcome expectations	5.8	Y/N	N/N/N
Allen *et al.* 51/USA	2-arm stepped-wedge RCT	Heart disease	Destination therapy LVAD placement	63.4±9.9	T: 248 I: 113 C: 135	39/209	Video-based/clinician	Usual care	Invasive treatment choice; Decisional conflict (DCS); Decisional regret (DRS); General HRQoL (EQ VAS); Depression (PHQ-2); Perceived stress (PSS)	33.5	Y/Y	Y/Y/Y
Arterburn *et al.* 52/USA	2-arm RCT	Obesity-related chronic health condition	Bariatric surgery	50.5±9.9	T: 152 I: 75 C: 77	111/41	Video-based/self-guided	Usual care (booklet control)	Invasive treatment choice; Decisional conflict (DCS); Knowledge; Decision self-efficacy (DSES)	4.6	Y/N	N/N/Y
Auvinen *et al.* 53/Finland	2-arm RCT	Prostate cancer	Radical prostatectomy/orchidectomy	59.9±26.6	T: 210 I: 104 C: 106	0/210	Paper-based/clinician	Usual care (standardized treatment protocol)	Invasive treatment choice	3.3	Y/Y	N N/Y
Berry *et al.* 54/USA	2-arm RCT	Prostate cancer	Prostatectomy	57.4±13.5	T: 392 I: 198 C: 194	0/392	Computer-based/self-guided	Usual care (links+materials provided in clinic)	Decisional conflict (DCS)	29.6	N/Y	N/Y/Y
Bozic *et al.* 55/USA	2-arm RCT	Osteoarthritis	Hip or knee replacement	60±NR	T: 198 I: 95 C: 103	NR/NR	Video-based/self-guided	Usual care	Invasive treatment choice	37.9	N/N	N/Y/Y
Coylewright *et al.* 56/USA	2-arm RCT	Heart disease	Percutaneous coronary intervention	68.2±10.5	T: 124 I: 65 C: 59	33/91	Paper-based/clinician	Usual care	Decisional conflict (DCS)	7.3	Y/Y	N/Y/Y
De Achaval *et al.* 57/USA	3-arm RCT	Knee osteoarthritis	Total knee arthroplasty	62.8±9.0	T: 208 I1: 70 I2: 69 C: 69	141/67	Video-based/self-guided	Usual care (booklet control)	Decisional conflict (DCS); Satisfaction with decision-making (DCS)	1.4	N/N	N/N/Y
Deyo *et al.* 58/USA	2-arm RCT	Lumbar spinal stenosis and herniated disk, or nonspecific back pain	Lumbar spine surgery	52.4±16.5	T: 393 I: 190 C: 203	188/205	Video-based/self-guided	Usual care (booklet control)	Invasive treatment choice	12.5	Y/N	N/N/Y
Eden *et al.* 59/USA	2-arm RCT	Pregnant women	Cesarean section	31.1±NR	T: 131 I: 66 C: 65	131/0	Computer-based/self-guided	Usual care (brochure)	Invasive treatment choice; Decisional conflict (DCS)	0	Y/Y	N/Y/Y
Goel *et al.* 60/Canada	2-arm cluster RCT	Breast cancer	Lumpectomy and mastectomy	57.5±12.2	T: 136 I: 86 C: 50	136/0	Audio-based/self-guided	Usual care (pamphlet)	Decisional conflict (DCS); Satisfaction with decision-making (DCS); Knowledge (BCIT-R)	21.3	N/N	N/N/N
Gökce *et al.* 61/Turkey	2-arm RCT	Renal stone	SWL and RIRS	46.3±5.8	T: 119 I: 60 C: 59	52/67	Paper-based/self-guided	Usual care (standard information)	Invasive treatment choice; Decisional conflict (DCS)	3.4	N/N	N/N/N
Hawley *et al.* 62/USA	2-arm RCT	Breast cancer	Mastectomy	56.8±10.8	T: 537 I: 267 C: 270	537/0	Computer-based/self-guided	Active control (static version of icandecide)	Preparedness for decision-making; Decision quality (SDQ)	7.6	Y/Y	Y/Y/Y
Heller *et al.* 63/USA	2-arm RCT	Breast cancer	Breast reconstruction	47.0±9.4	T: 133 I: 66 C: 67	133/0	Computer-based/self-guided	Usual care (standard patient education)	Satisfaction with decision-making	0	N/N	N/N/N
Hutyra *et al.* 64/USA	2-arm RCT	Anterior shoulder dislocations	Operative treatment	23.6±5.3	T: 199 I: 100 C: 99	45/154	Computer-based/self-guided	Active control (text-based)	Invasive treatment choice; Decisional conflict (DCS)	0.5	N/N	N/N/Y
Ibrahim *et al.* 65/USA	2-arm RCT	Knee osteoarthritis	Total knee replacement	59.1±7.2	T: 336 I: 168 C: 168	235/101	Video-based/self-guided	Usual care (educational booklet)	Invasive treatment choice	9.5	Y/N	Y/Y/Y
Jayakumar *et al.* 66/USA	2-arm RCT	Knee osteoarthritis	Total knee arthroplasty	62.6±8.4	T: 145 I: 76 C: 69	83/62	Computer-based/clinician	Usual care (educational material)	Invasive treatment choice; Satisfaction with decision-making; Decision quality (K-DQI); Shared decision-making (CollaboRATE); Condition-specific HRQoL (KOOS JR)	11	N/N	Y/Y/Y
Jibaja-Weiss *et al.* 67/USA	2-arm RCT	Breast cancer	Mastectomy	51.0±10.9	T: 100 I: 51 C: 49	100/0	Computer-based/clinician	Usual care	Invasive treatment choice; Satisfaction with decision-making (SWDMP)	24	N/N	N/N/Y
Kearing *et al.* 68/USA	2-arm RCT	Lumbar spinal stenosis	Operative treatment	66.6±9.7	T: 199 I: 98 C: 101	81/118	Video-based/self-guided	Active control (video-based)	Invasive treatment choice	15.6	N/N	N/N/Y
Kennedy *et al.* 69/UK	3-arm RCT	Menorrhagia	Hysterectomy	40.3±7.0	T: 894 I1: 296 *I* ^2^: 300 C: 298	894/0	Video-based/self-guided	Usual care	Invasive treatment choice	30.1	N/Y	N/N/Y
Kleiss *et al.* 70/USA	2-arm RCT	Upper-extremity conditions	Operative treatment	55±14	T: 147 I: 76 C: 71	98/49	Computer-based/clinician	Usual care (no intervention)	Satisfaction with decision-making; Decisional regret (DRS); Physical Health (PROMIS PF)	31.3	N/N	N/Y/N
Korteland *et al.* 71/The Netherlands	2-arm RCT	Heart disease	Prosthetic heart valve selection	61.0±16.3	T: 138 I: 67 C: 71	34/104	Computer-based/self-guided	Usual care (standard preoperative care)	Decisional conflict (DCS); General Health (SF-36); Physical Health (SF-36); Mental Health (SF-36)	11	Y/Y	N/Y/Y
Kostick *et al.* 72/USA	2-arm RCT	Heart disease	LVAD support	59.8±12.1	T: 98 I: 47 C: 51	23/75	Paper-based/clinician	Usual care (standard care)	Invasive treatment choice; Decisional conflict (DCS); Satisfaction with decision-making; Decisional regret (ORS); Preparedness for decision-making; Shared decision-making (CollaboRATE); General Health	44.8	Y/Y	N/Y/Y
Kuppermann *et al.* 73/USA	2-arm RCT	Pregnant women	Repeat cesarean section	34.1±4.5	T: 1485 I: 742 C: 743	1485/0	Computer-based/clinician	Usual care	Invasive treatment choice; Decisional conflict (DCS); Satisfaction with decision-making (SWD); Knowledge; Shared decision-making (SDM-Q-9); Decision self-efficacy (DSES)	1.0	N/Y	Y/N/Y
Lam *et al.* 74/Hong Kong	2-arm RCT	Breast cancer	Mastectomy	55.7±10.5	T: 276 I: 138 C: 138	276/0	Paper-based/self-guided	Usual care (standard information booklet)	Invasive treatment choice; Decisional conflict (DCS); Knowledge; Decisional regret (DRS); Outcome expectations; Mental health (CHQ); Anxiety (HADS); Depression (HADS)	18.5	Y/N	N/N/Y
Lamers *et al.* 75/The Netherlands	2-arm RCT	Prostate cancer	Radical prostatectomy	65.3±5.9	T: 382 I: 273 C: 109	0/382	Computer-based/self-guided	Usual care	Invasive treatment choice	12.0	N/N	Y/Y/Y
Luan *et al.* 76/USA	2-arm RCT	Breast cancer	Breast reconstruction for mastectomy	49.2±3.4	T: 16 I: 8 C: 8	16/0	Paper-based/self-guided	Usual care (standard preconsultation material)	Invasive treatment choice; Decisional conflict (DCS); Decisional regret (DRS); Condition-specific HRQoL (BREAST-Q)	0	N/N	N/N/N
Manne *et al.* 78/USA	2-arm RCT	Breast cancer	Breast reconstruction for mastectomy	50.2±10.6	T: 55 I: 31 C: 24	55/0	Computer-based/self-guided	Usual care (pamphlet)	Invasive treatment choice; Decisional conflict (DCS); Satisfaction with decision-making; Knowledge; Anxiety (STAI)	21.8	Y/Y	N/N/Y
Manne *et al.* 77/USA	2-arm RCT	Breast cancer	Contralateral prophylactic mastectomy	46.5±8.4	T: 93 I: 46 C: 47	93/0	Computer-based/self-guided	Usual care	Invasive treatment choice; Satisfaction with decision-making; Knowledge; Preparedness for decision-making (OPDMS)	10.8	Y/Y	N/N/Y
Metcalfe *et al.* 79/Canada	2-arm RCT	BRCA1/2 mutation	Prophylactic mastectomy/oophorectomy	39.1±8.8	T: 150 I: 76 C: 74	150/0	Paper-based/self-guided	Usual care (standard genetic counseling)	Decisional conflict (DCS); Knowledge; Perceived stress (IES)	7.0	Y/Y	N/N/N
Montgomery *et al.* 80/UK	3-arm RCT	Pregnant women	Cesarean section	32.4±4.7	T: 742 I1: 245 *I* ^2^: 250 C: 247	742/0	Computer-based/self-guided	Usual care	Invasive treatment choice; Decisional conflict (DCS); Satisfaction with decision-making (SDS); Knowledge; Anxiety (STAI)	3.6	Y/N	Y/Y/Y
Parkinson *et al.* 81/Australia	2-arm RCT	Women with breast cancer or ductal carcinoma	Breast reconstruction following mastectomy	51.9±9.5	T: 222 I: 116 C: 106	222/0	Computer-based/self-guided	Usual care (standard online information)	General HRQoL (QALYs)	26.9	Y/Y	N/Y/Y
Phelan *et al.* 82/USA	2-arm RCT	Lumbar spinal stenosis	Lumbar spine surgery	49.5±18.8	T: 100 I: 47 C: 53	44/56	Computer-based/self-guided	Usual care (booklet control)	Invasive treatment choice	10	Y/N	N/N/Y
Politi *et al.* 83/USA	2-arm RCT	Breast cancer	Post-mastectomy breast reconstruction	50.7±10.8	T: 120 I: 60 C: 60	120/0	Computer-based/self-guided	Enhanced usual care	Invasive treatment choice; Decisional conflict (SURE); Decision quality (DQI); Condition-specific HRQoL (BREAST-Q)	0	Y/Y	N/Y/Y
Rivero-Santana *et al.* 84/Spain	2-arm RCT	Knee osteoarthritis	Total knee replacement	66.8±8.4	T: 193 I: 97 C: 96	139/54	Computer-based/self-guided	Usual care	Invasive treatment choice; Decisional conflict (DCS); Satisfaction with decision-making; Knowledge (K-DQI); Decisional regret (DRS)	3.6	N/N	N/Y/Y
Schwartz *et al.* 85/USA	2-arm RCT	BRCA1/BRCA2 mutation carriers	Mastectomy	43.9±10.9	T: 214 I: 100 C: 114	214/0	Computer-based/self-guided	Usual care (booklet control)	Invasive treatment choice	12.1	Y/N	N/N/Y
Sherman *et al.* 86/Australia	2-arm RCT	Women with breast cancer or ductal carcinoma	Breast reconstruction following mastectomy	51.9±9.5	T: 222 I: 116 C: 106	222/0	Computer-based/SELF-guided	Usual care (standard online information)	Decisional conflict (DCS); Satisfaction with decision-making; Decisional regret (DRS)	26.9	Y/N	N/Y/Y
Shorten *et al.* 87/Australia	2-arm RCT	Pregnant Women	Repeat cesarean section	31.8±NR	T: 227 I: 115 C: 112	227/0	Paper-based/self-guided	Usual care (routine pregnancy care)	Invasive treatment choice	25.6	N/N	N/N/Y
Shue *et al.* 88/USA	2-arm RCT	Hip and knee osteoarthritis	Total hip or knee joint replacement	61±11	T: 147 I: 73 C: 74	78/69	Video-based/self-guided	Active control (booklet-based)	Invasive treatment choice; Knowledge	10.2	N/N	N/N/Y
Stacey *et al.* 89/Canada	2-arm RCT	Osteoarthritis	Total joint arthroplasty	66.5±9.8	T: 334 I: 167 C: 167	192/142	Video-based/self-guided	Usual care (standard patient education)	Invasive treatment choice; Knowledge (K-DQI); Decision quality (K-DQI)	37	N/N	N/Y/Y
Stiggelbout *et al.* 90/The Netherlands	2-arm RCT	Asymptomatic abdominal aneurysm	Elective aneurysm repair	72.0±8.0	T: 100 I: 49 C: 51	7/93	Paper-based/clinician	Usual care (general brochure)	Invasive treatment choice	11.5	N/N	N/N/Y
Street *et al.* 91/USA	2-arm RCT	Breast cancer	Mastectomy	58.1±12.7	T: 60 I: 30 C: 30	60/0	Computer-based/self-guided	Usual care (Brochure)	Invasive treatment choice	0	N/N	N/N/Y
Trenaman *et al.* 93/Canada	2-arm RCT	Osteoarthritis	Total joint replacement	66.5±9.5	T: 334 I: 167 C: 167	192/142	Video-based/self-guided	Usual care (standard patient education)	General HRQoL (QALYs)	37	N/Y	N/Y/Y
Trenaman *et al.* 92/Canada	2-arm RCT	Osteoarthritis	Total joint replacement	66.6±9.8	T: 324 I: 161 C: 163	185/139	Video-based/self-guided	Usual care (standard patient education)	Invasive treatment choice	37	N/N	N/Y/Y
Tucholka *et al.* 94/USA	2-arm RCT	Breast cancer	Mastectomy	56.3±14.7	T: 227 I: 116 C: 111	227/0	Computer-based/self-guided	Usual care (standard website)	Knowledge (BCSDQI)	7.0	Y/N	N/Y/Y
van Roosmalen *et al.* 95/The Netherlands	2-arm RCT	Deleterious BRCA1/2 mutation	Prophylactic mastectomy/oophorectomy	43.6±10.8	T: 368 I: 184 C: 184	368/0	Video-based/self-guided	Usual care	Invasive treatment choice; Knowledge; General HRQoL; Anxiety (STAI); Depression (CES-D)	3.3 (only those withdrew)	Y/Y	N/N/Y
van Roosmalen *et al.* 96/The Netherlands	2-arm RCT	Deleterious BRCA1/2 mutation	Prophylactic mastectomy/oophorectomy	39.5±10.0	T: 88 I: 44 C: 44	88/0	Computer-based/clinician	Usual care	Invasive treatment choice; General HRQoL; Anxiety (STAI); Depression (CES-D); Perceived stress (IES)	1.1	Y/Y	N/N/Y
van Tol-Geerdink *et al.* 97/The Netherlands	2-arm RCT	Localized prostate cancer	Prostatectomy	64±5.0	T: 240 I: 163 C: 77	0/240	Paper-based/clinician	Usual care	Invasive treatment choice	0	Y/N	N/Y/Y
Vandemheen *et al.* 98/Canada, Australia	2-arm RCT	Cystic fibrosis	Lung transplantation	30.4±8.9	T: 149 I: 70 C: 79	68/81	Computer-based/self-guided	Usual care	Invasive treatment choice; Decisional conflict (DCS); Satisfaction with decision-making; Knowledge; Preparedness for decision-making; Outcome expectations	16.1	Y/N	N/Y/Y
Varelas *et al.* 99/USA	2-arm RCT	Breast cancer	Breast reconstruction following mastectomy	49.6±11.2	T: 47 I: 25 C: 22	26/0	Computer-based/self-guided	Usual care (consultation)	Decisional conflict (DCS); Condition-specific HRQoL (BREAST-Q); Anxiety (STAI)	44.7	N/N	N/N/N
Vina *et al.* 100/USA	2-arm RCT	Osteoarthritis	Knee replacement	61.6±8.0	T: 493 I: 240 C: 253	251/242	Video-based/self-guided	Usual care (booklet control)	Invasive treatment choice	0.6	N/Y	N/Y/Y
Vodermaier *et al.* 101/Germany	2-arm RCT	Breast cancer	Mastectomy	55.2±11.0	T: 111 I: 55 C: 56	111/0	Paper-based/clinician	Usual care (standard care)	Invasive treatment choice; Decisional conflict (DCS)	7.9	N/N	N/N/Y
Vuorma *et al.* 102/Finland	2-arm RCT	Menorrhagia	Hysterectomy	44.4±4.18	T: 363 I: 184 C: 179	363/0	Paper-based/self-guided	Usual care	Invasive treatment choice	13.2	Y/Y	N/N/Y
Whelan *et al.* 103/Canada	2-arm cluster RCT	Breast cancer	Mastecty/lumpectomy plus radiation	Median: 58.35	T: 201 I: 94 C: 107	201/0	Paper-based/clinician	Usual care	Invasive treatment choice; Decisional conflict (DCS); Satisfaction with decision-making (DCS); Anxiety (STAI); Depression (CES-D)	17.9	Y/N	N/N/Y
Wilkens *et al.* 104/USA	2-arm RCT	Osteoarthritis	Operative treatment	63.5±1.4	T: 90 I: 45 C: 45	65/25	Computer-based/self-guided	Usual care	Invasive treatment choice; Decisional conflict (DCS); Satisfaction with decision-making; Decisional regret (DRS); Condition-specific HRQoL (QuickDASH); Depression (PHQ-2)	7.8	Y/Y	N/N/N
Wilkins *et al.* 105/USA	2-arm RCT	Breast cancer	Mastectomy	54.9±9.80	T: 101 I: 52 C: 49	101/0	Video-based/self-guided	Active control (written educational materials)	Invasive treatment choice; Satisfaction with decision-making; Knowledge; Decision self-efficacy (SECPMD); Anxiety (STAI)	NR	N/N	N/N/Y
Wong *et al.* 106/UK	2-arm RCT	Pregnant women	Surgical termination of pregnancy	25±NR	T: 328 I: 163 C: 165	328/0	Paper-based/self-guided	Usual care (placebo leaflet)	Invasive treatment choice; Decisional conflict (DCS); Knowledge; Anxiety (STAI)	14.0	Y/N	N/N/N
Ye *et al.* 107/China	2-arm RCT	Cataract	Cataract surgery	64.3±0.3	T: 773 I: 386 C: 387	556/217	Paper-based/self-guided	Usual care (booklet control)	Invasive treatment choice; Decisional conflict (DCS); Decision quality	3.4	Y/Y	Y/Y/Y

BCIT-R, Breast Cancer Information Test – Revised; BCSDQI, Breast Cancer Surgery Decision Quality Instrument; CES-D, Center for Epidemiologic Studies Depression Scale; CHQ, Chinese Health Questionnaire; DCS, Decision Conflict Scale; DRS, Decision Regret Scale; DSES, Decision Self-Efficacy Scale; HADS, Hospital Anxiety and Depression Scale; HRQoL, health-related quality of life; IES, Impact of Event Scale; ITT, Intention-to-treat; K-DQI, Knee Osteoarthritis Decision Quality Instrument; KOOS JR, Knee Injury and Osteoarthritis Outcome Score Joint Replacement; LVAD, left ventricular assist device; MDM, missing data management; N, no; NR, not recorded; OPDMS, Ottawa Preparation for Decision-Making Scale; ORS, Ottawa Regret Scale; PDSI, patient decision support intervention; PHQ, Patient Health Questionnaire; PROMIS PF, Patient-Reported Outcomes Measurement Information System Physical Function; PSS, Perceived Stress Scale; QALY, quality-adjusted life-year; QoL, quality of life; QuickDASH, Quick Disabilities of Arm, Shoulder, and Hand; RCT, randomized controlled trial; RIRS, retrograde intrarenal surgery; SDM-Q-9, Shared Decision-Making Questionnaire 9-item; SDQ, Subjective Decision Quality; SDS, Satisfaction with Decision Scale; SECPMD, Self-Efficacy to Communicate with Physician/Manage Disease; SF, Short-Form Health Survey; STAI, State-Trait Anxiety Inventory; SWD, Satisfaction with Decision Scale; SWDMP, Satisfaction with the Process of Making a Treatment Decision Scale; SWL, shock wave lithotripsy; Y, yes.

### Patient decision support interventions characteristics and quality

A detailed summary of the PDSIs interventions is provided in Supplementary Table 8 (Supplemental Digital Content 1, http://links.lww.com/JS9/A238). The PDSIs were primarily computer-based (*k*=26, 44.8%) or video-based (*k*=16, 27.6%), and used a digital platform (*k*=43, 74.1%). Some employed artificial intelligence (*k*=12, 20.3%) to predict postoperative PROs (*k*=5, 8.6%). The majority of the PDSIs utilized asynchronous communication mechanisms (*k*=50, 86.2%), were designed for self-administration by patients (*k*=45, 77.6%), incorporated patient’s values (*k*=39, 67.2%), and lacked a theoretical basis (*k*=40, 69.0%).

The evidence on the quality of PDSIs was available for all 58 trials (Supplementary Table 9, Supplemental Digital Content 1, http://links.lww.com/JS9/A238). Eleven PDSIs met all IPDASi-SF criteria, and their total score ranged from 5 to 16 (median=12). Full information on the available options, their positive and negative features, and fair comparisons, was provided for all PDSIs. However, incomplete information was provided regarding the impartial reviews (33 of 58 PDSIs; 56.9%), citations to referenced studies (32 of 58 PDSIs; 55.2%), testing details with patients (29 of 58 PDSIs; 50%), and production date (24 of 58 PDSIs; 41.4%). Ten trials incorporated the Ottawa Decision Support Framework (ODSF) to inform the design of the decision assistance, out of 18 trials that incorporated the theoretical framework.

### Risk of bias in included studies

Forty-eight trials (82.8%) were rated as having some concerns, six (10.3%) were rated to have low risk, and four (6.9%) were at high risk of overall bias (Supplementary Fig. 1, Supplemental Digital Content 1, http://links.lww.com/JS9/A238 and Supplementary Fig. 2, Supplemental Digital Content 1, http://links.lww.com/JS9/A238). The risks and concerns were mostly attributed to the absence of a published protocol to evaluate the selection of reported results, lack of information about the randomization process, deviations from the intended interventions, and measurement of the outcome with inadequate masking of the outcome assessors. All RCTs adequately addressed the issue of missing outcome data. Supplementary Figure 2 (Supplemental Digital Content 1, http://links.lww.com/JS9/A238) illustrates the risk of bias graph stratified by intention-to-treat and per-protocol analyses. The inter-rater agreement was almost perfect (κ=0.96, *P*<0.001).

### Effects of patient decision support interventions on invasive treatment choice and decision-making–related outcomes

Forty-two trials (72.4%) reported on the invasive treatment choice^51^–^53^,^55^,^58^,^59^,^61^,^64^–^69^,^72^–^78^,^80^,^82^–^85^,^87^,^89^–^91^,^95^–^98^,^100^–^107^. Of the 5136 patients who used PDSIs, 2396 (46.7%) chose the invasive treatment option, compared with 2302 of 4802 patients (47.9%) in the control groups. The absolute difference was 1.2% and RR was 0.97 (95% CI: 0.90, 1.04; *I* ^2^ *=*64.5%). A similar effect was observed in a follow-up assessment (median: 9 months) of treatment choice (Table 2, Supplementary Fig. 3, Supplemental Digital Content 1, http://links.lww.com/JS9/A238).

Thirty-nine trials reported on decision-making–related outcomes (Supplementary Fig. 4, Supplemental Digital Content 1, http://links.lww.com/JS9/A238), including 27 trials (46.6%) reporting on decisional conflict^50^–^52^,^54^,^56^,^57^,^60^,^61^,^64^,^71^–^74^,^76^,^78^–^80^,^83^,^84^,^86^,^98^,^99^,^101^,^103^,^104^,^107^, 18 (31.0%) on decisional satisfaction^57^,^60^,^63^,^66^,^67^,^70^,^72^,^73^,^77^,^78^,^80^,^84^,^86^,^98^,^103^–^105^, 15 (25.9%) on disease and treatment knowledge^50^,^52^,^60^,^73^,^74^,^77^–^80^,^84^,^89^,^95^,^98^,^104^–^106^, eight (13.8%) on decisional regret^51^,^70^,^72^,^74^,^76^,^84^,^86^,^104^, five (8.6%) on preparedness for decision-making^50^,^62^,^72^,^77^,^98^, four (6.9%) on decision quality measured through value concordance^62^,^66^,^89^,^107^, and three (5.2%) on shared decision-making^66^,^72^,^73^, decisional self-efficacy^52^,^73^,^105^, and outcome expectations^52^,^74^,^98^.

A small effect size (*g*) of PDSIs on decisional conflict (−0.29; 95% CI, −0.41, −0.16; *I* ^2^ *=*79.7%) was observed. The effect size at follow-up assessment (median: 6 months) was −0.11 (95% CI: −0.23, 0.01; *I* ^2^ *=*0.0%)^50^,^51^,^79^,^84^,^86^,^103^. The effect size ranged from −0.38 to −0.10 for subdomains of decisional conflict (Table 2).

**Table 2:** Effectiveness of PDSIs on invasive treatment choice, decision-making–related outcomes, PROs, and outcomes related to healthcare resource use

				Overall effect		Test of heterogeneity			
Outcomes	Number of trials (references)	Sample size	Effect estimate (95% CI)	*Z*	*P*	*I* ^2^ (%)	χ^2^	*P*	Certainty of evidence
Invasive treatment choice									
Postintervention	42^51^–^53^,^55^,^58^,^59^,^61^,^64^–^69^,^72^–^78^,^80^,^82^–^85^,^87^,^89^–^91^,^95^–^98^,^100^–^107^	9938	RR=0.97 (0.90, 1.04)	−0.83	0.41	64.5	115.35	<0.001	Very low
Follow-up (1–85 months)	6^51^,^72^,^84^,^92^,^100^,^102^	1429	RR=1.05 (0.85, 1.30)	0.46	0.65	82.2	28.15	<0.001	Very low
Decision-making–related									
Decisional conflict									
Postintervention	27^50^–^52^,^54^,^56^,^57^,^60^,^61^,^64^,^71^–^74^,^76^,^78^–^80^,^83^,^84^,^86^,^98^,^99^,^101^,^103^,^104^,^107^	5726	*g*=−0.29 (−0.41, −0.16)	−4.41	<0.001**	79.7	128.27	<0.001	Very low
Follow-up (1–6 months)	6^50^,^51^,^79^,^84^,^86^,^103^	1100	*g*=−0.11 (−0.23, 0.01)	−1.84	0.07	0.0	2.70	0.75	Low
Subscale of Decisional Conflict Scale									
Informed subscale	13^56^,^57^,^60^,^64^,^71^–^73^,^78^,^80^,^84^,^101^,^106^	3215	*g*=−0.38 (−0.61, −0.14)	−3.11	<0.001**	88.9	107.84	<0.001	Very low
Values clarity subscale	12^56^,^57^,^60^,^64^,^71^–^73^,^78^,^80^,^84^,^101^	2973	*g*=−0.25 (−0.41, −0.08)	−2.89	<0.001**	74.8	43.69	<0.001	Very low
Support subscale	12^56^,^57^,^60^,^64^,^71^–^73^,^78^,^80^,^84^,^101^	2903	*g*=−0.17 (−0.29, −0.04)	−2.65	0.01*	50.7	22.30	0.02	Very low
Uncertainty subscale	13^56^,^57^,^60^,^64^,^71^–^73^,^78^,^80^,^84^,^101^,^106^	3218	*g*=−0.10 (−0.17, −0.02)	−2.52	0.01*	5.6	12.71	0.39	Low
Effective decision subscale	13^56^,^57^,^60^,^64^,^71^–^73^,^78^,^80^,^84^,^101^,^106^	3212	*g*=−0.14 (−0.23, −0.04)	−2.80	0.01*	28.6	16.80	0.16	Very low
Satisfaction with decision-making									
Postintervention	18^57^,^60^,^63^,^66^,^67^,^70^,^72^,^73^,^77^,^78^,^80^,^84^,^86^,^98^,^103^–^105^	3744	*g*=0.09 (−0.05, 0.22)	1.26	0.21	71.7	60.0	<0.001	Very low
Follow-up (6 months)	4^84^,^86^,^103^,^104^	649	*g*=0.16 (0.01, 0.32)	2.09	0.04*	0.0	1.55	0.67	Moderate
Disease and treatment knowledge									
Postintervention	15^50^,^52^,^60^,^73^,^74^,^77^–^80^,^84^,^89^,^95^,^98^,^104^,^106^	4118	*g*=0.32 (0.15, 0.49)	3.65	<0.001**	84.6	90.98	<0.001	Very low
Follow-up (1–6 months)	4^50^,^52^,^79^,^84^	586	*g*=0.10 (−0.06, 0.27)	1.27	0.20	0.0	0.32	0.96	Moderate
Decisional regret									
Postsurgery (immediately–6 months)	8^51^,^70^,^72^,^74^,^76^,^84^,^86^,^104^	1043	*g*=−0.20 (−0.53, 0.13)	−1.21	0.23	83.6	42.68	<0.001	Very low
Follow-up (4–6 months)	3^51^,^74^,^104^	551	*g*=0.03 (−0.36, 0.42)	0.14	0.89	80.2	10.10	0.01	Low
Decision quality									
Postintervention	3^62^,^66^,^83^	745	*g*=0.53 (−0.02, 1.09)	1.88	0.06	90.5	20.95	<0.001	Very low
Values-concordance	4^62^,^66^,^89^,^107^	1712	RR=1.98 (1.15, 3.39)	2.48	0.01*	90.2	30.68	<0.001	Very low
Preparedness for decision-making	5^50^,^62^,^72^,^77^,^98^	953	*g*=0.22 (0.09, 0.34)	3.36	<0.001**	0.0	3.27	0.51	Low
Shared decision-making	3^66^,^72^,^73^	1527	*g*=0.22 (−0.31, 0.75)	0.80	0.42	88.7	17.76	<0.001	Very low
Decision self-efficacy	3^52^,^73^,^105^	1595	*g*=0.02 (−0.07, 0.12)	0.48	0.63	0.0	0.32	0.85	Moderate
Outcome expectations	3^52^,^74^,^98^	526	*g*=0.11 (−0.57, 0.80)	0.32	0.75	93.5	30.55	<0.001	Very low
PROs									
General HRQoL									
Postintervention	5^51^,^71^,^72^,^95^,^96^	862	*g*=0.02 (−0.20, 0.25)	0.21	0.84	58.3	9.59	0.05	Very low
Follow-up (3–9 months)	3^51^,^71^,^96^	461	*g*=0.13 (−0.18, 0.44)	0.84	0.40	61.3	5.17	0.08	Low
Physical health	2^70^,^71^	285	*g*=0.15 (−0.08, 0.38)	1.30	0.20	0.0	0.05	0.82	Moderate
Mental health									
Postintervention	2^71^,^74^	363	*g*=0.14 (−0.34, 0.62)	0.59	0.57	80.3	5.08	0.02	Low
Follow-up (postoperative–4 months)	2^71^,^74^	363	*g*=0.07 (−0.13. 0.28)	0.69	0.49	0.0	0.04	0.84	High
Condition-specific HRQoL	5^66^,^76^,^83^,^99^,^104^	379	*g*=0.28 (−0.11, 0.67)	1.39	0.17	67.8	12.4	0.01	Very low
Anxiety									
Postintervention	9^74^,^78^,^80^,^95^,^96^,^99^,^103^,^105^,^106^	1640	*g*=−0.03 (−0.20, 0.13)	−0.40	0.69	57.9	19.0	0.01	Very low
Follow-up (4–9 months)	3^74^,^96^,^103^	513	*g*=−0.05 (−0.25, 0.16)	−0.44	0.66	27.5	2.76	0.25	Moderate
Depression									
Postintervention	6^51^,^74^,^95^,^96^,^103^,^104^	1191	*g*=0.04 (−0.10, 0.17)	0.51	0.61	24.7	6.64	0.25	Moderate
Follow-up (4–9 months)	4^51^,^74^,^96^,^103^	749	*g*=−0.02 (−0.19, 0.14)	−0.27	0.78	24.0	3.95	0.27	High
Perceived stress	3^51^,^79^,^95^	729	*g*=−0.00 (−0.19, 0.19)	−0.00	1.00	40.6	3.37	0.19	High
Healthcare resources use									
Consultation time (min)	4^66^,^83^,^99^,^104^	362	MD=0.04 (−0.17, 0.24)	0.37	0.71	0.0	1.42	0.70	Moderate

*g*, Hedges’s *g*; HRQoL, health-related quality of life; *I* ^2^, percentage of variation across studies that is due to heterogeneity rather than chance; MD, mean difference; PDSI, patient decision support intervention; PRO, patient-reported outcomes; RR, risk ratio; *Z*, overall effect size (*Z*-statistics); χ^2^, Cochran’s *Q* test.

* *P*<0.05.

** *P*<0.001.

A negligible effect size of 0.09 (95% CI: −0.05, 0.22; *I* ^2^ *=*71.7%) was observed for PDSIs on satisfaction with decision-making. At a follow-up assessment (median: 6 months), the effect size was 0.16 (95% CI: 0.01, 0.32; *I* ^2^ *=*0.0%)^84^,^86^,^103^,^104^.

The effect size of PDSIs on disease and treatment knowledge was 0.32 (95% CI: 0.15, 0.49; *I* ^2^=84.6%). The effect size of PDSIs on knowledge was 0.10 (95% CI: −0.06, 0.27; *I* ^2^ *=*0.0%) at a follow-up assessment (median: 4.5 months) based on four trials^50^,^52^,^79^,^84^.

The effect size on decision-making preparedness was 0.22 (95% CI: 0.09, 0.34; *I* ^2^ *=*0.0%). The pooled estimate revealed that the patients who used PDSIs were more likely to have experienced better decision quality (RR=1.98; 95% CI: 1.15, 2.36; *I* ^2^ *=*90.2%) as compared with the control groups.

There were no significant changes in the following decision-making–related outcomes: decisional regret, shared decision-making, decision self-efficacy, and outcome expectations (Table 2).

### Effects of patient decision support interventions on patient-related outcomes and healthcare resource utilization

There were 18 trials that reported on PROs (Supplementary Fig. 5, Supplemental Digital Content 1, http://links.lww.com/JS9/A238), including nine trials that reported on anxiety^74^,^78^,^80^,^95^,^96^,^99^,^103^,^105^,^106^, six on depression^51^,^74^,^95^,^96^,^103^,^104^, five on general HRQoL^51^,^71^,^72^,^95^,^96^, five on condition-specific HRQoL^66^,^76^,^83^,^99^,^104^, and three on perceived stress^51^,^79^,^95^. The use of PDSIs had no effect on HRQoL (general or condition-specific), physical health, mental health, depression, anxiety, and perceived stress (Table 2).

Four trials^66^,^83^,^99^,^104^ assessed healthcare resource utilization in terms of consultation time but the overall effect was negligible (mean difference=0.04 min; 95% CI: −0.17, 0.24; *P*=0.71) (Supplementary Fig. 6, Supplemental Digital Content 1, http://links.lww.com/JS9/A238).

### Subgroup analysis

Subgroup analysis showed a statistically significant subgroup difference in decision conflict (χ^2^=12.80; *P*=0.03), with a very large effect size of −1.07 on reducing decisional conflict in patients considering breast reconstruction^76^,^78^,^83^,^86^,^99^ (*g*=−1.07; 95% CI: −1.91, −0.23), but no effect in patients considering cesarean section^73^,^80^ (*g*=−0.13; 95% CI: −0.38, 0.12) and those contemplating destination therapy left ventricular assist device (LVAD) placement^51^,^72^ (*g*=0.06; 95% CI: −0.17, 0.29). In addition, self-guided PDSIs (*g*=0.35; 95% CI: 0.19, 0.51) had a much greater effect on disease and treatment knowledge^50^,^52^,^60^,^74^,^77^–^80^,^84^,^89^,^95^,^98^,^104^,^106^ as compared with clinician-delivered PDSIs (*g*=0.00; 95% CI: −0.11, 0.11) with a subgroup difference (χ^2^=12.82; *P*<0.001). No other differences were observed in subgroup comparisons (Table 3 (Supplementary Figs. 7–10, Supplemental Digital Content 1, http://links.lww.com/JS9/A238).

**Table 3:** Subgroup analysis of the PDSIs on invasive treatment choice and decision-making–related outcomes

			Test of heterogeneity			
Variables	Number of trials (references)	Pooled estimate (95% CI)	*I* ^2^ (%)	χ^2^	*P*	χ^2^, subgroup differences
Invasive treatment choice						
Types of elective surgery						
Breast reconstruction	3^76^,^78^,^83^	RR=0.99 (0.83, 1.18)	0.0	1.26	0.53	14.06, *P*=0.08
Destination therapy LVAD placement	2^51^,^72^	RR=0.77 (0.51, 1.15)	80.0	4.99	0.03	
First/repeat cesarean section	4^59^,^73^,^80^,^87^	RR=0.99 (0.87, 1.14)	41.8	5.15	0.16	
Lumbar spine surgery	2^58^,^82^	RR=0.70 (0.49, 1.01)	19.9	1.25	0.26	
Lumpectomy and/or mastectomy	8^67^,^74^,^77^,^85^,^91^,^101^,^103^,^105^	RR=1.13 (0.88, 1.46)	59.2	17.14	0.02	
Prophylactic mastectomy/oophorectomy/hysterectomy	5 69,95,96,102	RR=1.12 (1.01, 1.24)	0.0	1.53	0.82	
Prostatectomy/radical prostatectomy or orchidectomy	3^53^,^75^,^97^	RR=0.91 (0.65, 1.26)	86.7	15.03	<0.001	
Total knee/hip/joint replacement/arthroplasty	6^55^,^65^,^66^,^84^,^89^,^100^	RR=1.01 (0.89, 1.15)	52.7	10.57	0.06	
Others^a^	9^52^,^61^,^64^,^68^,^90^,^98^,^104^,^106^,^107^	RR=0.87 (0.73, 1.03)	53.5	17.19	0.03	
Mode of delivery						
Clinician-delivered	11^51^,^53^,^66^,^67^,^72^,^73^,^90^,^96^,^97^,^101^,^103^	RR=0.88 (0.73, 1.07)	79.2	48.16	<0.001	1.33, *P*=0.25
Self-guided	31^52^,^55^,^58^,^59^,^61^,^64^,^65^,^68^,^69^,^74^–^78^,^80^,^82^–^85^,^87^,^89^,^91^,^95^,^98^,^100^,^102^,^104^–^107^	RR=1.00 (0.93, 1.07)	52.9	63.74	<0.001	
Consider value						
Yes	26^51^,^59^,^64^–^69^,^73^–^78^,^80^,^83^–^85^,^87^,^89^,^90^,^96^,^97^,^104^,^106^,^107^	RR=0.98 (0.90, 1.07)	60.6	63.39	<0.001	0.27, *P*=0.61
No	16^52^,^53^,^55^,^58^,^61^,^72^,^82^,^91^,^95^,^98^,^100^–^103^,^105^	RR=0.94 (0.83, 1.07)	71.1	51.95	<0.001	
Use of theoretical framework						
Yes	12^51^,^59^,^61^,^64^,^67^,^72^,^77^,^78^,^85^,^87^,^98^,^104^	RR=0.98 (0.83, 1.17)	61.0	28.23	<0.001	0.03, *P*=0.87
No	30^52^,^53^,^55^,^58^,^65^,^66^,^68^,^69^,^73^–^76^,^80^,^82^–^84^,^89^–^91^,^95^–^97^,^100^–^103^,^105^–^107^	RR=0.97 (0.89, 1.05)	66.6	86.73	<0.001	
PROM-based PDSIs						
Yes	3^66^,^90^,^96^	RR=1.24 (0.85, 1.81)	0.0	1.64	0.44	1.64, *P*=0.20
No	39^51^–^53^,^55^,^58^,^59^,^61^,^64^–^69^,^72^–^78^,^80^,^82^–^85^,^87^,^89^,^91^,^95^,^97^,^98^,^100^–^107^	RR=0.96 (0.90, 1.04)	64.5	112.09	<0.001	
Decisional conflict						
Types of elective surgery						
Breast reconstruction	5^76^,^78^,^83^,^86^,^99^	*g*=−1.07 (−1.91, −0.23)	92.1	50.63	<0.001	12.80, *P*=0.03*
Destination therapy LVAD placement	2^51^,^72^	*g*=0.06 (−0.17, 0.29)	0.0	0.69	0.41	
First/repeat cesarean section	2^73^,^80^	*g*=−0.13 (−0.38, 0.12)	73.3	3.75	0.05	
Lumpectomy and/or mastectomy	4^60^,^74^,^101^,^103^	*g*=−0.31 (−0.46, −0.15)	0.0	1.33	0.72	
Total knee/hip/joint replacement/arthroplasty	4^50^,^57^,^84^	*g*=−0.48 (−0.96, 0.01)	86.6	22.31	<0.001	
Others^b^	10^52^,^54^,^56^,^61^,^64^,^71^,^79^,^98^,^104^,^107^	*g*=−0.16 (−0.29, −0.02)	53.5	19.37	0.02	
Mode of delivery						
Clinician-delivered	6^51^,^56^,^72^,^73^,^101^,^103^	*g*=−0.13 (−0.30, 0.03)	54.5	10.99	0.05	3.21, *P*=0.07
Self-guided	21^50^,^52^,^54^,^57^,^60^,^61^,^64^,^71^,^74^,^76^,^78^–^80^,^83^,^84^,^86^,^98^,^99^,^104^,^107^	*g*=−0.35 (−0.51, −0.18)	81.8	109.78	<0.001	
Consider value						
Yes	19^51^,^54^,^56^,^57^,^60^,^64^,^71^,^73^,^74^,^76^,^78^,^80^,^83^,^84^,^86^,^99^,^104^,^107^	*g*=−0.31 (−0.48, −0.15)	84.4	115.70	<0.001	0.26, *P*=0.61
No	8^50^,^52^,^61^,^72^,^79^,^98^,^101^,^103^	*g*=−0.26 (−0.40, −0.12)	28.1	9.73	0.20	
Use of theoretical framework						
Yes	10^51^,^54^,^61^,^64^,^72^,^78^,^79^,^86^,^98^,^104^	*g*=−0.29 (−0.54, −0.05)	81.7	49.14	<0.001	0.00, *P*=1.00
No	17^50^,^52^,^56^,^57^,^60^,^71^,^73^,^74^,^76^,^80^,^83^,^84^,^99^,^101^,^103^,^107^	*g*=−0.29 (−0.45, −0.14)	79.8	79.13	<0.001	
PROM-based PDSIs						
Yes	2^54^,^57^	*g*=−0.12 (−0.54, 0.29)	68.4	3.16	0.08	0.65, *P*=0.42
No	26^50^–^52^,^56^,^57^,^60^,^61^,^64^,^71^–^74^,^76^,^78^–^80^,^83^,^84^,^86^,^98^,^99^,^101^,^103^,^104^,^107^	*g*=−0.30 (−0.44, −0.17)	80.6	123.68	<0.001	
Satisfaction with decision-making						
Types of elective surgery						
Breast reconstruction	3^63^,^78^,^86^	*g*=0.09 (−0.16, 0.35)	31.6	2.92	0.23	0.20, *P*=1.00
First/repeat cesarean section	2^73^,^80^	*g*=0.13 (−0.25, 0.51)	91.6	11.86	<0.001	
Lumpectomy and/or mastectomy	5^60^,^67^,^77^,^103^,^105^	*g*=0.05 (−0.19, 0.30)	54.2	8.74	0.07	
Total knee/hip/joint replacement/arthroplasty	4^57^,^66^,^84^	*g*=0.14 (−0.34, 0.61)	85.8	21.12	<0.001	
Others^c^	4^70^,^72^,^98^,^104^	*g*=0.05 (−0.33, 0.43)	74.1	11.56	0.01	
Mode of delivery						
Clinician-delivered	6^66^,^67^,^70^,^72^,^73^,^103^	*g*=0.20 (−0.05, 0.44)	76.1	20.89	<0.001	1.23, *P*=0.27
Self-guided	12^57^,^60^,^63^,^77^,^78^,^80^,^84^,^86^,^98^,^104^,^105^	*g*=0.03 (−0.16, 0.21)	71.6	38.69	<0.001	
Consider value						
Yes	13^57^,^60^,^66^,^67^,^70^,^73^,^77^,^78^,^80^,^84^,^86^,^104^	*g*=0.11 (−0.06, 0.27)	74.3	46.75	<0.001	0.16, *P*=0.69
No	5^63^,^72^,^98^,^103^,^105^	*g*=0.04 (−0.25, 0.33)	69.5	13.10	0.01	
Use of theoretical framework						
Yes	8^67^,^70^,^72^,^77^,^78^,^86^,^98^,^104^	*g*=0.02 (−0.19, 0.23)	58.2	16.76	0.02	0.57, *P*=0.45
No	10^57^,^60^,^63^,^66^,^73^,^80^,^84^,^103^,^105^	*g*=0.13 (−0.06, 0.32)	79.0	42.89	<0.001	
PROM-based PDSIs						
Yes	2^57^,^66^	*g*=0.23 (−0.61, 1.06)	89.6	9.57	<0.001	0.13, *P*=0.72
No	17^57^,^60^,^63^,^67^,^70^,^72^,^73^,^77^,^78^,^80^,^84^,^86^,^98^,^103^–^105^	*g*=0.07 (−0.06, 0.21)	68.8	48.03	<0.001	
Disease and treatment knowledge						
Types of elective surgery						
First/repeat cesarean section	2^73^,^80^	*g*=0.28 (−0.28, 0.83)	95.9	21.11	<0.001	3.90, *P*=0.42
Lumpectomy and/or mastectomy	4^60^,^74^,^77^,^105^	*g*=0.15 (−0.04, 0.34)	17.2	3.62	0.31	
Prophylactic mastectomy/oophorectomy/hysterectomy	2^79^,^95^	*g*=0.20 (−0.16, 0.56)	72.2	3.59	0.06	
Total knee/hip/joint replacement/arthroplasty	3^50^,^84^,^89^	*g*=0.39 (0.18, 0.61)	46.8	3.76	0.15	
Others^d^	4^52^,^78^,^98^,^106^	*g*=0.50 (0.06, 0.94)	85.4	20.48	<0.001	
Mode of delivery						
Clinician-delivered	1^73^	*g*=0.00 (−0.11, 0.11)	–	–	–	12.82, *P*<0.001**
Self-guided	14^50^,^52^,^60^,^74^,^77^–^80^,^84^,^89^,^95^,^98^,^104^,^106^	*g*=0.35 (0.19, 0.51)	76.0	54.13	<0.001	
Consider value						
Yes	9^60^,^73^,^74^,^77^,^78^,^80^,^84^,^89^,^106^	*g*=0.39 (0.13, 0.65)	90.3	82.82	<0.001	1.15, *P*=0.28
No	6^50^,^52^,^79^,^95^,^98^,^104^	*g*=0.22 (0.07, 0.38)	36.7	7.89	0.16	
Use of theoretical framework						
Yes	4^77^–^79^,^98^	*g*=0.20 (−0.01, 0.41)	16.4	3.59	0.31	0.98, *P*=0.32
No	11^50^,^52^,^60^,^73^,^74^,^80^,^84^,^89^,^95^,^104^,^106^	*g*=0.35 (0.14, 0.56)	88.5	86.77	<0.001	

*g*, Hedges’s *g*; *I* ^2^, percentage of variation across studies that is due to heterogeneity rather than chance; LVAD, left ventricular assist devices; PDSI, patient decision support intervention; PROM, patient-reported outcome measure; RCT, randomized controlled trial; RR, risk ratio.

^a^Bariatric surgery, cataract surgery, aneurysm repair, lung transplantation, surgical termination of pregnancy, shock wave lithotripsy, and retrograde intrarenal surgery.

^b^Bariatric, cataract, general surgery, lung transplantation, percutaneous coronary intervention, prophylactic mastectomy, prostatectomy, shock wave lithotripsy, and retrograde intrarenal surgery.

^c^LVAD placement, lung transplantation, and general surgery.

^d^Bariatric surgery, lung transplantation, and surgical termination of pregnancy.

*P<0.05.

**P<0.001.

### Publication bias

Publication bias was not detected for treatment choice, patient satisfaction, and disease and treatment knowledge; however, there was an asymmetrical distribution on the funnel plots for decisional conflict (Supplementary Figs. 11a, c, d, Supplemental Digital Content 1, http://links.lww.com/JS9/A238). The trim-and-fill method imputed three studies and pooled confounder-adjusted estimate increased from −0.29 (95% CI: −0.41, −0.16) to −0.21 (95% CI: −0.35, −0.07) (Supplementary Fig. 12, Supplemental Digital Content 1, http://links.lww.com/JS9/A238). A sensitivity analysis using the Copas selection model suggested that publication bias was unlikely to be an issue (Supplementary Fig. 13, Supplemental Digital Content 1, http://links.lww.com/JS9/A238).

### Certainty of evidence

Supplementary Table 10 (Supplemental Digital Content 1, http://links.lww.com/JS9/A238) shows the GRADE summary of evidence. For all primary and secondary outcomes, the certainty of evidence was rated as very low or low, except for disease and treatment knowledge (follow-up), decision self-efficacy, physical health, anxiety (follow-up), depression (postintervention), and consultation time, which were rated as moderate, while mental health and depression (follow-up), as well as perceived stress, which were rated as high.

## Discussion

### Summary of evidence

Our review demonstrated PDSIs that were intended to guide decision-making for elective surgeries had a beneficial impact on many decision-related outcomes. These effects were small and varied according to the type of elective surgery and mode of delivery of the PDSIs. They did not influence invasive treatment choice, PROs, or healthcare utilization outcomes.

### Effects on outcome measures

**Figure 2:**
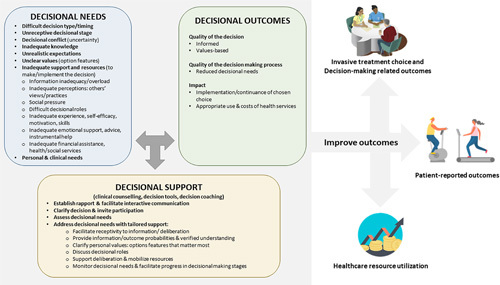
Possible mechanisms of patient decision support interventions in improving outcomes among elective surgical patients. Adapted from Ottawa Decision Support Framework.^109^

Many findings in this analysis corroborated with aggregated findings from previous reviews such as: using PDSIs reduces decisional conflict^7^–^9^, enhances disease and treatment knowledge^7^–^9^, and improves decision quality^8^. According to the ODSF^108^ (Fig. 2), PDSIs can assist in meeting decisional needs by providing information on the possible treatment options and health conditions, as well as the associated benefits and harms^5^. This enables patients to appreciate the value-sensitive nature of decisions, thus enhancing the preference elicitation process^5^. ODSF theorizes that when adequate decisional support meets decisional needs, decision quality improves with a greater possibility of value concordance^108^. Nonetheless, a comprehensive needs assessment is required before the implementation of PDSIs in a specific patient population, as our subgroup analysis indicated that the magnitude of the benefits may vary across patient populations and PDSIs designs.

In line with previous systematic reviews, our meta-analyses demonstrated that PDSIs had no effects on PROs^7^,^9^. In theory, the use of PDSIs may improve PROs through two mechanisms: (1) encouraging the selection of treatment with greater PROs benefits, and (2) improving an individual’s psychological well-being. The first mechanism contradicts our finding, which has shown that the use of PDSIs had no effects on treatment choice for most elective surgeries. This is because, in addition to the potential improvement in their PROs, the surgical candidate may also assess the risk of surgical adverse events. The second mechanism could work via enhanced shared decision-making^5^,^109^ or decisions aligned with the patients’ values and preferences. The insignificant effects on shared decision-making in this review contradicted the former; and the absence of trials that assessed positive psychological constructs such as satisfaction with treatment outcomes rendered the latter uncertain. Hence, future high-quality research is warranted to investigate the downstream effects of PDSIs, such as the positive psychological effects on treatment outcomes.

A recent Cochrane review^5^ found that the use of PDSIs had no discernible effect on the choice of nonsurgical or invasive surgical intervention, whereas another review^7^ found a marginal difference in which patients who used PDSIs were less likely to undergo surgical treatment. In line with the former review^5^, which focused exclusively on RCTs, our meta-analysis demonstrated no effect on the choice of invasive treatment. There are two plausible explanations for the contradictory result in the latter review^7^. First, the review combined experimental and observational studies, which increased the likelihood of bias. The analysis might also have overestimated the effect size and reported marginal effect due to the relatively smaller sample size (*N*=2674), as compared with our review (*N*=9938). Second, our review included trials with more diverse populations. PDSIs typically had a variable effect depending on the target population and the surgery being considered. Indeed, our subgroup analysis found that, while PDSIs had no influence on most elective surgery, they might be able to decrease the likelihood of some invasive procedures, most notably destination therapy LVAD placement and lumbar spine surgery, which is consistent with previous review findings^8^. Therefore, although the overall effect of PDSIs on treatment choice was largely minimal, it varied with the type of surgery.

Consistent with another meta-analytic review^5^, our review indicated that the use of PDSIs did not incur increased use of the surgeon’s consultation time. This would imply no increase in resource utilization and the likelihood of acceptance by clinicians if PDSIs were to be implemented in clinical practice. It should be noted that the timing of PDSIs administration might affect consultation time. Two included trials^83^,^99^, in which patients received a self-guided PDSIs a few days before the consultation, observed a shorter consultation time (*g*=−0.06; 95% CI: −0.38, 0.26). In contrast, two other trials^66^,^104^, in which patients received a self-guided PDSIs during the waiting time before the routine consultation, observed similar or even a longer consultation time (*g*=0.10; 95% CI: −0.16, 0.37) compared with the control group.

### Effects of investigator moderators

Surprisingly, our review discovered that self-guided PDSIs appeared to be more effective than clinician-administered PDSIs in enhancing disease and treatment knowledge and possibly reducing decisional conflict. This finding could be due to several reasons. First, providing self-guided PDSIs well before the consultation allows patients more time to digest the information and prepare for discussing the decision^5^. Second, there may be a lack of clinician buy-in for clinician-administered PDSIs, resulting in less effectiveness. A qualitative study among oncological surgeons showed that although two-thirds of them were aware of PDSIs, less than half had used one during routine surgical consultations^111^. Lastly, it could be due to chance because our subgroup analysis included one trial.

Similarly, theory-guided development, value consideration, or provision of patient-reported outcome measure data showed no effects in our review. Increasingly, PDSIs are developed by taking into account the recommendations of ODSF, which postulates that high-quality decisions are typically those consistent with the patient’s values^108^. A recent review stressed the need of including longitudinal PROs into the treatment decision-making process^112^. PROs are particularly relevant for patients considering elective surgeries as the main aim of the treatment is to improve functioning and well-being, or HRQoL. Given that PROs data is increasingly collected in clinical practice, incorporating such data into PDSIs becomes feasible. A possible reason for the insignificant subgroup differences of the design-related factors is that their effects were confounded or moderated by other contextual or implementation-related factors such as suboptimal protocol compliance. It is also possible that the design of PDSIs was inadequate or difficult for the user to comprehend. For example, it appears that the three PDSIs provided PROs data in the form of numerical scale scores without interpretation. Without training in psychometrics, patients are unlikely to be able to fully understand such information.

### Limitations

Several limitations should be considered before interpreting these findings. First, while the comprehensive search approach lends credibility to this review, we used a broad search strategy and selected a large amount of data. Although two independent reviewers were involved, reviewer’s fatigue might have led to the misclassification of records for inclusion. Second, the included trials were clinically and statistically heterogeneous, limiting their comparison. To address this issue, our study used subgroup analysis. Last, the English language restriction imposed on the RCTs might have limited the generalizability of the findings.

### Implications for future research and patient decision support interventions design

In this review, most trials were classified as having some concerns due to the lack of a published protocol for assessing bias in the selection of reported outcomes. In addition, a few trials were rated as having high risk of bias for not blinding those receiving the intervention and not providing the randomization procedure. Hence, investigators assessing the efficacy of PDSIs in future trials should adhere to good trial design as well as reporting standards such as the Consolidated Standards of Reporting Trials (CONSORT) 2010^113^.

Our review identified a significant gap in the reporting of PDSIs evaluation, including information about impartial review, citations to studies, and patient pilot testing. This made it challenging for reviewers to assess the quality of the PDSIs. Future research should develop and use a standardized International Patient Decision Aid Standards Version^26^ so that the quality of PDSIs can be properly assessed. In addition, it is difficult to explore the information in the comparator group due to a lack of description. Future studies are recommended to comply with the Standards for UNiversal reporting of patient Decision Aid Evaluation (SUNDAE) checklist to ensure transparent and high-quality reports of PDSIs evaluation studies^13^.

## Conclusions

This review has demonstrated that PDSIs targeting individuals considering elective surgeries had benefited their decision-making by reducing decisional conflict and increasing disease and treatment knowledge, decision-making preparedness, and decision quality. However, the quality of PDSIs varied and the certainty of evidence for many key outcomes was low. Nonetheless, these findings may be used to guide the development and evaluation of new PDSIs for use in elective surgical care. Furthermore, future high-quality research is needed to investigate the downstream treatment outcomes of PDSIs, such as the positive psychological effects of PDSIs.

## Ethical approval

Not applicable as this is a review paper.

## Sources of funding

None.

## Author contribution

L.J.C.: conceptualization, methodology, software, validation, formal analysis, investigation, resources, data curation, writing – original draft, writing – review and editing, visualization, and project administration. N.B.: methodology, validation, formal analysis, investigation, resources, writing – review and editing, and visualization. M.L.: methodology, validation, formal analysis, investigation, writing – review and editing, and visualization. V.X.W., W.W., G.K.P.L., and H.W.D.H.: methodology, validation, investigation, writing – review and editing, and supervision. N.L.: conceptualization, methodology, validation, investigation, resources, data curation, writing – review and editing, visualization, and supervision.

## Conflicts of interest disclosure

The authors declare that they have no financial conflict of interest with regard to the content of this report.

## Research registration unique identifying number (UIN)

1. Name of the registry: PROSPERO
2. Unique Identifying number or registration ID: CRD42021273767
3. Hyperlink to your specific registration (must be publicly accessible and will be checked): https://www.crd.york.ac.uk/prospero/display_record.php?RecordID=273767

## Guarantor

Nan Luo affirms that the manuscript is an honest, accurate, and transparent account of the study being reported; that no important aspects of the study have been omitted; and that any discrepancies from the study as planned (and, if relevant, registered) have been explained.

## Data availability statement

The authors confirm that the data supporting the findings of this study are available within the article [and/or] its supplementary materials.

## Provenance and peer review

Not commissioned, externally peer-reviewed.

## Supplementary Material
