# Supplementary material for: Patient decision support interventions for candidates considering elective surgeries: a systematic review and meta-analysis
Source: Int J Surg. 2023 Apr 10;109(5):1382–99. doi: 10.1097/JS9.0000000000000302 (PMC10389624; doi:10.1097/JS9.0000000000000302)
Supplement: Supplementary file 1 [file js9-109-1382-s001.docx]

**Table of Contents**

[**Supplementary Table 1** PRISMA 2020 Checklist statement. 2](#_Toc124625943)

[**Supplementary Table 2** Eligibility criteria. 6](#_Toc124625944)

[**Supplementary Table 3** Index and keyword terms used in the databases. 7](#_Toc124625945)

[**Supplementary Table 4** Lists of clinical trial registries, specialised journal, and grey literature databases. 14](#_Toc124625946)

[**Supplementary Table 5** List of excluded studies. 15](#_Toc124625947)

[**Supplementary Table 6** Outcomes and their respective definition and measures. 23](#_Toc124625948)

[**Supplementary Table 7** Study code and references for all included studies. 26](#_Toc124625949)

[**Supplementary Table 8** Description of PDSIs in selected trials. 32](#_Toc124625950)

[**Supplementary Table 9** The International Patient Decision Aid Standards Instrument “Short Form” (IPDASi-SF) Ratings. 42](#_Toc124625951)

[**Supplementary Table 10** GRADE summary of evidence for PDSIs on decision-making related, patient-reported outcomes and healthcare resources outcomes. 44](#_Toc124625952)

[**Supplementary Figure 1.** Risk of bias summary. 51](#_Toc124625953)

[**Supplementary Figure 2.** Risk of bias graph stratified by (a) intention-to-treat and (b) per-protocol analysis. 52](#_Toc124625954)

[**Supplementary Figure 3.** Forest plot of risk ratio (95% CI) for PDSIs and comparator groups on actual invasive treatment choice 53](#_Toc124625955)

[**Supplementary Figure 4.** Forest plot of Hedges’s *g* (95% CI) for PDSIs and comparator groups on decision-making related outcomes 55](#_Toc124625956)

[**Supplementary Figure 5.** Forest plot of Hedges’s *g* (95% CI) for PDSIs and comparator groups on patient-reported outcomes 65](#_Toc124625957)

[**Supplementary Figure 6.** Forest plot of Hedges’s *g* (95% CI) for PDSIs and comparator groups on healthcare resources use outcomes 69](#_Toc124625958)

[**Supplementary Figure 7.** Forest plot of risk ratio (95% CI) for PDSIs and comparator groups on actual invasive treatment choice, stratified by different modifiers 70](#_Toc124625959)

[**Supplementary Figure 8.** Forest plot of Hedges’s *g* (95% CI) for PDSIs and comparator groups on decisional conflict, stratified by different modifiers 75](#_Toc124625960)

[**Supplementary Figure 9.** Forest plot of Hedges’s *g* (95% CI) for PDSIs and comparator groups on patient satisfaction, stratified by different modifiers 80](#_Toc124625961)

[**Supplementary Figure 10.** Forest plot of Hedges’s *g* (95% CI) for PDSIs and comparator groups on disease and treatment knowledge, stratified by different modifiers 85](#_Toc124625962)

[**Supplementary Figure 11.** Egger's regression test, Begg’s test and funnel plots of precision by Hedges’s g of (a) invasive treatment choice (N = 42), (b) decisional conflict (N = 27), (c) satisfaction with decision-making (N = 18), and (d) disease and treatment knowledge outcome (N = 15). 89](#_Toc124625963)

[**Supplementary Figure 12.** Nonparametric trim-and-fill analysis of publication bias for decisional conflict outcome. 90](#_Toc124625964)

[**Supplementary Figure 13.** Funnel, contour, treatment effect and *p*-value for residual selection bias plot. 91](#_Toc124625965)

# **Supplementary Table 1** PRISMA 2020 Checklist statement.

| **Section and Topic** | **Item #** | **Checklist item** | **Location where item is reported** |
| --- | --- | --- | --- |
| **TITLE** | | |  |
| Title | 1 | Identify the report as a systematic review. | 1 |
| **ABSTRACT** | | |  |
| Abstract | 2 | See the PRISMA 2020 for Abstracts checklist. | 1-2 |
| **INTRODUCTION** | | |  |
| Rationale | 3 | Describe the rationale for the review in the context of existing knowledge. | 3-4 |
| Objectives | 4 | Provide an explicit statement of the objective(s) or question(s) the review addresses. | 3-4 |
| **METHODS** | | |  |
| Eligibility criteria | 5 | Specify the inclusion and exclusion criteria for the review and how studies were grouped for the syntheses. | 4, Supplementary Table 2 |
| Information sources | 6 | Specify all databases, registers, websites, organisations, reference lists and other sources searched or consulted to identify studies. Specify the date when each source was last searched or consulted. | 4-5, Figure 1 |
| Search strategy | 7 | Present the full search strategies for all databases, registers and websites, including any filters and limits used. | 4-5, Supplementary Table 3 |
| Selection process | 8 | Specify the methods used to decide whether a study met the inclusion criteria of the review, including how many reviewers screened each record and each report retrieved, whether they worked independently, and if applicable, details of automation tools used in the process. | 5-6 |
| Data collection process | 9 | Specify the methods used to collect data from reports, including how many reviewers collected data from each report, whether they worked independently, any processes for obtaining or confirming data from study investigators, and if applicable, details of automation tools used in the process. | 5-6 |
| Data items | 10a | List and define all outcomes for which data were sought. Specify whether all results that were compatible with each outcome domain in each study were sought (e.g. for all measures, time points, analyses), and if not, the methods used to decide which results to collect. | 5-6, Supplementary Table 6 |
|  | 10b | List and define all other variables for which data were sought (e.g. participant and intervention characteristics, funding sources). Describe any assumptions made about any missing or unclear information. | 5-6 |
| Study risk of bias assessment | 11 | Specify the methods used to assess risk of bias in the included studies, including details of the tool(s) used, how many reviewers assessed each study and whether they worked independently, and if applicable, details of automation tools used in the process. | 7 |
| Effect measures | 12 | Specify for each outcome the effect measure(s) (e.g. risk ratio, mean difference) used in the synthesis or presentation of results. | 7-8 |
| Synthesis methods | 13a | Describe the processes used to decide which studies were eligible for each synthesis (e.g. tabulating the study intervention characteristics and comparing against the planned groups for each synthesis (item #5)). | 7-8 |
|  | 13b | Describe any methods required to prepare the data for presentation or synthesis, such as handling of missing summary statistics, or data conversions. | 7-8 |
|  | 13c | Describe any methods used to tabulate or visually display results of individual studies and syntheses. | 7-8 |
|  | 13d | Describe any methods used to synthesize results and provide a rationale for the choice(s). If meta-analysis was performed, describe the model(s), method(s) to identify the presence and extent of statistical heterogeneity, and software package(s) used. | 7-8 |
|  | 13e | Describe any methods used to explore possible causes of heterogeneity among study results (e.g. subgroup analysis, meta-regression). | 7-8 |
|  | 13f | Describe any sensitivity analyses conducted to assess robustness of the synthesized results. | 7-8 |
| Reporting bias assessment | 14 | Describe any methods used to assess risk of bias due to missing results in a synthesis (arising from reporting biases). | 8 |
| Certainty assessment | 15 | Describe any methods used to assess certainty (or confidence) in the body of evidence for an outcome. | 8 |
| **RESULTS** | | |  |
| Study selection | 16a | Describe the results of the search and selection process, from the number of records identified in the search to the number of studies included in the review, ideally using a flow diagram. | 8-9, Supplementary Table 7 |
|  | 16b | Cite studies that might appear to meet the inclusion criteria, but which were excluded, and explain why they were excluded. | 8-9, Supplementary Table 5 |
| Study characteristics | 17 | Cite each included study and present its characteristics. | 9-10, Table 1, Supplementary Table 8-9 |
| Risk of bias in studies | 18 | Present assessments of risk of bias for each included study. | 10, Supplementary Figure 1-2 |
| Results of individual studies | 19 | For all outcomes, present, for each study: (a) summary statistics for each group (where appropriate) and (b) an effect estimate and its precision (e.g. confidence/credible interval), ideally using structured tables or plots. | 10-12, Table 1-2 |
| Results of syntheses | 20a | For each synthesis, briefly summarise the characteristics and risk of bias among contributing studies. | 10-12, Table 1-2 |
|  | 20b | Present results of all statistical syntheses conducted. If meta-analysis was done, present for each the summary estimate and its precision (e.g. confidence/credible interval) and measures of statistical heterogeneity. If comparing groups, describe the direction of the effect. | 10-12, Table 1-2 |
|  | 20c | Present results of all investigations of possible causes of heterogeneity among study results. | 12-13, Table 1-2 |
|  | 20d | Present results of all sensitivity analyses conducted to assess the robustness of the synthesized results. | 12-13, Table 1-2 |
| Reporting biases | 21 | Present assessments of risk of bias due to missing results (arising from reporting biases) for each synthesis assessed. | 13, Supplementary Figure 11-13 |
| Certainty of evidence | 22 | Present assessments of certainty (or confidence) in the body of evidence for each outcome assessed. | 13, Supplementary Table 11 |
| **DISCUSSION** | | |  |
| Discussion | 23a | Provide a general interpretation of the results in the context of other evidence. | 13-18 |
|  | 23b | Discuss any limitations of the evidence included in the review. | 17 |
|  | 23c | Discuss any limitations of the review processes used. | 17 |
|  | 23d | Discuss implications of the results for practice, policy, and future research. | 17-18 |
| **OTHER INFORMATION** | | |  |
| Registration and protocol | 24a | Provide registration information for the review, including register name and registration number, or state that the review was not registered. | 4, Title page |
|  | 24b | Indicate where the review protocol can be accessed, or state that a protocol was not prepared. | 4 |
|  | 24c | Describe and explain any amendments to information provided at registration or in the protocol. | N.A. |
| Support | 25 | Describe sources of financial or non-financial support for the review, and the role of the funders or sponsors in the review. | Title page |
| Competing interests | 26 | Declare any competing interests of review authors. | Title page |
| Availability of data, code and other materials | 27 | Report which of the following are publicly available and where they can be found: template data collection forms; data extracted from included studies; data used for all analyses; analytic code; any other materials used in the review. | Title page |

*From:*  Page MJ, McKenzie JE, Bossuyt PM, Boutron I, Hoffmann TC, Mulrow CD, et al. The PRISMA 2020 statement: an updated guideline for reporting systematic reviews. BMJ 2021;372:n71. doi: 10.1136/bmj.n71

For more information, visit: <http://www.prisma-statement.org/>

# **Supplementary Table 2** Eligibility criteria.

| Criteria | Inclusion | Exclusion |
| --- | --- | --- |
| Population | - All studies enrolling surgical candidates considering elective surgeries. - No restrictions will be placed on the types of surgery/disease. | - Adults with cognitive impairment (measured by Montreal Cognitive Assessment/ Mini-Mental State Exam) /psychiatric disorders (e.g., schizophrenia, bipolar, etc.) |
| Intervention | - All studies that randomize surgical candidates considering elective surgeries to a PDSIs treatment arm. - PDSIs are tools to provide patients and/or clinicians with information, risks, and benefits about treatment or other care-related decisions when no one treatment option is superior and non-surgical alternatives are available. |  |
| Comparison | - No restrictions will be placed on the type of usual care or active control that is provided. |  |
| Outcomes | Invasive treatment choice  Decision-making related outcomes including:   - Decisional conflict & subscale - Satisfaction with decision-making - Disease and treatment knowledge - Decisional regret - Preparedness for decision-making - Decision quality - Value concordance - Shared decision-making - Decision self-efficacy - Outcome expectations   Patient-reported outcomes including:   - General HRQoL - Condition-specific HRQoL - Physical and mental health - Depression and anxiety - Perceived stress   Healthcare resource utilization outcomes   - Consultation time |  |
| Type of design | - Any types of randomized controlled trials | - Non-experimental study, qualitative or reviews |
| Years of publication | - No limit |  |
| Publication type | - Published and unpublished trials | - Abstract only - Book chapter review - Letters - Editorials - Systematic review |
| Language | - English | - Non-English |

# **Supplementary Table 3** Index and keyword terms used in the databases.

| **Database (Search engine)** | **Index and keyword terms** |
| --- | --- |
| **MEDLINE (PubMed)** | "Decision Support Systems, Clinical"[Mesh] OR "Decision Support Techniques"[Mesh] OR "Decision Making, Computer-Assisted"[Mesh] OR "Decision Making"[Mesh] OR "Clinical Decision-Making"[Mesh] OR "Decision Making, Shared"[Mesh] OR ((Decision*[Title/Abstract]) AND (Aid*[Title/Abstract] OR Tool*[Title/Abstract] OR Support*[Title/Abstract] OR Instrument*[Title/Abstract] OR Technolog*[Title/Abstract] OR System*[Title/Abstract] OR Technique*[Title/Abstract] OR Program*[Title/Abstract] OR Algorithm*[Title/Abstract] OR Process*[Title/Abstract] OR Method*[Title/Abstract] OR Board*[Title/Abstract] OR Guide*[Title/Abstract])) OR Shared Decision Making[Title/Abstract] OR Treatment Decision[Title/Abstract] OR ((Patient [Title/Abstract]) AND (Deci*[Title/Abstract] OR Prefer*[Title/Abstract] OR Participat*[Title/Abstract] OR Select*[Title/Abstract] OR Educat*[Title/Abstract] OR Choice[Title/Abstract]))  **AND**  "Elective Surgical Procedures"[Mesh] OR ((Elective[Title/Abstract] OR Non-emergency[Title/Abstract] OR Nonemergency[Title/Abstract] OR Non-Urgent[Title/Abstract] OR Nonurgent[Title/Abstract] OR Scheduled[Title/Abstract] OR Prophylactic[Title/Abstract]) AND (Abdominal[Title/Abstract] OR Aesthetic[Title/Abstract] OR Bariatric[Title/Abstract] OR Breast[Title/Abstract] OR Cardiovascular[Title/Abstract] OR Cataract[Title/Abstract] OR Ear Nose and Throat[Title/Abstract] OR Endocrine[Title/Abstract] OR Eye[Title/Abstract] OR General[Title/Abstract] OR Geriatric[Title/Abstract] OR Head and Neck[Title/Abstract] OR Hip[Title/Abstract] OR Knee[Title/Abstract] OR Liver[Title/Abstract] OR Lung[Title/Abstract] OR Neuro*[Title/Abstract] OR Oral[Title/Abstract] OR Orthognathic[Title/Abstract] OR Orthop*[Title/Abstract] OR Pelvi*[Title/Abstract] OR Plastic[Title/Abstract] OR Prostat*[Title/Abstract] OR Spine[Title/Abstract] OR Thora*[Title/Abstract] OR Trapeziometacarpal[Title/Abstract] OR Urolog*[Title/Abstract]) AND (Surger*[Title/Abstract] OR Operative∗[Title/Abstract] OR Operation*[Title/Abstract] OR Surgical[Title/Abstract] OR Procedure*[Title/Abstract] OR Transplant[Title/Abstract] OR Reconstruct*[Title/Abstract])) OR Prostatectomy[Title/Abstract] OR ((Hip[Title/Abstract] OR Joint[Title/Abstract] OR Knee[Title/Abstract]) AND (Replacement[Title/Abstract] OR Arthroplasty[Title/Abstract])) OR Lumpectomy[Title/Abstract] OR Mastectomy[Title/Abstract] OR Oophorectomy[Title/Abstract] OR Hysterectomy[Title/Abstract] OR LVAD[Title/Abstract] OR Caesarean section[Title/Abstract] OR Aneurysm repair[Title/Abstract] OR Percutaneous coronary intervention[Title/Abstract] OR Lumbar spine surgery[Title/Abstract]  **AND**  (randomized controlled trial[pt] OR controlled clinical trial[pt] OR randomized controlled trials[mh] OR random allocation[mh] OR double-blind method[mh] OR single-blind method[mh] OR clinicatrial[pt] OR clinical trials[mh] OR clinical trial*[tw] OR ((singl*[tw] OR doubl*[tw] OR trebl*[tw] OR tripl*[tw]) AND (mask*[tw] OR blind*[tw])) OR rct[tiab] OR intervention*[tiab] OR "latin square"[tw] OR placebos[mh] OR placebo*[tw] OR random*[tw] OR research design[mh] OR comparative study[pt] OR evaluation studies[pt] OR follow-up studies[mh] OR prospective studies[mh] OR cross-over studies[mh] OR control[tw] OR controll*[tw] OR prospectiv*[tw] OR volunteer*[tw]) NOT (animals[mh] NOT humans[mh]) |
| **Cochrane Central Register of Controlled Trials (Ovid)** | MeSH descriptor: [Decision Support Systems, Clinical] explode all trees  MeSH descriptor: [Decision Support Techniques] explode all trees  MeSH descriptor: [Decision Making, Computer-Assisted] explode all trees  MeSH descriptor: [Decision Making] explode all trees  MeSH descriptor: [Clinical Decision-Making] explode all trees  MeSH descriptor: [Decision Making, Shared] explode all trees  (((Decision*) NEXT (Aid* OR Tool* OR Support* OR Instrument* OR Technolog* OR System* OR Technique* OR Program* OR Algorithm* OR Process* OR Method* OR Board* OR Guide*)) OR ("Shared Decision Making") OR ("Treatment Decision") OR ((Patient) NEXT (Deci* OR Prefer* OR Participat* OR Select* OR Educat* OR Choice))):ti,ab,kw  **AND**  MeSH descriptor: [Elective Surgical Procedures] explode all trees  (((Elective OR Non-emergency OR Nonemergency OR Non-Urgent OR Nonurgent OR Scheduled OR Prophylactic) NEXT (Abdominal OR Aesthetic OR Bariatric OR Breast OR Cardiovascular OR Cataract OR "Ear Nose and Throat" OR Endocrine OR Eye OR General OR Geriatric OR "Head and Neck" OR Hip OR Knee OR Liver OR Lung OR Neuro* OR Oral OR Orthognathic OR Orthop* OR Pelvi* OR Plastic OR Prostat* OR Spine OR Thora* OR Trapeziometacarpal OR Urolog*) NEXT (Surger* OR Operative∗ OR Operation* OR Surgical OR Procedure* OR Transplant OR Reconstruct*)) OR Prostatectomy OR ((Hip OR Joint OR Knee) NEXT (Replacement OR Arthroplasty)) OR Lumpectomy OR Mastectomy OR Oophorectomy OR Hysterectomy OR LVAD OR "Caesarean section" OR "Aneurysm repair" OR "Percutaneous coronary intervention" OR "Lumbar spine surgery"):ti,ab,kw |
| **Excerpta Medica Database (Elsevier)** | 'Clinical Decision Support System'/exp OR 'Decision Support System'/exp OR 'Decision Making'/exp OR 'Clinical Decision Making'/exp OR 'Shared Decision Making'/exp OR ((Decision*) NEAR/1 (Aid* OR Tool* OR Support* OR Instrument* OR Technolog* OR System* OR Technique* OR Program* OR Algorithm* OR Process* OR Method* OR Board* OR Guide*)) OR 'Shared Decision Making' OR 'Treatment Decision' OR ((Patient) NEXT (Deci* OR Prefer* OR Participat* OR Select* OR Educat* OR Choice)):ti,ab  **AND**  'Elective Surgery'/exp OR (((Elective OR 'Non Emergency' OR Nonemergency OR 'Non Urgent' OR Nonurgent OR Scheduled OR Prophylactic) NEAR/1 (Abdominal OR Aesthetic OR Bariatric OR Breast OR Cardiovascular OR Cataract OR "Ear Nose and Throat" OR Endocrine OR Eye OR General OR Geriatric OR "Head and Neck" OR Hip OR Knee OR Liver OR Lung OR Neuro* OR Oral OR Orthognathic OR Orthop* OR Pelvi* OR Plastic OR Prostat* OR Spine OR Thora* OR Trapeziometacarpal OR Urolog*) NEAR/1 (Surger* OR Operative∗ OR Operation* OR Surgical OR Procedure* OR Transplant OR Reconstruct*)) OR Prostatectomy OR ((Hip OR Joint OR Knee) NEAR/1 (Replacement OR Arthroplasty)) OR Lumpectomy OR Mastectomy OR Oophorectomy OR Hysterectomy OR LVAD OR 'Caesarean section' OR 'Aneurysm repair' OR 'Percutaneous coronary intervention' OR 'Lumbar spine surgery'):ti,ab  **AND**  'intervention study'/exp OR 'clinical trial'/exp OR 'study design'/exp OR 'Latin square design'/exp OR 'comparative study'/exp OR 'controlled study'/exp OR 'crossover procedure'/exp OR 'double blind procedure'/exp OR 'randomized controlled trial'/exp OR 'single blind procedure'/exp OR 'prospective study'/exp OR 'comparative study'/exp OR 'evaluation study'/exp OR 'crossover procedure'/exp OR intervention*:ab,ti OR rct:ab,ti OR random*:ab,ti OR factorial*:ab,ti OR crossover*:ab,ti OR ‘cross over*’:ab,ti OR placebo*:ab,ti OR comparative:ab,ti OR comparing:ab,ti OR ‘evaluation stud*’:ab,ti OR trial*:ab,ti OR control*:ab,ti OR prospective:ab,ti OR assign*:ab,ti OR allocat*:ab,ti OR volunteer*:ab,ti OR ((singl*:ab,ti OR doubl*:ab,ti OR trebl*:ab,ti OR tripl*:ab,ti) AND (mask*:ab,ti OR blind*:ab,ti)) NOT ('animal'/exp NOT 'human'/exp) |
| **Cumulative Index to Nursing and Allied Health Literature (EBSCO)** | (MM "Decision Support Systems, Clinical") OR MH ("Decision Support Techniques" OR "Decision Support Systems, Management" OR "Decision Making, Computer Assisted" OR "Decision Making, Shared" OR "Decision Making, Patient" OR "Decision Making, Clinical" OR "Decision Making") OR TI (((Decision*) N1 (Aid* OR Tool* OR Support* OR Instrument* OR Technolog* OR System* OR Technique* OR Program* OR Algorithm* OR Process* OR Method* OR Board* OR Guide*) OR Shared Decision Making OR Treatment Decision OR Patient Decision OR ((Patient) N1 (Deci* OR Prefer* OR Participat* OR Select* OR Educat* OR Choice))) OR AB (((Decision*) N1 (Aid* OR Tool* OR Support* OR Instrument* OR Technolog* OR System* OR Technique* OR Program* OR Algorithm* OR Process* OR Method* OR Board* OR Guide*) OR Shared Decision Making OR Treatment Decision OR Patient) N1(Deci* OR Prefer* OR Participat* OR Select* OR Educat* OR Choice)))  **AND**  (MH "Surgery, Elective") OR  TI ((Elective OR Non-emergency OR Nonemergency OR Non-Urgent OR Nonurgent OR Scheduled OR Prophylactic) N1 (Abdominal OR Aesthetic OR Bariatric OR Breast OR Cardiovascular OR Cataract OR "Ear Nose and Throat" OR Endocrine OR Eye OR General OR Geriatric OR "Head and Neck" OR Hip OR Knee OR Liver OR Lung OR Neuro* OR Oral OR Orthognathic OR Orthop* OR Pelvi* OR Plastic OR Prostat* OR Spine OR Thora* OR Trapeziometacarpal OR Urolog*) N1 Surger* OR Operative∗ OR Operation* OR Surgical OR Procedure* OR Transplant OR Reconstruct*) OR Prostatectomy OR ((Hip OR Joint OR Knee) N1 (Replacement OR Arthroplasty)) OR Lumpectomy OR Mastectomy OR Oophorectomy OR Hysterectomy OR LVAD OR "Caesarean section" OR "Aneurysm repair" OR "Percutaneous coronary intervention" OR "Lumbar spine surgery") OR AB ((Elective OR Non-emergency OR Nonemergency OR Non-Urgent OR Nonurgent OR Scheduled) N1 (Abdominal OR Aesthetic OR Bariatric OR Breast OR Cardiovascular OR Cataract OR "Ear Nose and Throat" OR Endocrine OR Eye OR General OR Geriatric OR "Head and Neck" OR Hip OR Knee OR Liver OR Lung OR Neuro* OR Oral OR Orthognathic OR Orthop* OR Pelvi* OR Plastic OR Prostat* OR Spine OR Thora* OR Trapeziometacarpal OR Urolog*) N1 Surger* OR Operative∗ OR Operation* OR Surgical OR Procedure* OR Transplant OR Reconstruct*) OR Prostatectomy OR ((Hip OR Joint OR Knee) N1 (Replacement OR Arthroplasty)) OR Lumpectomy OR Mastectomy OR Oophorectomy OR Hysterectomy OR LVAD OR "Caesarean section" OR "Aneurysm repair" OR "Percutaneous coronary intervention" OR "Lumbar spine surgery")  **AND**  MH ("Clinical Trials+" OR "Quantitative Studies" OR "Study Design+" OR "Random Assignment" OR "Evaluation Research" OR "Comparative Studies") OR (PT Clinical trial) OR (TX clini* N1 trial*) OR (TX ((singl* N1 blind*) OR (singl* N1 mask*)) OR TX ((doubl* N1 blind*) OR (doubl* N1 mask*)) OR TX ((tripl* N1 blind*) OR (tripl* N1 mask*))) OR (TX randomi* control*) OR ((TX random* allocat*) OR (TX allocat* random*)) OR (TX placebo*) OR (TX (waitlist* OR (wait* AND list*)) AND (control* OR group))) OR ((TX "treatment as usual") OR (TX tau)) OR (TX (control* N3 (trial* OR study OR studies OR group*))) OR TX (rct OR intervention* OR “latin square” OR prospectiv* OR volunteer OR follow or factorial* OR crossover* OR “cross over” OR comparative OR comparing OR evaluation stud*) |
| **Web of Science (Clarivate)** | TI = ("Decision Support Systems, Clinical" OR "Decision Support Techniques" OR "Decision Making, Computer-Assisted" OR "Decision Making" OR "Clinical Decision-Making" OR "Decision Making, Shared" OR ((Decision*) AND (Aid* OR Tool* OR Support* OR Instrument* OR Technolog* OR System* OR Technique* OR Program* OR Algorithm* OR Process* OR Method* OR Board* OR Guide*)) OR "Shared Decision Making" OR "Treatment Decision" OR ((Patient) AND (Deci* OR Prefer* OR Participat* OR Select* OR Educat* OR Choice)))  **AND**  TI = (( Elective OR Non-emergency OR Nonemergency OR Non-Urgent OR Nonurgent OR Scheduled OR Prophylactic) AND (Abdominal OR Aesthetic OR Bariatric OR Breast OR Cardiovascular OR Abdominal OR Aesthetic OR Bariatric OR Breast OR Cardiovascular OR Cataract OR "Ear Nose and Throat" OR Endocrine OR Eye OR General OR Geriatric OR "Head and Neck" OR Hip OR Knee OR Liver OR Lung OR Neuro* OR Oral OR Orthognathic OR Orthop* OR Pelvi* OR Plastic OR Prostat* OR Spine OR Thora* OR Trapeziometacarpal OR Urolog*) AND (Surger* OR Operative∗ OR Operation* OR Surgical OR Procedure* OR Transplant OR Reconstruct*) OR Prostatectomy OR ((Hip OR Joint OR Knee) AND (Replacement OR Arthroplasty)) OR Lumpectomy OR Mastectomy OR Oophorectomy OR Hysterectomy OR LVAD OR "Caesarean section" OR "Aneurysm repair" OR "Percutaneous coronary intervention" OR "Lumbar spine surgery")  **AND**  TI = (rct OR random* OR control* OR trial OR placebo* OR compar* OR group OR groups OR therapy OR treatment OR intervention OR "research design" OR comparative OR "evaluation stud*" OR "follow-up stud*" OR "prospective" OR "single blind" OR "double blind" OR "trebl* blind" OR "triple blind" OR factorial OR allocat* OR assign* OR volunteer* OR crossover OR "cross over") |
| **PsycINFO (Ovid)** | exp Decision Support Systems/ OR exp Decision Making/ OR Shared decision making.mp.  OR ((Decision$ AND (Aid$ OR Tool$ OR Support$ OR Instrument$ OR Technolog$ OR System$ OR Technique$ OR Program$ OR Algorithm$ OR Process$ OR Method$ OR Board$ OR Guide$)) OR 'Shared Decision Making' OR 'Treatment Decision' OR ((Patient) AND (Deci$ OR Prefer$ OR Participat$ OR Select$ OR Educat$ OR Choice))).ab,ti,tw.  **AND**  (((Elective OR "Non Emergency" OR Nonemergency OR "Non Urgent" OR Nonurgent OR Scheduled OR Prophylactic) AND (Abdominal OR Aesthetic OR Bariatric OR Breast OR Cardiovascular OR Abdominal OR Aesthetic OR Bariatric OR Breast OR Cardiovascular OR Cataract OR "Ear Nose and Throat" OR Endocrine OR Eye OR General OR Geriatric OR "Head and Neck" OR Hip OR Knee OR Liver OR Lung OR Neuro$ OR Oral OR Orthognathic OR Orthop$ OR Pelvi$ OR Plastic OR Prostat$ OR Spine OR Thora$ OR Trapeziometacarpal OR Urolog$) AND (Surger$ OR Operative$ OR Operation$ OR Surgical OR Procedure$ OR Transplant OR Reconstruct$)) OR Prostatectomy OR ((Hip OR Joint OR Knee) AND (Replacement OR Arthroplasty)) OR Lumpectomy OR Mastectomy OR Oophorectomy OR Hysterectomy OR LVAD OR "Caesarean section" OR "Aneurysm repair" OR "Percutaneous coronary intervention" OR "Lumbar spine surgery").ab,ti,tw.  **AND**  (rct OR random$ OR control$ OR trial OR placebo$ OR compar$ OR therapy OR treatment OR intervention$ OR research design OR comparative OR evaluation stud$ OR follow-up stud$ OR prospective OR single blind OR double blind OR trebl$ blind OR triple blind OR factorial OR allocat$ OR assign$ OR volunteer$ OR crossover OR cross over).ab,ti,tw. |
| **ProQuest Dissertations and Theses (ProQuest)** | mainsubject(Decision Support Systems, Clinical) OR mainsubject(Decision Support Techniques) OR mainsubject(Decision Making, Computer-Assisted) OR mainsubject(Decision Making) OR mainsubject(Clinical Decision-Making) OR mainsubject(Decision Making, Shared) OR ti(((Decision) AND (Aid OR Tool OR Support OR Instrument OR Technology OR System OR Technique OR Program OR Algorithm OR Process OR Method OR Board OR Guide)) OR Shared Decision Making OR Treatment Decision OR ((Patient) AND (Deci$ OR Prefer$ OR Participat$ OR Select$ OR Educat$ OR Choice))) OR ab(((Decision) AND (Aid OR Tool OR Support OR Instrument OR Technology OR System OR Technique OR Program OR Algorithm OR Process OR Method OR Board OR Guide)) OR Shared Decision Making OR Treatment Decision OR ((Patient) AND (Deci$ OR Prefer$ OR Participat$ OR Select$ OR Educat$ OR Choice)))  **AND**  mainsubject(Elective Surgical Procedures) OR ti((Elective OR Non-emergency OR Nonemergency OR Non-Urgent OR Nonurgent OR Scheduled OR Prophylactic) AND (Abdominal OR Aesthetic OR Bariatric OR Breast OR Cardiovascular OR Abdominal OR Aesthetic OR Bariatric OR Breast OR Cardiovascular OR Cataract OR "Ear Nose and Throat" OR Endocrine OR Eye OR General OR Geriatric OR "Head and Neck" OR Hip OR Knee OR Liver OR Lung OR Neuro$ OR Oral OR Orthognathic OR Orthop$ OR Pelvi$ OR Plastic OR Prostat$ OR Spine OR Thora$ OR Trapeziometacarpal OR Urolog$) AND (Surger$ OR Operative$ OR Operation$ OR Surgical OR Procedure$ OR Transplant OR Reconstruct$) OR Prostatectomy OR ((Hip OR Joint OR Knee) AND (Replacement OR Arthroplasty)) OR Lumpectomy OR Mastectomy OR Oophorectomy OR Hysterectomy OR LVAD OR "Caesarean section" OR "Aneurysm repair" OR "Percutaneous coronary intervention" OR "Lumbar spine surgery") OR ab((Elective OR Non-emergency OR Nonemergency OR Non-Urgent OR Nonurgent OR Scheduled OR Prophylactic) AND (Abdominal OR Aesthetic OR Bariatric OR Breast OR Cardiovascular OR Abdominal OR Aesthetic OR Bariatric OR Breast OR Cardiovascular OR Cataract OR "Ear Nose and Throat" OR Endocrine OR Eye OR General OR Geriatric OR "Head and Neck" OR Hip OR Knee OR Liver OR Lung OR Neuro$ OR Oral OR Orthognathic OR Orthop$ OR Pelvi$ OR Plastic OR Prostat$ OR Spine OR Thora$ OR Trapeziometacarpal OR Urolog$) AND (Surger$ OR Operative$ OR Operation$ OR Surgical OR Procedure$ OR Transplant OR Reconstruct$) OR Prostatectomy OR ((Hip OR Joint OR Knee) AND (Replacement OR Arthroplasty)) OR Lumpectomy OR Mastectomy OR Oophorectomy OR Hysterectomy OR LVAD OR "Caesarean section" OR "Aneurysm repair" OR "Percutaneous coronary intervention" OR "Lumbar spine surgery")  **AND**  ti(rct OR random$ OR control$ OR trial OR placebo$ OR compar$ OR group OR groups OR therapy OR treatment OR intervention OR "research design" OR comparative OR "evaluation stud$" OR "follow-up stud$" OR "prospective" OR "single blind" OR "double blind" OR "trebl$ blind" OR "triple blind" OR factorial OR allocat$ OR assign$ OR volunteer$ OR crossover OR "cross over") OR ab(rct OR random$ OR control$ OR trial OR placebo$ OR compar$ OR group OR groups OR therapy OR treatment OR intervention OR "research design" OR comparative OR "evaluation stud$" OR "follow-up stud$" OR "prospective" OR "single blind" OR "double blind" OR "trebl$ blind" OR "triple blind" OR factorial OR allocat$ OR assign$ OR volunteer$ OR crossover OR "cross over") |
| **Scopus (Elsevier)** | TITLE-ABS-KEY ( ( decision* ) W/1 ( aid* OR tool* OR support* OR instrument* OR technolog* OR system* OR technique* OR program* OR algorithm* OR process* OR method* OR board* OR guide* ) ) OR TITLE-ABS-KEY ( "Shared Decision Making" OR "Treatment Decision" OR ((Patient) AND (Deci* OR Prefer* OR Participat* OR Select* OR Educat* OR Choice)))  **AND**  TITLE-ABS-KEY (((Elective OR Non-emergency OR Nonemergency OR Non-Urgent OR Nonurgent OR Scheduled OR Prophylactic) W/1 (Abdominal OR Aesthetic OR Bariatric OR Breast OR Cardiovascular OR Abdominal OR Aesthetic OR Bariatric OR Breast OR Cardiovascular OR Cataract OR "Ear Nose and Throat" OR Endocrine OR Eye OR General OR Geriatric OR "Head and Neck" OR Hip OR Knee OR Liver OR Lung OR Neuro* OR Oral OR Orthognathic OR Orthop* OR Pelvi* OR Plastic OR Prostat* OR Spine OR Thora* OR Trapeziometacarpal OR Urolog*) W/1 (Surger* OR Operative∗ OR Operation* OR Surgical OR Procedure* OR Transplant OR Reconstruct*)) OR Prostatectomy OR ((Hip OR Joint OR Knee) AND (Replacement OR Arthroplasty)) OR Lumpectomy OR Mastectomy OR Oophorectomy OR Hysterectomy OR LVAD OR "Caesarean section" OR "Aneurysm repair" OR "Percutaneous coronary intervention" OR "Lumbar spine surgery")  **AND**  TITLE-ABS-KEY ( rct OR random* OR control* OR trial OR placebo* OR compar* OR therapy OR treatment OR intervention* OR research AND design OR comparative OR evaluation AND stud* OR follow-up AND stud* OR prospective OR single AND blind OR double AND blind OR trebl* AND blind OR triple AND blind OR factorial OR allocat* OR assign* OR volunteer* OR crossover OR cross AND over ) |

# **Supplementary Table 4** Lists of clinical trial registries, specialised journal, and grey literature databases.

| Sources | Website |
| --- | --- |
| ***Clinical trial registries:*** |  |
| Australian New Zealand Clinical Trials Registry | http://www.anzctr.org.au/ |
| CenterWatch | http://www.centerwatch.com/ |
| ClinicalTrials.gov | https://clinicaltrials.gov/ |
| Cochrane Central Register of Controlled Trials | https://www.cochranelibrary.com/central/about-central |
| EU Clinical Trials Register | https://www.clinicaltrialsregister.eu/ctr-search/search |
| Singapore Clinical Trials Register | https://www.hsa.gov.sg/clinical-trials/clinical-trials-register |
| UK Clinical Research Network: Portfolio Database | https://www.nihr.ac.uk/research-and-impact/nihr-clinical-research-network-portfolio/ |
|  |  |
| ***Specialized journals:*** |  |
| Annals of Surgery | https://journals.lww.com/annalsofsurgery/pages/default.aspx |
| BMC Medical Informatics and Decision Making | https://bmcmedinformdecismak.biomedcentral.com/ |
| British Journal of Surgery | https://bjssjournals.onlinelibrary.wiley.com/journal/13652168 |
| International Journal of Surgery | https://www.journals.elsevier.com/international-journal-of-surgery |
| JAMA Surgery | https://jamanetwork.com/journals/jamasurgery |
| Journal of the American College of Surgeons | https://www.journalacs.org/ |
| Journal of Vascular Surgery | https://www.jvascsurg.org/ |
| Medical Decision Making | https://journals.sagepub.com/home/mdm |
| Plastic and Reconstructive Surgery | https://journals.lww.com/plasreconsurg/pages/default.aspx |
| Surgery | https://www.journals.elsevier.com/surgery |
| The American Journal of Surgery | https://www.journals.elsevier.com/the-american-journal-of-surgery |
| World Journal of Surgery | https://www.springer.com/journal/268 |
|  |  |
| ***Grey literature resources:*** |  |
| CogPrints | http://cogprints.org/ |
| Google Scholar | http://scholar.google.com.sg/ |
| GreySource | http://www.greynet.org/greysourceindex.html |
| NYAM Grey Literature Report | http://www.greylit.org/ |
| OpenGrey: System for Information on Grey Literature in Europe | http://www.opengrey.eu/ |
| OpenMD.com | https://openmd.com/directory/allergy-immunology |
| Science.gov | http://www.science.gov/scigov/ |
| World Bank Publications | https://www.worldbank.org/en/research |

# **Supplementary Table 5** List of excluded studies.

| Year | Identification | Reference of excluded studies | Reasons |
| --- | --- | --- | --- |
| 2011 | Other methods | Au, A. H., Lam, W. W., Chan, M. C., Or, A. Y., Kwong, A., Suen, D., Wong, A. L., Juraskova, I., Wong, T. W., & Fielding, R. (2011). Development and pilot-testing of a Decision Aid for use among Chinese women facing breast cancer surgery. *Health Expect*, *14*(4), 405-416. <https://doi.org/10.1111/j.1369-7625.2010.00655.x> | Developmental paper |
| 2016 | Other methods | Cohan, J. N., Ozanne, E. M., Sewell, J. L., Hofer, R. K., Mahadevan, U., Varma, M. G., & Finlayson, E. (2016). A Novel Decision Aid for Surgical Patients with Ulcerative Colitis: Results of a Pilot Study. *Dis Colon Rectum*, *59*(6), 520-528. <https://doi.org/10.1097/dcr.0000000000000572> | Not parallel/cross-over RCT |
| 2009 | Other methods | Frost, J., Shaw, A., Montgomery, A., & Murphy, D. J. (2009). Women's views on the use of decision aids for decision making about the method of delivery following a previous caesarean section: qualitative interview study. *Bjog*, *116*(7), 896-905. <https://doi.org/10.1111/j.1471-0528.2009.02120.x> | Not parallel/cross-over RCT |
| 2015 | Other methods | LeBlanc, A., Wang, A. T., Wyatt, K., Branda, M. E., Shah, N. D., Van Houten, H., Pencille, L., Wermers, R., & Montori, V. M. (2015). Encounter Decision Aid vs. Clinical Decision Support or Usual Care to Support Patient-Centered Treatment Decisions in Osteoporosis: The Osteoporosis Choice Randomized Trial II. *PLoS One*, *10*(5), e0128063. <https://doi.org/10.1371/journal.pone.0128063> | Not elective surgery |
| 2011 | Other methods | Lurie, J. D., Spratt, K. F., Blood, E. A., Tosteson, T. D., Tosteson, A. N., & Weinstein, J. N. (2011). Effects of viewing an evidence-based video decision aid on patients' treatment preferences for spine surgery. *Spine (Phila Pa 1976)*, *36*(18), 1501-1504. <https://doi.org/10.1097/BRS.0b013e3182055c1e> | Secondary analysis of RCT |
| 2001 | Other methods | Molenaar, S., Sprangers, M. A., Rutgers, E. J. T., Luiten, E. J., Mulder, J., Bossuyt, P. M., Everdingen, J. J. v., Oosterveld, P., & Haes, H. C. d. (2001). Decision Support for Patients With Early-Stage Breast Cancer: Effects of an Interactive Breast Cancer CDROM on Treatment Decision, Satisfaction, and Quality of Life. *Journal of Clinical Oncology*, *19*(6), 1676-1687. <https://doi.org/10.1200/jco.2001.19.6.1676> | Not parallel/cross-over RCT |
| 2011 | Other methods | Montori, V. M., Shah, N. D., Pencille, L. J., Branda, M. E., Van Houten, H. K., Swiglo, B. A., Kesman, R. L., Tulledge-Scheitel, S. M., Jaeger, T. M., Johnson, R. E., Bartel, G. A., Melton, L. J., 3rd, & Wermers, R. A. (2011). Use of a decision aid to improve treatment decisions in osteoporosis: the osteoporosis choice randomized trial. *Am J Med*, *124*(6), 549-556. <https://doi.org/10.1016/j.amjmed.2011.01.013> | Not elective surgery |
| 2009 | Other methods | Raynes-Greenow, C. H., Roberts, C. L., Nassar, N., & Trevena, L. (2009). Do audio-guided decision aids improve outcomes? A randomized controlled trial of an audio-guided decision aid compared with a booklet decision aid for Australian women considering labour analgesia. *Health Expect*, *12*(4), 407-416. <https://doi.org/10.1111/j.1369-7625.2009.00553.x> | Not elective surgery |
| 2017 | Other methods | Sepucha, K., Atlas, S. J., Chang, Y., Dorrwachter, J., Freiberg, A., Mangla, M., Rubash, H. E., Simmons, L. H., & Cha, T. (2017). Patient Decision Aids Improve Decision Quality and Patient Experience and Reduce Surgical Rates in Routine Orthopaedic Care: A Prospective Cohort Study. *J Bone Joint Surg Am*, *99*(15), 1253-1260. <https://doi.org/10.2106/jbjs.16.01045> | Not parallel/cross-over RCT |
| 2009 | Databases and registers | Actrn. (2009). Evaluating an online decision aid for women considering breast reconstruction following mastectomy. [*http://www.who.int/trialsearch/Trial2.aspx?TrialID=ACTRN12609000363280*](http://www.who.int/trialsearch/Trial2.aspx?TrialID=ACTRN12609000363280). <https://www.cochranelibrary.com/central/doi/10.1002/central/CN-01837400/full> | No full-text available |
| 2017 | Databases and registers | Allen, L. A., McIlvennan, C. K., Thompson, J. S., Dunlay, S. M., LaRue, S. J., Lewis, E. F., Patel, C. B., Blue, L., Fairclough, D. L., Leister, E. C., & et al. (2017). Effectiveness of a shared decision making intervention for patients offered a destination therapy left ventricular assist device for end-stage heart failure: the DECIDE-LVAD trial [Journal: Conference Abstract]. *Circulation*, *136*, e461‐. <https://doi.org/10.1161/CIR.0000000000000546> | Conference abstract |
| 2005 | Databases and registers | Armstrong, K., Weber, B., Ubel, P. A., Peters, N., Holmes, J., & Schwartz, J. S. (2005). Individualized survival curves improve satisfaction with cancer risk management decisions in women with BRCA1/2 mutations [Journal: Article]. *Journal of Clinical Oncology*, *23*(36), 9319‐9328. <https://doi.org/10.1200/JCO.2005.06.119> | Not a decision aid |
| 2019 | Databases and registers | Bansback, N., Trenaman, L., MacDonald, K. V., Hawker, G., Johnson, J. A., Stacey, D., & Marshall, D. A. (2019). An individualized patient-reported outcome measure (PROM) based patient decision aid and surgeon report for patients considering total knee arthroplasty: protocol for a pragmatic randomized controlled trial. *BMC Musculoskelet Disord*, *20*(1), 89. <https://doi.org/10.1186/s12891-019-2434-2> | Study protocol |
| 1997 | Databases and registers | Barry, M. J., Cherkin, D. C., Chang, Y., Fowler, F. J., & Skates, S. (1997). A randomized trial of a multimedia shared decision-making program for men facing a treatment decision for benign prostatic hyperplasia [Journal: Article]. *Disease management and clinical outcomes*, *1*(1), 5‐14. <https://www.cochranelibrary.com/central/doi/10.1002/central/CN-00195062/full> | Conference abstract |
| 2019 | Databases and registers | Berger-Höger, B., Liethmann, K., Mühlhauser, I., Haastert, B., & Steckelberg, A. (2019). Nurse-led coaching of shared decision-making for women with ductal carcinoma in situ in breast care centers: A cluster randomized controlled trial. *International Journal of Nursing Studies*, *93*, 141-152. <https://doi.org/10.1016/j.ijnurstu.2019.01.013> | Not a decision aid |
| 2013 | Databases and registers | Berry, D. L., Halpenny, B., Hong, F., Wolpin, S., Lober, W. B., Russell, K. J., Ellis, W. J., Govindarajulu, U., Bosco, J., Davison, B. J., & et al. (2013). The Personal Patient Profile-Prostate decision support for men with localized prostate cancer: a multi-center randomized trial [Journal Article; Multicenter Study; Randomized Controlled Trial; Research Support, N.I.H., Extramural; Research Support, Non‐U.S. Gov't]. *Urologic oncology*, *31*(7), 1012‐1021. <https://doi.org/10.1016/j.urolonc.2011.10.004> | Same dataset as included study |
| 2012 | Databases and registers | Bosco, J. L. F., Halpenny, B., & Berry, D. L. (2012). Personal preferences and treatment choice in an intervention trial of men newly diagnosed with localized prostate cancer [Journal: Conference Abstract]. *Journal of Clinical Oncology*, *30*(5 SUPPL. 1). <https://www.cochranelibrary.com/central/doi/10.1002/central/CN-01028120/full> | Conference abstract |
| 2018 | Databases and registers | Boulware, L. E., Ephraim, P. L., Ameling, J., Lewis-Boyer, L., Rabb, H., Greer, R. C., Crews, D. C., Jaar, B. G., Auguste, P., Purnell, T. S., Lamprea-Monteleagre, J. A., Olufade, T., Gimenez, L., Cook, C., Campbell, T., Woodall, A., Ramamurthi, H., Davenport, C. A., Choudhury, K. R., & Weir, M. R. (2018). Effectiveness of informational decision aids and a live donor financial assistance program on pursuit of live kidney transplants in African American hemodialysis patients. *BMC Nephrology*, *19*(1), N.PAG-N.PAG. <https://doi.org/10.1186/s12882-018-0901-x> | No included outcomes |
| 2019 | Databases and registers | Brown, L., Gardner, G., & Bonner, A. (2019). A randomized controlled trial testing a decision support intervention for older patients with advanced kidney disease. *Journal of Advanced Nursing (John Wiley & Sons, Inc.)*, *75*(11), 3032-3044. <https://doi.org/10.1111/jan.14112> | Not elective surgery |
| 2015 | Databases and registers | Causarano, N., Platt, J., Baxter, N. N., Bagher, S., Jones, J. M., Metcalfe, K. A., Hofer, S. O., O'Neill, A. C., Cheng, T., Starenkyj, E., & et al. (2015). Pre-consultation educational group intervention to improve shared decision-making for postmastectomy breast reconstruction: a pilot randomized controlled trial [Journal Article; Randomized Controlled Trial; Research Support, Non‐U.S. Gov't]. *Supportive care in cancer*, *23*(5), 1365‐1375. <https://doi.org/10.1007/s00520-014-2479-6> | Not a decision aid |
| 2021 | Databases and registers | Chen, S. W., Yang, C. C., Te, J. C., Tsai, Y. L., Shorten, B., & Shorten, A. (2021). Birth choices after caesarean in Taiwan: a mixed methods pilot study of a decision aid for shared decision making [Journal: Article in Press]. *Midwifery*, *95*, 102920‐. <https://doi.org/10.1016/j.midw.2020.102920> | Not parallel/cross-over RCT |
| 2019 | Databases and registers | Cox, C. E., White, D. B., Hough, C. L., Jones, D. M., Kahn, J. M., Olsen, M. K., Lewis, C. L., Hanson, L. C., & Carson, S. S. (2019). Effects of a Personalized Web-Based Decision Aid for Surrogate Decision Makers of Patients With Prolonged Mechanical Ventilation: A Randomized Clinical Trial. *Annals of Internal Medicine*, *170*(5), 285-297. <https://doi.org/10.7326/M18-2335> | Surrogate as population |
| 2019 | Databases and registers | Doll, J. A., Jones, W. S., Lokhnygina, Y., Culpepper, S., Parks, R. L., Calhoun, C., Au, D. H., & Patel, M. R. (2019). PREPARED Study: A Study of Shared Decision-Making for Coronary Artery Disease. *Circ Cardiovasc Qual Outcomes*, *12*(2), e005244. <https://doi.org/10.1161/circoutcomes.118.005244> | Not parallel/cross-over RCT |
| 2016 | Databases and registers | Dowsey, M. M., Scott, A., Nelson, E. A., Li, J., Sundararajan, V., Nikpour, M., & Choong, P. F. (2016). Using discrete choice experiments as a decision aid in total knee arthroplasty: study protocol for a randomised controlled trial. *Trials*, *17*(1), 416. <https://doi.org/10.1186/s13063-016-1536-5> | Study protocol |
| 2018 | Databases and registers | Durand, M. A., Yen, R. W., O'Malley, A. J., Politi, M. C., Dhage, S., Rosenkranz, K., Weichman, K., Margenthaler, J., Tosteson, A. N. A., Crayton, E., Jackson, S., Bradley, A., Volk, R. J., Sepucha, K., Ozanne, E., Percac-Lima, S., Song, J., Acosta, J., Mir, N., & Elwyn, G. (2018). What matters most: protocol for a randomized controlled trial of breast cancer surgery encounter decision aids across socioeconomic strata. *BMC Public Health*, *18*(1), 241. <https://doi.org/10.1186/s12889-018-5109-2> | Study protocol |
| 2008 | Databases and registers | Emmett, C. L. (2008). *Decision aids for mode of delivery after previous caesarean section* (Publication Number U236497) [Ph.D., University of Bristol (United Kingdom)]. ProQuest Dissertations & Theses Global. Ann Arbor. | Same dataset as included study |
| 2015 | Databases and registers | Eslami, S., Aslani, A., Tara, F., Ghalichi, L., Erfanian, F., & Abu-Hanna, A. (2015). The impact of a computerized decision aid on empowering pregnant women for choosing vaginal versus cesarean section delivery: study protocol for a randomized controlled trial. *Trials*, *16*, 549. <https://doi.org/10.1186/s13063-015-1070-x> | Study protocol |
| 2018 | Databases and registers | Fletcher, J. W. A., Khan, M., & Thorpe, P. (2018). Study protocol for a randomised controlled trial of consenting processes and their effects on patient decision-making when undergoing spinal injections: the Risks In Spinal Consenting for Surgery (RISCS) trial. *BMJ Open*, *8*(9), e023426. <https://doi.org/10.1136/bmjopen-2018-023426> | Study protocol |
| 2021 | Databases and registers | Hadizadeh-Talasaz, F., Ghoreyshi, F., Mohammadzadeh, F., & Rahmani, R. (2021). Effect of shared decision making on mode of delivery and decisional conflict and regret in pregnant women with previous cesarean section: a randomized clinical trial [Journal: Article]. *BMC Pregnancy and Childbirth*, *21*(1). <https://doi.org/10.1186/s12884-021-03615-w> | Not a decision aid |
| 2020 | Databases and registers | Haji, S. Z. H., Firoozi, M., Asgharipour, N., & Shakeri, M. T. (2020). Impact of Motivational Interviewing on Women's Knowledge, Attitude and Intention to Choose Vaginal Birth after Caesarean Section: A Randomized Clinical Trial. *Journal of Midwifery & Reproductive Health*, *8*(1), 2114-2125. <https://doi.org/10.22038/jmrh.2019.40249.1451> | Not a decision aid |
| 2021 | Databases and registers | Hoffman, A. S., Hanson, S., Bordes, M. C., & Reece, G. (2021). Considering breast reconstruction after mastectomy: a randomized controlled trial testing a patient decision aid video and workbook [Meeting Abstract]. *Medical Decision Making*, *41*(4), E287-E288. [<Go to ISI>://WOS:000648637500220](http://gateway.isiknowledge.com/gateway/Gateway.cgi?GWVersion=2&SrcAuth=ResearchSoft&SrcApp=EndNote&DestLinkType=FullRecord&DestApp=WOS&KeyUT=000648637500220) | Conference abstract |
| 2010 | Databases and registers | Hollinghurst, S., Emmett, C., Peters, T. J., Watson, H., Fahey, T., Murphy, D. J., & Montgomery, A. (2010). Economic evaluation of the DiAMOND randomized trial: cost and outcomes of 2 decision aids for mode of delivery among women with a previous cesarean section [Journal Article; Randomized Controlled Trial; Research Support, Non‐U.S. Gov't]. *Medical Decision Making*, *30*(4), 453‐463. <https://doi.org/10.1177/0272989X09353195> | Same dataset as included study |
| 2011 | Databases and registers | Hooker, G. W., Leventhal, K. G., DeMarco, T., Peshkin, B. N., Finch, C., Wahl, E., Joines, J. R., Brown, K., Valdimarsdottir, H., & Schwartz, M. D. (2011). Longitudinal changes in patient distress following interactive decision aid use among BRCA1/2 carriers: a randomized trial. *Med Decis Making*, *31*(3), 412-421. <https://doi.org/10.1177/0272989x10381283> | No included outcomes |
| 2018 | Databases and registers | Huang, R., Zhang, D., Song, X., Xu, J., Hargraves, I., Montori, V., & Ting, H. H. (2018). Gaps between and within the knowledge of patients and cardiologists concerning the risks and benefits of percutaneous coronary intervention for chronic total occlusion: a randomized controlled trial of a decision aid [Journal: Conference Abstract]. *Journal of the American College of Cardiology*, *71*(11). <https://doi.org/10.1016/S0735-1097(18)33168-1> | Conference abstract |
| 2020 | Databases and registers | Joshi, S. P., Ramarajan, L., Deshpande, O., Fernandes, E., Vanmali, V., Hawaldar, R. W., Kannan, S., Parmar, V., Nair, N. S., Gupta, S., & et al. (2020). A randomized controlled trial of a self-administered, online decision-aid (“Navya Patient Preference Tool”) to reduce decisional conflict in women with early breast cancer [Journal: Conference Abstract]. *Journal of Clinical Oncology*, *38*(15). <https://doi.org/10.1200/JCO.2020.38.15-suppl.7033> | Conference abstract |
| 2021 | Databases and registers | Kharbanda, A. B., Vazquez-Benitez, G., Ballard, D. W., Vinson, D. R., Chettipally, U. K., Dehmer, S. P., Ekstrom, H., Rauchwerger, A. S., McMichael, B., Cotton, D. M., Kene, M. V., Simon, L. E., Zhu, J., Warton, E. M., O'Connor, P. J., & Kharbanda, E. O. (2021). Effect of Clinical Decision Support on Diagnostic Imaging for Pediatric Appendicitis: A Cluster Randomized Trial. *JAMA Network Open*, *4*(2), e2036344-e2036344. <https://doi.org/10.1001/jamanetworkopen.2020.36344> | Not elective surgery |
| 2009 | Databases and registers | Kuppermann, M., Norton, M. E., Gates, E., Gregorich, S. E., Learman, L. A., Nakagawa, S., Feldstein, V. A., Lewis, J., Washington, A. E., Nease, R. F., Jr., Kuppermann, M., Norton, M. E., Gates, E., Gregorich, S. E., Learman, L. A., Nakagawa, S., Feldstein, V. A., Lewis, J., Washington, A. E., & Nease, R. F., Jr. (2009). Computerized prenatal genetic testing decision-assisting tool: a randomized controlled trial. *Obstetrics & Gynecology*, *113*(1), 53-63. <https://doi.org/10.1097/AOG.0b013e31818e7ec4> | Not elective surgery |
| 2020 | Databases and registers | Matlock, D. D., McIlvennan, C. K., Thompson, J. S., Morris, M. A., Venechuk, G., Dunlay, S. M., LaRue, S. J., Lewis, E. F., Patel, C. B., Blue, L., & et al. (2020). Decision Aid Implementation among Left Ventricular Assist Device Programs Participating in the DECIDE-LVAD Stepped-Wedge Trial [Journal: Article]. *Medical Decision Making*, *40*(3), 289‐301. <https://doi.org/10.1177/0272989X20915227> | Programme evaluation |
| 2018 | Databases and registers | Matlock, D. D., Thompson, J., Morris, M. A., McIlvennan, C. K., Finnigan-Fox, G., Glasgow, R. E., Leister, E. C., Lewis, C., & Allen, L. (2018). Implementation of a shared decision support intervention for patients considering destination therapy left ventricular assist device: the decide-LVAD trial [Journal: Conference Abstract]. *Journal of the American College of Cardiology*, *71*(11). <https://doi.org/10.1016/S0735-1097(18)33188-7> | Conference abstract |
| 2019 | Databases and registers | Meier, J. D., Chorney, J. M., Fox, S. D., & Hong, P. (2019). Decision aid prototype for treatment of pediatric sleep disordered breathing: A randomized pilot study. *Laryngoscope*, *129*(1), 229-234. <https://doi.org/10.1002/lary.27204> | Surrogate as population |
| 2018 | Databases and registers | McIlvennan, C. K., Matlock, D. D., Thompson, J. S., Dunlay, S. M., Blue, L., LaRue, S. J., Lewis, E. F., Patel, C. B., Fairclough, D. L., Leister, E. C., Swetz, K. M., Baldridge, V., Walsh, M. N., & Allen, L. A. (2018). Caregivers of Patients Considering a Destination Therapy Left Ventricular Assist Device and a Shared Decision-Making Intervention: The DECIDE-LVAD Trial. *JACC Heart Fail*, *6*(11), 904-913. <https://doi.org/10.1016/j.jchf.2018.06.019> | Surrogate as population |
| 2019 | Databases and registers | Minneci, P. C., Cooper, J. N., Leonhart, K., Nacion, K., Sulkowski, J., Porter, K., Wei, L., & Deans, K. J. (2019). Effects of a Patient Activation Tool on Decision Making Between Surgery and Nonoperative Management for Pediatric Appendicitis: A Randomized Clinical Trial. *JAMA Network Open*, *2*(6), e195009-e195009. <https://doi.org/10.1001/jamanetworkopen.2019.5009> | Not a decision aid |
| 2020 | Databases and registers | Murphy, C., de Laine, C., Macaulay, M., & Fader, M. (2020). Development and randomised controlled trial of a Continence Product Patient Decision Aid for men post-radical prostatectomy [Journal: Article in Press]. *Journal of Clinical Nursing*. <https://doi.org/10.1111/jocn.15223> | Developmental paper |
| 2001 | Databases and registers | Murray, E., Davis, H., Tai, S. S., Coulter, A., Gray, A., & Haines, A. (2001). Randomised controlled trial of an interactive multimedia decision aid on benign prostatic hypertrophy in primary care. *Bmj*, *323*(7311), 493-496. <https://doi.org/10.1136/bmj.323.7311.493> | Setting in primary care |
| 2020 | Databases and registers | Politi, M., Lee, C., Philpott-Streiff, S., Foraker, R., Olsen, M., Merrill, C., Tao, Y., & Myckatyn, T. (2020). Incorporating personalized risk prediction into clinical decision support: a randomized controlled trial for post-mastectomy breast reconstruction [Meeting Abstract]. *Medical Decision Making*, *40*(1), E14-E15. [<Go to ISI>://WOS:000509275600022](http://gateway.isiknowledge.com/gateway/Gateway.cgi?GWVersion=2&SrcAuth=ResearchSoft&SrcApp=EndNote&DestLinkType=FullRecord&DestApp=WOS&KeyUT=000509275600022) | Conference abstract |
| 2011 | Databases and registers | Rustveld, L., & Jibaja-Weiss, M. (2011). A patchwork of life: a bilingual breast cancer treatment patient decision aid targeted at medically underserved women [Journal: Conference Abstract]. *Cancer epidemiology biomarkers and prevention*, *20*(10). <https://doi.org/10.1158/1055-9965.DISP-11-A31> | Conference abstract |
| 2012 | Databases and registers | Schwalm, J. D., Stacey, D., Pericak, D., & Natarajan, M. K. (2012). Radial artery versus femoral artery access options in coronary angiogram procedures: randomized controlled trial of a patient-decision aid. *Circulation: Cardiovascular Quality & Outcomes*, *5*(3), 260-266. <http://libproxy1.nus.edu.sg/login?url=https://search.ebscohost.com/login.aspx?direct=true&db=rzh&AN=108114367&site=ehost-live> | Not elective surgery |
| 2019 | Databases and registers | Sepucha, K., Bedair, H., Yu, L., Dorrwachter, J. M., Dwyer, M., Talmo, C. T., Vo, H., & Freiberg, A. A. (2019). Decision Support Strategies for Hip and Knee Osteoarthritis: Less Is More: A Randomized Comparative Effectiveness Trial (DECIDE-OA Study). *J Bone Joint Surg Am*, *101*(18), 1645-1653. <https://doi.org/10.2106/jbjs.19.00004> | Not parallel/cross-over RCT |
| 2012 | Databases and registers | Sherman, K. A., Harcourt, D., Lam, T., & Boyages, J. (2012). Breconda: development and acceptability of an interactive decisional support tool for women considering breast reconstruction [Journal: Conference Abstract]. *Cancer research*, *72*(24 SUPPL. 3). <https://doi.org/10.1158/0008-5472.SABCS12-P4-17-04> | Conference abstract |
| 2013 | Databases and registers | Sherman, K., Harcourt, D., Lam, T., Boyages, J., Brown, P., Cameron, L., Shaw, L. K., & Winch, C. (2013). Facilitating decision making of women considering breast reconstruction following mastectomy: results from a randomised controlled trial of the breconda web-based decision aid [Journal: Conference Abstract]. *Psycho-Oncology*, *22*, 22‐23. <https://doi.org/10.1111/j.1099-1611.2013.3393> | Conference abstract |
| 2017 | Databases and registers | Sherman, K. A., Kilby, C. J., Shaw, L. K., Winch, C., Kirk, J., Tucker, K., & Elder, E. (2017). Facilitating decision-making in women undergoing genetic testing for hereditary breast cancer: BRECONDA randomized controlled trial results. *Breast*, *36*, 79-85. <https://doi.org/10.1016/j.breast.2017.10.001> | Same dataset as included study |
| 2014 | Databases and registers | Stacey, D., Hawker, G., Dervin, G., Tugwell, P., Boland, L., Pomey, M. P., O'Connor, A. M., & Taljaard, M. (2014). Decision aid for patients considering total knee arthroplasty with preference report for surgeons: a pilot randomized controlled trial. *BMC Musculoskelet Disord*, *15*, 54. <https://doi.org/10.1186/1471-2474-15-54> | Pilot study of same dataset |
| 2019 | Databases and registers | Ter Stege, J. A., Woerdeman, L. A. E., Hahn, D. E. E., van Huizum, M. A., van Duijnhoven, F. H., Kieffer, J. M., Retèl, V. P., Sherman, K. A., Witkamp, A. J., Oldenburg, H. S. A., & Bleiker, E. M. A. (2019). The impact of an online patient decision aid for women with breast cancer considering immediate breast reconstruction: study protocol of a multicenter randomized controlled trial. *BMC Med Inform Decis Mak*, *19*(1), 165. <https://doi.org/10.1186/s12911-019-0873-1> | Study protocol |
| 2016 | Databases and registers | Tol‐Geerdink, J. J., Leer, J. W. H., Wijburg, C. J., Oort, I. M., Vergunst, H., Lin, E. J., Witjes, J. A., & Stalmeier, P. F. M. (2016). Does a decision aid for prostate cancer affect different aspects of decisional regret, assessed with new regret scales? A randomized, controlled trial [Academic Journal]. *Health Expectations*, *19*(2), 459‐470. <https://doi.org/10.1111/hex.12369> | Psychometric properties testing |
| 2017 | Databases and registers | Tucholka, J., Yang, D. Y., Bruce, J., Schumacher, J., Steiman, J., Wilke, L., Greenberg, C., & Neuman, H. (2017). Impact of the receipt of pre-consultation web-based material on patients' value-concordant decision-making for type of breast cancer surgery [Journal: Conference Abstract]. *Annals of Surgical Oncology*, *24*(2), 150‐151. <https://doi.org/10.1245/s10434-017-5854-y> | Conference abstract |
| 2011 | Databases and registers | Vodermaier, A., Caspari, C., Wang, L., Koehm, J., Ditsch, N., & Untch, M. (2011). How and For Whom Are Decision Aids Effective? Long-Term Psychological Outcome of a Randomized Controlled Trial in Women With Newly Diagnosed Breast Cancer. *Health psychology*, *30*(1), 12-19. <https://doi.org/10.1037/a0021648> | Same dataset as included study |
| 2015 | Databases and registers | Warner, D. O., LeBlanc, A., Kadimpati, S., Vickers, K. S., Shi, Y., & Montori, V. M. (2015). Decision Aid for Cigarette Smokers Scheduled for Elective Surgery. *Anesthesiology*, *123*(1), 18-28. <https://doi.org/10.1097/aln.0000000000000704> | No surgical candidate |
| 2018 | Databases and registers | Waterman, A. D., & Peipert, J. D. (2018). An Explore Transplant Group Randomized Controlled Education Trial to Increase Dialysis Patients' Decision-Making and Pursuit of Transplantation. *Progress in Transplantation*, *28*(2), 174-183. <https://doi.org/10.1177/1526924818765815> | Not a decision aid |
| 2003 | Databases and registers | Whelan, T., Sawka, C., Levine, M., Gafni, A., Reyno, L., Willan, A., Julian, J., Dent, S., Abu-Zahra, H., Chouinard, E., & et al. (2003). Helping patients make informed choices: a randomized trial of a decision aid for adjuvant chemotherapy in lymph node-negative breast cancer [Clinical Trial; Journal Article; Randomized Controlled Trial; Research Support, Non‐U.S. Gov't]. *Journal of the National Cancer Institute*, *95*(8), 581‐587. <https://doi.org/10.1093/jnci/95.8.581> | Not elective surgery |

# **Supplementary Table 6** Outcomes and their respective definition and measures.

| **Outcomes** | **Definition** | **Measures** |
| --- | --- | --- |
| **Invasive treatment choice** | |  |
| Invasive treatment choice | A treatment on choices based upon a set of criteria determined by clinician and/or patient, on whether to proceed with invasive surgery. However, when studies did not report the actual choice, we used the patients' preferred option as a surrogate measure. | - |
| **Decision-making related outcomes** | | |
| Decisional conflict | Personal uncertainty about which course of action to take when choosing between competing medical/surgical options entails risk, regret, or a challenge to personal life values. | DCS (16 items, 10 items, and SURE tool version for clinical practice) |
| Satisfaction with decision-making | Contentment with a choice that results from a complex analysis and consideration of multiple factors. | Effective decision subscale of DCS, patient satisfaction survey with treatment/surgery, visit/consultation, decision, decision-making process, and information provided |
| Disease and treatment knowledge | A collection of beliefs based on the information the patient has gathered about various aspects of the disease and therapy options over the course of his/her life, both before and after the diagnosis/surgical consultation. | K-DQI/DQI knowledge subscale, BCIT-R, self-developed knowledge questionnaire about the disease and elective surgery |
| Decisional regret | Distress or remorse after a surgery among patients who chose to have surgical treatment. | DRS |
| Preparedness for decision-making | The patient's assessment of the effectiveness of a decision aid or other decision support intervention in preparing the respondent to interact with their practitioner during a consultation focused on making a health decision. | RPAD, PDM |
| Decision quality | The reasonableness and significance of a decision at the time it is made, regardless of its outcome. | K-DQI/DQI |
| Value concordance | Capability of patients to achieve concordance between their values, preferences, and the actual treatment that is carried out. | K-DQI/DQI |
| Shared decision-making | A process by which clinicians and patients make decisions together using the best available evidence about the likely benefits and harms of each option, and where patients are supported to arrive at informed preferences. | CollaboRATE,, SDM-Q-9 |
| Decision self-efficacy | Self-confidence or belief in one's ability to make decisions, including participate in shared decision making. | DSES, SEMCD-6 |
| Outcome expectations | Successful correspondence to known evidence of the probability of outcomes for a person’s health profile. | - |
| **Patient-reported outcomes** | |  |
| General HRQoL | Measure of how an individual perceives his or her health. | SF-36 (General health), EQ VAS, self-developed 11-point general health scale |
| Condition-specific HRQoL | Specific measures of health status and quality of life to different diseases, conditions, states, and populations. | KOOS JR, BREAST-Q, QuickDASH |
| Physical health | The ability to perform daily tasks and live comfortably in one’s body. | PROMIS PF, SF-36 (PCS) |
| Mental health | A person’s condition with regard to their psychological and emotional well-being. | Chinese Health Questionnaire, SF-36 (MCS) |
| Depression | Depression is a mood disorder that causes a persistent feeling of sadness and loss of interest. | HADS – depression, CES-D, PHQ-2 |
| Anxiety | Anxiety is an emotion characterized by feelings of tension, worried thoughts and physical changes like increased blood pressure. | HADS – Anxiety, STAI |
| Perceived stress | Individual's feelings or thoughts about the amount of stress they are experiencing at any given time or over a specified time period. | PSS, IES, Cancer-related distress |
| **Healthcare resource utilization** | |  |
| Consultation time | The time taken from completed assessment to consultation by the clinician or specialist in the consultation room. | - |
| BCIT-R: Breast Cancer Information Test–Revised; CES-D: Center for Epidemiologic Studies Depression Scale; DCS: Decision Conflict Scale; DRS: Decision Regret Scale; DSES: Decision Self Efficacy Scale; HADS: Hospital Anxiety and Depression Scale; IES: Impact of Event Scale; K-DQI: Knee Osteoarthritis Decision Quality Instrument; KOOS JR: Knee Injury and Osteoarthritis Outcome Score, Joint Replacement; MCS: Mental Component Summary; PCS: Physical Component Summary; PDM: Preparedness for decision making; PHQ: Patient Health Questionnaire; PROMIS: Patient-Reported Outcomes Measurement Information System; PSS: Perceived Stress Scale; QuickDASH: shortened version of the DASH Outcome Measure; RPAD: Rochester Participatory Decision-Making Scale; SDM-Q-9: 9-item Shared Decision Making Questionnaire; SEMCD-6: Self-Efficacy for Managing Chronic Diseases 6-item; Scale STAI: State-Trait Anxiety Inventory | | |

# **Supplementary Table 7** Study code and references for all included studies.

| **Code** | **References of included studies** |
| --- | --- |
| A001 | Allen, K. D., Sanders, L. L., Olsen, M. K., Bowlby, L., Katz, J. N., Mather, R. C., 3rd, & Williams, J. W., Jr. (2016). Internet Versus DVD Decision Aids for Hip and Knee Osteoarthritis. Musculoskeletal Care, 14(2), 87-97. <https://doi.org/10.1002/msc.1116> |
| A002 | Allen, L. A., McIlvennan, C. K., Thompson, J. S., Dunlay, S. M., LaRue, S. J., Lewis, E. F., Patel, C. B., Blue, L., Fairclough, D. L., Leister, E. C., Glasgow, R. E., Cleveland, J. C., Jr., Phillips, C., Baldridge, V., Walsh, M. N., & Matlock, D. D. (2018). Effectiveness of an Intervention Supporting Shared Decision Making for Destination Therapy Left Ventricular Assist Device: The DECIDE-LVAD Randomized Clinical Trial. JAMA Intern Med, 178(4), 520-529. <https://doi.org/10.1001/jamainternmed.2017.8713> |
| A003 | Arterburn, D. E., Westbrook, E. O., Bogart, T. A., Sepucha, K. R., Bock, S. N., & Weppner, W. G. (2011). Randomized trial of a video-based patient decision aid for bariatric surgery. Obesity (Silver Spring), 19(8), 1669-1675. <https://doi.org/10.1038/oby.2011.65> |
| A004 | Auvinen, A., Hakama, M., Ala-Opas, M., Vornanen, T., Leppilahti, M., Salminen, P., & Tammela, T. L. (2004). A randomized trial of choice of treatment in prostate cancer: the effect of intervention on the treatment chosen. BJU Int, 93(1), 52-56; discussion 56. <https://doi.org/10.1111/j.1464-410x.2004.04554.x> |
| A005 | Berry, D. L., Hong, F., Blonquist, T. M., Halpenny, B., Filson, C. P., Master, V. A., Sanda, M. G., Chang, P., Chien, G. W., Jones, R. A., Krupski, T. L., Wolpin, S., Wilson, L., Hayes, J. H., Trinh, Q. D., Sokoloff, M., & Somayaji, P. (2018). Decision Support with the Personal Patient Profile-Prostate: A Multicenter Randomized Trial. J Urol, 199(1), 89-97. <https://doi.org/10.1016/j.juro.2017.07.076> |
| A006 | Bozic, K. J., Belkora, J., Chan, V., Youm, J., Zhou, T., Dupaix, J., Bye, A. N., Braddock, C. H., 3rd, Chenok, K. E., & Huddleston, J. I., 3rd. (2013). Shared decision making in patients with osteoarthritis of the hip and knee: results of a randomized controlled trial. J Bone Joint Surg Am, 95(18), 1633-1639. <https://doi.org/10.2106/jbjs.M.00004> |
| A007 | Coylewright, M., Dick, S., Zmolek, B., Askelin, J., Hawkins, E., Branda, M., Inselman, J. W., Zeballos-Palacios, C., Shah, N. D., Hess, E. P., LeBlanc, A., Montori, V. M., & Ting, H. H. (2016). PCI Choice Decision Aid for Stable Coronary Artery Disease: A Randomized Trial. Circ Cardiovasc Qual Outcomes, 9(6), 767-776. [https://doi.org/10.1161/circoutcomes.116.002641](https://doi.org/10.1161/circoutcomes.116.002641%20) |
| A008 | de Achaval, S., Fraenkel, L., Volk, R. J., Cox, V., & Suarez-Almazor, M. E. (2012). Impact of educational and patient decision aids on decisional conflict associated with total knee arthroplasty. Arthritis Care Res (Hoboken), 64(2), 229-237. [https://doi.org/10.1002/acr.20646](https://doi.org/10.1002/acr.20646%20) |
| A009 | Deyo, R. A., Cherkin, D. C., Weinstein, J., Howe, J., Ciol, M., & Mulley, A. G., Jr. (2000). Involving patients in clinical decisions: impact of an interactive video program on use of back surgery. Med Care, 38(9), 959-969. [https://doi.org/10.1097/00005650-200009000-00009](https://doi.org/10.1097/00005650-200009000-00009%20) |
| A010 | Eden, K. B., Perrin, N. A., Vesco, K. K., & Guise, J. M. (2014). A randomized comparative trial of two decision tools for pregnant women with prior cesareans [Comparative Study; Journal Article; Randomized Controlled Trial; Research Support, N.I.H., Extramural; Research Support, Non‐U.S. Gov't; Research Support, U.S. Gov't, P.H.S.]. Journal of obstetric, gynecologic, and neonatal nursing : JOGNN, 43(5), 568‐579. [https://doi.org/10.1111/1552-6909.12485](https://doi.org/10.1111/1552-6909.12485%20) |
| A011 | Goel, V., Sawka, C. A., Thiel, E. C., Gort, E. H., & O'Connor, A. M. (2001). Randomized trial of a patient decision aid for choice of surgical treatment for breast cancer. Med Decis Making, 21(1), 1-6. [https://doi.org/10.1177/0272989x0102100101](https://doi.org/10.1177/0272989x0102100101%20) |
| A012 | Gokce, M., Akpınar, C., Esen, B., Solak, V., Gülpınar, O., & Bedük, Y. (2019). The role of a novel decision aid to support informed decision making process in patients with a symptomatic non - lower pole renal stone < 20 mm in diameter: a prospective randomized study. Int Braz J Urol, 45(5), 941-947. [https://doi.org/10.1590/s1677-5538.Ibju.2018.0198](https://doi.org/10.1590/s1677-5538.Ibju.2018.0198%20) |
| A013 | Hawley, S. T., Li, Y., An, L. C., Resnicow, K., Janz, N. K., Sabel, M. S., Ward, K. C., Fagerlin, A., Morrow, M., Jagsi, R., & et al. (2018). Improving breast cancer surgical treatment decision making: the iCanDecide randomized clinical trial [Journal: Article]. Journal of Clinical Oncology, 36(7), 659‐666. [https://doi.org/10.1200/JCO.2017.74.8442](https://doi.org/10.1200/JCO.2017.74.8442%20) |
| A014 | Heller, L., Parker, P. A., Youssef, A., & Miller, M. J. (2008). Interactive digital education aid in breast reconstruction. Plast Reconstr Surg, 122(3), 717-724. [https://doi.org/10.1097/PRS.0b013e318180ed06](https://doi.org/10.1097/PRS.0b013e318180ed06%20) |
| A015 | Hutyra, C. A., Smiley, S., Taylor, D. C., Orlando, L. A., & Mather Iii, R. C. (2019). Efficacy of a Preference-Based Decision Tool on Treatment Decisions for a First-Time Anterior Shoulder Dislocation: A Randomized Controlled Trial of At-Risk Patients. Medical Decision Making, 39(3), 253-263. [https://doi.org/10.1177/0272989X19832915](https://doi.org/10.1177/0272989X19832915%20) |
| A016 | Ibrahim, S. A., Blum, M., Lee, G. C., Mooar, P., Medvedeva, E., Collier, A., & Richardson, D. (2017). Effect of a Decision Aid on Access to Total Knee Replacement for Black Patients With Osteoarthritis of the Knee: A Randomized Clinical Trial. JAMA Surg, 152(1), e164225. [https://doi.org/10.1001/jamasurg.2016.4225](https://doi.org/10.1001/jamasurg.2016.4225%20) |
| A017 | Jayakumar, P., Moore, M. G., Furlough, K. A., Uhler, L. M., Andrawis, J. P., Koenig, K. M., Aksan, N., Rathouz, P. J., & Bozic, K. J. (2021). Comparison of an Artificial Intelligence-Enabled Patient Decision Aid vs Educational Material on Decision Quality, Shared Decision-Making, Patient Experience, and Functional Outcomes in Adults With Knee Osteoarthritis: A Randomized Clinical Trial. JAMA Netw Open, 4(2), e2037107. [https://doi.org/10.1001/jamanetworkopen.2020.37107](https://doi.org/10.1001/jamanetworkopen.2020.37107%20) |
| A018 | Jibaja-Weiss, M. L., Volk, R. J., Granchi, T. S., Neff, N. E., Robinson, E. K., Spann, S. J., Aoki, N., Friedman, L. C., & Beck, J. R. (2011). Entertainment education for breast cancer surgery decisions: a randomized trial among patients with low health literacy. Patient Educ Couns, 84(1), 41-48. [https://doi.org/10.1016/j.pec.2010.06.009](https://doi.org/10.1016/j.pec.2010.06.009%20) |
| A019 | Kearing, S., Berg, S. Z., & Lurie, J. D. (2016). Can Decision Support Help Patients With Spinal Stenosis Make a Treatment Choice?: A Prospective Study Assessing the Impact of a Patient Decision Aid and Health Coaching. Spine, 41(7). [https://doi.org/10.1097/BRS.0000000000001272](https://doi.org/10.1097/BRS.0000000000001272%20) |
| A020 | Kennedy, A. D., Sculpher, M. J., Coulter, A., Dwyer, N., Rees, M., Abrams, K. R., Horsley, S., Cowley, D., Kidson, C., Kirwin, C., Naish, C., & Stirrat, G. (2002). Effects of decision aids for menorrhagia on treatment choices, health outcomes, and costs: a randomized controlled trial. Jama, 288(21), 2701-2708. [https://doi.org/10.1001/jama.288.21.2701](https://doi.org/10.1001/jama.288.21.2701%20) |
| A021 | Kleiss, I. I. M., Kortlever, J. T. P., Ring, D., Vagner, G. A., & Reichel, L. M. (2021). A Randomized Controlled Trial of Decision Aids for Upper-Extremity Conditions. J Hand Surg Am, 46(4), 338.e331-338.e315. [https://doi.org/10.1016/j.jhsa.2020.09.003](https://doi.org/10.1016/j.jhsa.2020.09.003%20) |
| A022 | Korteland, N. M., Ahmed, Y., Koolbergen, D. R., Brouwer, M., De Heer, F., Kluin, J., Bruggemans, E. F., Klautz, R. J. M., Stiggelbout, A. M., Bucx, J. J. J., Roos-Hesselink, J. W., Polak, P., Markou, T., Van Den Broek, I., Ligthart, R., Bogers, A. J. J. C., & Takkenberg, J. J. M. (2017). Does the use of a decision aid improve decision making in prosthetic heart valve selection? [Article]. Circulation: cardiovascular quality and outcomes, 10(2). [https://doi.org/10.1161/CIRCOUTCOMES.116.003178](https://doi.org/10.1161/CIRCOUTCOMES.116.003178%20) |
| A023 | Kostick, K. M., Bruce, C. R., Minard, C. G., Volk, R. J., Civitello, A., Krim, S. R., Horstmanshof, D., Thohan, V., Loebe, M., Hanna, M., Bruckner, B. A., Blumenthal Barby, J. S., & Estep, J. D. (2018). A Multisite Randomized Controlled Trial of a Patient-Centered Ventricular Assist Device Decision Aid (VADDA Trial). J Card Fail, 24(10), 661-671. [https://doi.org/10.1016/j.cardfail.2018.08.008](https://doi.org/10.1016/j.cardfail.2018.08.008%20) |
| A024 | Kuppermann, M., Kaimal, A. J., Blat, C., Gonzalez, J., Thiet, M. P., Bermingham, Y., Altshuler, A. L., Bryant, A. S., Bacchetti, P., & Grobman, W. A. (2020). Effect of a Patient-Centered Decision Support Tool on Rates of Trial of Labor After Previous Cesarean Delivery: the PROCEED Randomized Clinical Trial [Comparative Study; Journal Article; Multicenter Study; Randomized Controlled Trial; Research Support, N.I.H., Extramural]. Jama, 323(21), 2151‐2159. [https://doi.org/10.1001/jama.2020.5952](https://doi.org/10.1001/jama.2020.5952%20) |
| A025 | Lam, W. W., Fielding, R., Butow, P., Cowling, B. J., Chan, M., Or, A., Kwong, A., & Suen, D. (2014). Decision aids for breast cancer surgery: a randomised controlled trial [Journal Article; Randomized Controlled Trial; Research Support, Non‐U.S. Gov't]. Hong kong medical journal = xianggang yi xue za zhi, 20 Suppl 7, 24‐27. [https://www.cochranelibrary.com/central/doi/10.1002/central/CN-01110724/full](https://www.cochranelibrary.com/central/doi/10.1002/central/CN-01110724/full%20) |
| A026 | Lamers, R. E. D., Cuypers, M., de Vries, M., van de Poll-Franse, L. V., Bosch, J. L. H. R., & Kil, P. J. M. (2021). Differences in treatment choices between prostate cancer patients using a decision aid and patients receiving care as usual: results from a randomized controlled trial [Article in Press]. World journal of urology. [https://doi.org/10.1007/s00345-021-03782-7](https://doi.org/10.1007/s00345-021-03782-7%20) |
| A027 | Luan, A., Hui, K. J., Remington, A. C., Liu, X., & Lee, G. K. (2016). Effects of A Novel Decision Aid for Breast Reconstruction: A Randomized Prospective Trial. Ann Plast Surg, 76 Suppl 3, S249-254. [https://doi.org/10.1097/sap.0000000000000722](https://doi.org/10.1097/sap.0000000000000722%20) |
| A028 | Manne, S. L., Topham, N., D'Agostino, T. A., Myers Virtue, S., Kirstein, L., Brill, K., Manning, C., Grana, G., Schwartz, M. D., & Ohman-Strickland, P. (2016). Acceptability and pilot efficacy trial of a web-based breast reconstruction decision support aid for women considering mastectomy. Psychooncology, 25(12), 1424-1433. <https://doi.org/10.1002/pon.3984> |
| A029 | Manne, S. L., Smith, B. L., Frederick, S., Mitarotondo, A., Kashy, D. A., & Kirstein, L. J. (2020). B-Sure: a randomized pilot trial of an interactive web-based decision support aid versus usual care in average-risk breast cancer patients considering contralateral prophylactic mastectomy. Transl Behav Med, 10(2), 355-363. <https://doi.org/10.1093/tbm/iby133> |
| A030 | Metcalfe, K. A., Dennis, C. L., Poll, A., Armel, S., Demsky, R., Carlsson, L., Nanda, S., Kiss, A., & Narod, S. A. (2017). Effect of decision aid for breast cancer prevention on decisional conflict in women with a BRCA1 or BRCA2 mutation: a multisite, randomized, controlled trial [Journal Article; Multicenter Study; Randomized Controlled Trial]. Genetics in medicine, 19(3), 330‐336. [https://doi.org/10.1038/gim.2016.108](https://doi.org/10.1038/gim.2016.108%20) |
| A031 | Montgomery, A. A., Emmett, C. L., Fahey, T., Jones, C., Ricketts, I., Patel, R. R., Peters, T. J., & Murphy, D. J. (2007). Two decision aids for mode of delivery among women with previous caesarean section: randomised controlled trial. Bmj, 334(7607), 1305. [https://doi.org/10.1136/bmj.39217.671019.55](https://doi.org/10.1136/bmj.39217.671019.55%20) |
| A032 | Parkinson, B., Sherman, K. A., Brown, P., Shaw, L. E., Boyages, J., Cameron, L. D., Elder, E., & Lam, T. (2018). Cost-effectiveness of the BRECONDA decision aid for women with breast cancer: Results from a randomized controlled trial. Psychooncology, 27(6), 1589-1596. [https://doi.org/10.1002/pon.4698](https://doi.org/10.1002/pon.4698%20) |
| A033 | Phelan, E. A., Deyo, R. A., Cherkin, D. C., Weinstein, J. N., Ciol, M. A., Kreuter, W., & Howe, J. F. (2001). Helping patients decide about back surgery: a randomized trial of an interactive video program. Spine (Phila Pa 1976), 26(2), 206-211;discussion 212. [https://doi.org/10.1097/00007632-200101150-00016](https://doi.org/10.1097/00007632-200101150-00016%20) |
| A034 | Politi, M. C., Lee, C. N., Philpott-Streiff, S. E., Foraker, R. E., Olsen, M. A., Merrill, C., Tao, Y., & Myckatyn, T. M. (2020). A Randomized Controlled Trial Evaluating the BREASTChoice Tool for Personalized Decision Support About Breast Reconstruction After Mastectomy. Ann Surg, 271(2), 230-237. [https://doi.org/10.1097/sla.0000000000003444](https://doi.org/10.1097/sla.0000000000003444%20) |
| A035 | Rivero-Santana, A., Torrente-Jiménez, R. S., Perestelo-Pérez, L., Torres-Castaño, A., Ramos-García, V., Bilbao, A., Escobar, A., Serrano-Aguilar, P., & Feijoo-Cid, M. (2021). Effectiveness of a decision aid for patients with knee osteoarthritis: a randomized controlled trial [Article]. Osteoarthritis and cartilage, 29(9), 1265-1274. [https://doi.org/10.1016/j.joca.2021.06.005](https://doi.org/10.1016/j.joca.2021.06.005%20) |
| A036 | Schwartz, M. D., Valdimarsdottir, H. B., DeMarco, T. A., Peshkin, B. N., Lawrence, W., Rispoli, J., Brown, K., Isaacs, C., O'Neill, S., Shelby, R., Grumet, S. C., McGovern, M. M., Garnett, S., Bremer, H., Leaman, S., O'Mara, K., Kelleher, S., & Komaridis, K. (2009). Randomized trial of a decision aid for BRCA1/BRCA2 mutation carriers: impact on measures of decision making and satisfaction. Health Psychol, 28(1), 11-19. [https://doi.org/10.1037/a0013147](https://doi.org/10.1037/a0013147%20) |
| A037 | Sherman, K. A., Shaw, L. E., Winch, C. J., Harcourt, D., Boyages, J., Cameron, L. D., Brown, P., Lam, T., Elder, E., French, J., & Spillane, A. (2016). Reducing Decisional Conflict and Enhancing Satisfaction with Information among Women Considering Breast Reconstruction following Mastectomy: Results from the BRECONDA Randomized Controlled Trial. Plast Reconstr Surg, 138(4), 592e-602e. [https://doi.org/10.1097/prs.0000000000002538](https://doi.org/10.1097/prs.0000000000002538%20) |
| A038 | Shorten, A., Shorten, B., Keogh, J., West, S., & Morris, J. (2005). Making choices for childbirth: a randomized controlled trial of a decision-aid for informed birth after cesarean [Journal Article; Multicenter Study; Randomized Controlled Trial; Research Support, Non‐U.S. Gov't]. Birth (Berkeley, Calif.), 32(4), 252‐261. [https://doi.org/10.1111/j.0730-7659.2005.00383.x](https://doi.org/10.1111/j.0730-7659.2005.00383.x%20) |
| A039 | Shue, J., Karia, R. J., Cardone, D., Samuels, J., Shah, M., & Slover, J. D. (2016). A Randomized Controlled Trial of Two Distinct Shared Decision-Making Aids for Hip and Knee Osteoarthritis in an Ethnically Diverse Patient Population. Value Health, 19(4), 487-493. [https://doi.org/10.1016/j.jval.2016.01.006](https://doi.org/10.1016/j.jval.2016.01.006%20) |
| A040 | Stacey, D., Taljaard, M., Dervin, G., Tugwell, P., O'Connor, A. M., Pomey, M. P., Boland, L., Beach, S., Meltzer, D., & Hawker, G. (2016). Impact of patient decision aids on appropriate and timely access to hip or knee arthroplasty for osteoarthritis: a randomized controlled trial. Osteoarthritis Cartilage, 24(1), 99-107. [https://doi.org/10.1016/j.joca.2015.07.024](https://doi.org/10.1016/j.joca.2015.07.024%20) |
| A041 | Stiggelbout, A. M., Molewijk, A. C., Otten, W., Van Bockel, J. H., Bruijninckx, C. M., Van der Salm, I., & Kievit, J. (2008). The impact of individualized evidence-based decision support on aneurysm patients' decision making, ideals of autonomy, and quality of life. Med Decis Making, 28(5), 751-762. [https://doi.org/10.1177/0272989x08321680](https://doi.org/10.1177/0272989x08321680%20) |
| A042 | Street, R. L., Jr., Voigt, B., Geyer, C., Jr., Manning, T., & Swanson, G. P. (1995). Increasing patient involvement in choosing treatment for early breast cancer. Cancer, 76(11), 2275-2285. https://doi.org/10.1002/1097-0142(19951201)76:11<2275::aid-cncr2820761115>3.0.co;2-s |
| A043 | Trenaman, L., Stacey, D., Bryan, S., Taljaard, M., Hawker, G., Dervin, G., Tugwell, P., & Bansback, N. (2017). Decision aids for patients considering total joint replacement: a cost-effectiveness analysis alongside a randomised controlled trial. Osteoarthritis Cartilage, 25(10), 1615-1622. [https://doi.org/10.1016/j.joca.2017.05.022](https://doi.org/10.1016/j.joca.2017.05.022%20) |
| A044 | Trenaman, L., Stacey, D., Bryan, S., Payne, K., Hawker, G., & Bansback, N. (2020). Long-term effect of patient decision aids on use of joint replacement and health care costs. Osteoarthritis Cartilage, 28(6), 819-823. <https://doi.org/10.1016/j.joca.2020.01.019> |
| A045 | Tucholka, J. L., Yang, D.-Y., Bruce, J. G., Steffens, N. M., Schumacher, J. R., Greenberg, C. C., Wilke, L. G., Steiman, J., & Neuman, H. B. (2018). A Randomized Controlled Trial Evaluating the Impact of Web-Based Information on Breast Cancer Patients' Knowledge of Surgical Treatment Options. Journal of the American College of Surgeons, 226(2), 126-133. [https://doi.org/10.1016/j.jamcollsurg.2017.10.024](https://doi.org/10.1016/j.jamcollsurg.2017.10.024%20) |
| A046 | van Roosmalen, M. S., Stalmeier, P. F., Verhoef, L. C., Hoekstra-Weebers, J. E., Oosterwijk, J. C., Hoogerbrugge, N., Moog, U., & van Daal, W. A. (2004a). Randomised trial of a decision aid and its timing for women being tested for a BRCA1/2 mutation [Clinical Trial; Journal Article; Randomized Controlled Trial]. British Journal of Cancer, 90(2), 333‐342. [https://doi.org/10.1038/sj.bjc.6601525](https://doi.org/10.1038/sj.bjc.6601525%20) |
| A047 | van Roosmalen, M. S., Stalmeier, P. F., Verhoef, L. C., Hoekstra-Weebers, J. E., Oosterwijk, J. C., Hoogerbrugge, N., Moog, U., & van Daal, W. A. (2004b). Randomized trial of a shared decision-making intervention consisting of trade-offs and individualized treatment information for BRCA1/2 mutation carriers. J Clin Oncol, 22(16), 3293-3301. [https://doi.org/10.1200/jco.2004.05.066](https://doi.org/10.1200/jco.2004.05.066%20) |
| A048 | van Tol-Geerdink, J. J., Willem Leer, J., Weijerman, P. C., van Oort, I. M., Vergunst, H., van Lin, E. N., Alfred Witjes, J., & Stalmeier, P. F. (2013). Choice between prostatectomy and radiotherapy when men are eligible for both: a randomized controlled trial of usual care vs decision aid. BJU Int, 111(4), 564-573. [https://doi.org/10.1111/j.1464-410X.2012.11402.x](https://doi.org/10.1111/j.1464-410X.2012.11402.x%20) |
| A049 | Vandemheen, K. L., O'Connor, A., Bell, S. C., Freitag, A., Bye, P., Jeanneret, A., Berthiaume, Y., Brown, N., Wilcox, P., Ryan, G., Brager, N., Rabin, H., Morrison, N., Gibson, P., Jackson, M., Paterson, N., Middleton, P., & Aaron, S. D. (2009). Randomized trial of a decision aid for patients with cystic fibrosis considering lung transplantation. Am J Respir Crit Care Med, 180(8), 761-768. [https://doi.org/10.1164/rccm.200903-0421OC](https://doi.org/10.1164/rccm.200903-0421OC%20) |
| A050 | Varelas, L., Egro, F. M., Evankovich, N., & Nguyen, V. (2020). A Randomized Controlled Trial to Assess the Use of a Virtual Decisional Aid to Improve Knowledge and Patient Satisfaction in Women Considering Breast Reconstruction Following Mastectomy [Article]. Cureus, 12(12), 8, Article e12018. [https://doi.org/10.7759/cureus.12018](https://doi.org/10.7759/cureus.12018%20) |
| A051 | Vina, E. R., Richardson, D., Medvedeva, E., Kent Kwoh, C., Collier, A., & Ibrahim, S. A. (2016). Does a Patient-centered Educational Intervention Affect African-American Access to Knee Replacement? A Randomized Trial. Clin Orthop Relat Res, 474(8), 1755-1764. [https://doi.org/10.1007/s11999-016-4834-z](https://doi.org/10.1007/s11999-016-4834-z%20) |
| A052 | Vodermaier, A., Caspari, C., Koehm, J., Kahlert, S., Ditsch, N., & Untch, M. (2009). Contextual factors in shared decision making: a randomised controlled trial in women with a strong suspicion of breast cancer. Br J Cancer, 100(4), 590-597. [https://doi.org/10.1038/sj.bjc.6604916](https://doi.org/10.1038/sj.bjc.6604916%20) |
| A053 | Vuorma, S., Teperi, J., Aalto, A. M., Hurskainen, R., Kujansuu, E., & Rissanen, P. (2004). A randomized trial among women with heavy menstruation -- impact of a decision aid on treatment outcomes and costs. Health Expect, 7(4), 327-337. [https://doi.org/10.1111/j.1369-7625.2004.00297.x](https://doi.org/10.1111/j.1369-7625.2004.00297.x%20) |
| A054 | Whelan, T., Levine, M., Willan, A., Gafni, A., Sanders, K., Mirsky, D., Chambers, S., O'Brien, M. A., Reid, S., & Dubois, S. (2004). Effect of a decision aid on knowledge and treatment decision making for breast cancer surgery: a randomized trial. Jama, 292(4), 435-441. [https://doi.org/10.1001/jama.292.4.435](https://doi.org/10.1001/jama.292.4.435%20) |
| A055 | Wilkens, S. C., Ring, D., Teunis, T., Lee, S. P., & Chen, N. C. (2019). Decision Aid for Trapeziometacarpal Arthritis: A Randomized Controlled Trial. J Hand Surg Am, 44(3), 247.e241-247.e249. [https://doi.org/10.1016/j.jhsa.2018.06.004](https://doi.org/10.1016/j.jhsa.2018.06.004%20) |
| A056 | Wilkins, E. G., Lowery, J. C., Copeland, L. A., Goldfarb, S. L., Wren, P. A., & Janz, N. K. (2006). Impact of an educational video on patient decision making in early breast cancer treatment [Journal: Article]. Medical Decision Making, 26(6), 589‐598. [https://www.cochranelibrary.com/central/doi/10.1002/central/CN-00623065/full](https://www.cochranelibrary.com/central/doi/10.1002/central/CN-00623065/full%20) |
| A057 | Wong, S. S. M., Thornton, J. G., Gbolade, B., & Bekker, H. L. (2006). A randomised controlled trial of a decision-aid leaflet to facilitate women's choice between pregnancy termination methods. BJOG: An International Journal of Obstetrics & Gynaecology, 113(6), 688-694. [https://doi.org/10.1111/j.1471-0528.2006.00930.x](https://doi.org/10.1111/j.1471-0528.2006.00930.x%20) |
| A058 | Ye, G., Qu, B., Tham, Y.-C., Zhong, Y., Jin, L., Lamoureux, E., Congdon, N., Zheng, Y., & Liu, Y. (2021). A decision aid to facilitate informed choices among cataract patients: A randomized controlled trial. Patient Education & Counseling, 104(6), 1295-1303. [https://doi.org/10.1016/j.pec.2020.10.036](https://doi.org/10.1016/j.pec.2020.10.036%20) |

# **Supplementary Table 8** Description of PDSIs in selected trials.

| **Author (Year)** | **Aim of intervention (Type of decision)** | **Intervention design** | | | | | | |  | **Survey administration** | | |
| --- | --- | --- | --- | --- | --- | --- | --- | --- | --- | --- | --- | --- |
|  |  | **Name of PDSIs** | **Co-intervention** | **Duration** | **Media format** | **PROMs data/ AI-embodiment** | **Consider value/ Use of theory** | **Communication/ Platform** |  | **Baseline** | **Posttest/ Follow-up** | **Assessment intervals** |
| Allen et al. (2016) | To compare the effectiveness  and acceptability of internet and DVD formats of PDA for osteoarthritis (Treatment) | NR | No | 45 mins | Digital | N/N | N/N | Asynchronous/ Offline computer |  | In person | Through telephone | T1: Baseline  T2: Post intervention  T3: 1 month |
| Allen et al. (2018) | To improve shared decision making and decision quality for DT LVAD (Treatment) | NR | Clinician education + pamphlets | 26 mins | Digital | N/N | Y/Y (ODSF) | Asynchronous/ Offline computer |  | In person | In person or through telephone or mail | T1: Baseline T2: Post intervention T3: 1 month T4: 6 months |
| Arterburn et al. (2011) | To improve decision quality for bariatric surgery (Treatment) | NR | Booklet | 45 mins | Digital | N/N | N/N | Asynchronous/ Offline computer |  | In person | In person | T1: Baseline T2: Post intervention T3: 3 months |
| Auvinen et al. (2004) | To improve treatment chosen by the patient for prostate cancer (Treatment) | NR | No | NR | Non-digital | N/N | N/N | Synchronous/ Booklet |  | NA | Medical record | T1: Post intervention |
| Berry et al. (2018) | To evaluate the efficacy of the web based P3P decision aid regard to decisional conflict in men with localized prostate cancer (Treatment) | P3P | Usual patient education | NR | Digital | Y/Y | Y/Y (DSF) | Asynchronous/ Online computer |  | Online | Online or through mail | T1: Baseline  T2: 1 month |
| Bozic et al. (2013) | To evaluate the impact of decision and communication aids on patient knowledge, efficiency of decision making, treatment choice, and patient and surgeon experience in patients with osteoarthritis of the hip or knee (Treatment) | NR | Booklet + question-listing telephone consultation with a trained health coach | NR | Digital | N/N | N/N | Asynchronous/ Offline computer |  | In person | In person | T1: Baseline  T2: After consultation  T3: 6 weeks |
| Coylewright et al. (2016) | To improve the knowledge and reduce decisional conflict for percutaneous coronary intervention (Treatment) | PCI Choice | No | NR | Non-digital | N/N | Y/N | Asynchronous/ Booklet |  | In person | In person | T1: Baseline T2: Post intervention |
| De Achaval et al. (2012) | To improve the functional outcomes of arthritis and reduce decisional conflict for total knee arthroplasty (Treatment) | Treatment Choices for Knee Osteoarthritis | Computer-based ACA | I1: 45 mins I2: 60 mins | Digital | I1: N/N  I2: Y/Y | Y/N | Asynchronous/ Offline computer |  | In person | In person | T1: Baseline T2: Post intervention |
| Deyo et al. (2000) | To improve outcomes and surgical choices of an interactive, diagnosis specific videodisk program for informing patients about treatment choices (Treatment) | NR | Booklet | NR | Digital | N/N | N/N | Asynchronous/ Offline computer |  | Through telephone | Through telephone or mail | T1: Baseline T2: 3 months T3: 1 year |
| Eden et al. (2014) | To evaluate if PDA help pregnant women with prior cesareans make informed decisions about having trials of labor (Treatment) | NR | No | NR | Digital | N/N | Y/Y (DCT, DMT) | Asynchronous/ Online computer |  | Online | Online | T1: Baseline  T2: Post intervention |
| Goel et al. (2001) | To improve the knowledge, and reduce anxiety, decisional conflict, or decisional regret for mastectomy (Treatment) | NR | Booklet | 26 mins | Digital | N/N | Y/N | Asynchronous/ Offline computer |  | In person | Through telephone or mail | T1: Baseline T2: Post intervention T3: 6 months |
| Gökce et al. (2019) | To improve the level of knowledge and reduce decisional conflicts for SWL vs RIRS (Treatment) | NR | No | NR | Non-digital | N/N | N/Y (OPDG) | Asynchronous/ Booklet |  | In person | In person | T1: Baseline T2: Post intervention |
| Hawley et al. (2018) | To determine the effect of iCanDecide on the rate of high-quality patient decisions—both informed and values concordant—regarding locoregional breast cancer treatment and on patient appraisal of decision making (Treatment) | iCanDecide | ACA | 46 mins | Digital | N/Y | Y/Y (HBM, SCT, FAMC) | Asynchronous/ Online computer |  | Online | Through mail or telephone | T1: Baseline  T2: 1 month T3: 9 months |
| Heller et al. (2008) | To make better informed treatment decisions for patient with breast cancer considering breast reconstruction (Treatment) | Interactive Digital Education Aid | Standard patient education | NR | Digital | N/N | N/N | Asynchronous/ Offline computer |  | In person | In person | T1: Baseline  T2: Post intervention and before surgery T3: 1 month postoperatively |
| Hutyra et al. (2019) | To identify patient preferences and guide respondents towards an evidence-based choice of treatment (Treatment) | NR | No | NR | Digital | N/Y | Y/Y (MUT) | Asynchronous/ Online computer |  | In person | In person | T1: Baseline T2: Post intervention |
| Ibrahim et al. (2017) | To improve access to TKR surgery for black patients with OA of the knee (Treatment) | NR | No | 40 mins | Digital | N/N | Y/N | Asynchronous/ Offline computer |  | In person or through telephone | T2: Medical record  T3: Medical record and through telephone | T1: Baseline  T2: 6 months  T3: 12 months |
| Jayakumar et al. (2021) | To improve decision quality, patient experience, functional outcomes, and process-level outcomes among individuals with advanced knee OA considering TKR (Treatment) | NR | No | NR | Digital | Y/Y | Y/N | Asynchronous/ Online computer |  | In person | In person | T1: Baseline T2: Post intervention T3: 4-6 months |
| Jibaja-Weiss et al. (2011) | To improve knowledge and satisfaction and reduce decisional conflict for patient with breast cancer considering surgery (Treatment) | CPtDA | Usual care | NR | Digital | N/N | Y/Y (EECT, ODSF, EDAM) | Synchronous/ Offline computer |  | In person | In person | T1: Baseline T2: Post intervention T3: 1 year |
| Kearing et al. (2016) | To improve patient knowledge and reduce decisional uncertainty about the spinal stenosis treatment choice (Treatment) | NR | Health coaching | 39 mins | Digital | N/N | Y/N | Asynchronous/ Offline computer |  | In person | In person | T1: Baseline T2: Post intervention T3: 2 weeks  T4: 6 months |
| Kennedy et al. (2002) | To evaluate the effect of PDA on treatment choices, health outcomes, and costs (Treatment) | NR | I1: Booklet  I2: Booklet + interview | 30 mins | Digital | N/N | Y/N | Asynchronous/ Offline computer |  | In person | Through telephone or mail | T1: Baseline T2: Post intervention T3: 6 months T4: 1 year T5: 2 years |
| Kleiss et al. (2021) | To test the difference in decision regret, pain self-efficacy, pain intensity, satisfaction, physical function, and treatment choice between patients who reviewed a PDA at the end of the visit and those who did not (Treatment) | PATIENT+ | No | NR | Digital | N/N | Y/Y (ODSF) | Asynchronous/ Online computer |  | Online | Through phone | T1: Baseline  T2: 4-6 weeks |
| Korteland et al. (2017) | To optimize the shared decision making in prosthetic heart valve selection (Treatment) | NR | Standard preoperative care | NR | Digital | N/Y | Y/N | Asynchronous/ Online computer |  | In person | In person | T1: Postintervention T2: 3 months postoperatively |
| Kostick et al. (2018) | To enhance the quality of decision making about LVAD therapy (Treatment) | VADDA | Standard education | NR | Non-digital | N/N | N/Y (ODSF) | Asynchronous/ Booklet |  | In person | In person or through telephone | T1: Baseline 1 T2: Post intervention + 1 day T3: 1 week T4: 1 month |
| Kuppermann et al. (2020) | To analyze the effect of a patient-centered decision support tool on rates of trial of labor and vaginal birth after cesarean delivery and decision quality (Treatment) | PROCEED | No | 10 mins | Digital | N/Y | Y/N | Synchronous/ Online computer |  | In person | Through telephone or medical record | T1: Baseline  T2: 34 – 37 weeks 6 days of gestation |
| Lam et al. (2014) | To assess the effect of decision aids on the decision-making process, satisfaction with TDM, and psychological morbidity in women undergoing breast cancer surgery (Treatment) | NR | No | NR | Non-digital | N/N | Y/N | Asynchronous/ Booklet |  | In person | In person | T1: Baseline  T2: 1-week post-consultation  T3: 1-month post-surgery  T4: 4 months post-surgery  T5: 10 months post-surgery |
| Lamers et al. (2021) | To determine whether or not PDA use influences treatment decisions in patients with low and intermediate risk prostate cancer (Treatment) | NR | Usual care | NR | Digital | N/N | Y/N | Asynchronous/ Online computer |  | In person | In person or medical record | T1: Baseline  T2: Post intervention |
| Luan et al. (2016) | To improve quality of life and reduce decisional conflict, decision regret, anxiety, and depression in the setting of breast reconstruction surgery (Treatment) | NR | No | NR | Non-digital | N/N | Y/N | Asynchronous/ Booklet |  | In person | In person | T1: Baseline T2: Postoperatively T3: 3 to 5 months postoperatively |
| Manne et al. (2016) | To improve BR knowledge and preparedness to decide and greater declines in decisional conﬂict in the setting of breast reconstruction surgery (Treatment) | BRAID | No | 74 mins | Digital | N/N | Y/Y (ODSF) | Asynchronous/ Online computer |  | In person | Through telephone or mail | T1: Baseline T2: 2 weeks |
| Manne et al. (2020) | To evaluate the feasibility and acceptability of B-Sure to facilitate informed decisions, and the impact of B-Sure in increasing CPM knowledge, reducing decisional conflict, and increasing preparedness to make the CPM decision among breast cancer patients at sporadic risk who are considering CPM (Treatment) | B-Sure | No | NR | Digital | N/N | Y/Y (ODSF) | Asynchronous/ Online computer |  | In person or online | Online | T1: Baseline  T2: 2-4 weeks after surgery |
| Metcalfe et al. (2017) | To evaluate the effectiveness of a decision aid for breast cancer prevention in women with a BRCA mutation with no previous diagnosis of cancer (Treatment) | NR | Standard care | NR | Non-digital | N/N | N/Y (ODSF) | Asynchronous/ Booklet |  | Through mail | Through telephone | T1: Baseline  T2: 3 months  T3: 6 months  T4: 12 months |
| Montgomery et al. (2007) | To reduce decisional conflict to facilitate decision on mode of delivery among pregnant women with a previous caesarian section (Treatment) | NR | Usual care | NR | Digital | N/Y | Y/N | Asynchronous/ Online computer |  | In person | In person | T1: Baseline T2: 37 weeks’ gestation |
| Parkinson et al. (2018) | To report on the cost‐effectiveness of BRECONDA (Breast RECONstruction  Decision Aid) to facilitate decisions regarding breast  reconstruction surgery, with usual care for women with breast cancer | BRECONDA | Standard information | 45 mins | Digital | N/N | Y/Y (PTM) | Asynchronous/ Online computer |  | Online | Online | T1: Baseline  T2: 1 month  T3: 6 months |
| Phelan et al. (2001) | To determine is video-based PDA with a booklet is superior to a booklet alone for informing patients about back surgery (Treatment) | Interactive videodisc | Booklet | NR | Digital | N/N | N/N | Asynchronous/ Offline computer |  | In person | In person | T1: Baseline T2: Post intervention |
| Politi et al. (2020) | To improve decision quality, enhance patient patient-clinician communication and quality of life and shorter consultation length in the setting of breast reconstruction surgery (Treatment) | BREASTChoice | No | NR | Digital | N/Y | Y/N | Asynchronous/ Online computer |  | Electronic | Electronic | T1: Post intervention (prior to/ after surgeon consultation) |
| Rivero-Santana et al. (2021) | To assess the effectiveness of a Patient Decision Aid (PtDA) for knee osteoarthritis (Treatment) | PtDA | No | NR | Digital | N/N | Y/N | Asynchronous/ Online computer |  | Online or in person | Online or through telephone | T1: Baseline  T2: Post intervention  T3: 6 months |
| Schwartz et al. (2009) | To evaluate if women using PDA is more likely to reach a definitive breast cancer risk management decision, report lower decisional conflict and increased decision satisfaction (Treatment) | Interactive CD-ROM DA | Usual care (Booklet control) | NR | Digital | N/Y | Y/Y (MVT, OFIDM) | Asynchronous/ Offline computer |  | Through telephone | Through telephone | T1: Baseline T2: 1 month T3: 6 months T4: 1 year |
| Sherman et al. (2016) | To assess the impact of an online Breast RECONstruction  Decision Aid (BRECONDA) on breast reconstruction decision-making (Treatment) | BRECONDA | Standard information | 45 mins | Digital | N/N | Y/Y (PTM) | Asynchronous/ Online computer |  | Online | Online | T1: Baseline  T2: 1 month  T3: 6 months |
| Shorten et al. (2005) | To determine whether a decision-aid for women who have experienced previous cesarean birth facilitates informed decision-making about birth options during a subsequent pregnancy (Treatment) | Birth Choices | No | NR | Non-digital | N/N | Y/Y (ODSF) | Asynchronous/ Booklet |  | In person | In person | T1: Baseline  T2: 12-18 weeks of pregnancy  T3: 28 weeks of pregnancy  T4: 36 weeks of pregnancy  T5: 6-8 weeks postnatally |
| Shue et al. (2016) | To evaluate the use of PDA for hip and knee osteoarthritis regarding the potential risks and benefits of different treatment options (Treatment) | Treatment Choices for [Hip or Knee] Osteoarthritis | Booklet-based PDSIs | NR | Digital | N/N | N/N | Asynchronous/ Offline computer |  | In person | In person | T1: Baseline  T2: 2-4 weeks |
| Stacey et al. (2016) | To improve the quality of decisions and timeliness of joint arthroplasty for those who prefer it and have osteoarthritis severe enough to require it (Treatment) | Treatment Choices for [Hip or Knee] Osteoarthritis | Booklet | 50 mins | Digital | N/N | Y/N | Asynchronous/ Offline computer |  | In person | Through telephone and mail | T1: Baseline T2: 2 weeks T3: 6 months T4: 12, 18, and 24 months |
| Stiggelbout et al. (2008) | To evaluate the impact of individualized evidence-based decision support on decision-making behavior, quality of life, and autonomy ideals of abdominal aneurysm patients (Treatment) | Individualized evidence-based brochure (IB) | No | NR | Non-digital | Y/Y | Y/N | Synchronous/ Booklet |  | NR | In person | T1: Post intervention |
| Street et al. (1995) | To improve knowledge related to breast cancer treatment, optimism about the future, involvement in decision-making process and interaction with physicians for women with breast cancer and considering surgical approach (Treatment) | Options for Treating Breast Cancer | Booklet | 30 to 45 mins | Digital | N/N | N/N | Asynchronous/ Offline computer |  | In person | In person | T1: Pre-consultation T2: Post-consultation |
| Trenaman et al. (2017) | To estimate the health and economic effects of patient decision aids for total joint replacement (Treatment) | NR | Booklet | 50 mins | Digital | N/N | Y/N | Asynchronous/ Offline computer |  | NR | Medical record | T1: Baseline T2: 2 years |
| Trenaman et al. (2020) | To evaluate the effect of patient decision aids on 1) use of joint replacement up to 7-years of follow-up, and 2) osteoarthritis-related health system costs (Treatment) | NR | Booklet | 50 mins | Digital | N/N | Y/N | Asynchronous/ Offline computer |  | NR | Medical record | T1: Baseline T2: 2 years T3: 7 years |
| Tucholka et al. (2018) | To compare patients’ knowledge after the  pre-consultation delivery of standard websites vs a web-based decision aid (Treatment) | NR | No | NR | Digital | N/N | Y/N | Asynchronous/ Online computer |  | In person | In person | T1: Baseline  T2: Post consultation |
| van Roosmalen et al. (2004a) | To evaluate the effects of a shared decision-making intervention in BRCA1/2 mutation carriers on well-being, treatment choice, and decision-related outcomes (Treatment) | NR | Brochure | 45 mins | Digital | N/N | N/N | Asynchronous/ Offline computer |  | Through mail | Through mail | T1: Baseline (T1) T2: 4 weeks after blood sampling (T2) T3: 6 weeks (2 weeks after a positive test result) (T3) |
| van Roosmalen et al. (2004b) | To evaluate the effects of a shared decision-making intervention in BRCA1/2 mutation carriers on well-being, treatment choice, and decision-related outcomes (Treatment) | Shared Decision-Making Intervention | No | NR | Digital | Y/Y | Y/N | Synchronous/ Online computer |  | In person | Through mail | T1: Baseline (T3) T2: 3 months (T4) T3: 9 months (T5) |
| van Tol-Geerdink et al. (2012) | To examine whether increased patient participation, using a decision aid, affected the treatment choice (treatment) | NR | No | NR | Non-digital | N/N | Y/N | Synchronous/ Decision board |  | In person | In person | T1: Baseline  T2: Pretreatment |
| Vandemheen et al. (2009) | To evaluate whether use of the decision aid increased knowledge about the options, improved realistic expectations, and decreased decisional conflict in adult patients with cystic fibrosis (Treatment) | NR | No | NR | Digital | N/N | N/Y (ODSF) | Asynchronous/ Online computer |  | In person | Through telephone | T1: Baseline  T2: 3 weeks T3: 12 months |
| Varelas et al. (2020) | To assess the impact of a virtual breast reconstruction decision aid program on the decision-making process of breast cancer patients considering breast reconstruction (Treatment) | Emmi Decide | Traditional consultation | 20 mins | Digital | N/N | Y/N | Asynchronous/ Online computer |  | In person | In person | T1: Baseline  T2: Post consultation |
| Vina et al. (2016) | To evaluate if a patient-centered intervention consisting of a PDA for knee OA and motivational interviewing improve the proportion of referrals of blacks with knee OA to orthopedic surgery and increase patients’ willingness to undergo TKA (Treatment) | NR | Motivational interviewing with certified interventionist | 40 mins | Digital | N/N | N/N | Asynchronous/ Offline computer |  | In person | Through telephone | T1: Baseline  T2: 2 weeks  T3: 3 months  T4: 12 months |
| Vodermaier et al. (2009) | To examine the impact of a decision aid intervention in breast cancer inpatients on decisional conflict, uptake rates of treatment options, length of consultation, patient involvement in care and satisfaction (Treatment) | NR | Information brochure | 20 mins | Non-digital | N/N | N/N | Synchronous/ Board |  | In person | Through mail | T1: Baseline  T2: 1 week |
| Vuorma et al. (2004) | To evaluate the effects of a decision aid for menorrhagia on treatment outcomes and costs (Treatment) | NR | No | NR | Non-digital | N/N | N/N | Asynchronous/ Booklet |  | Through mail | In person, through mail or medical records | T1: Baseline  T2: 3 months T3: 12 months |
| Whelan et al. (2004) | To improve communication and empower patients in the decision-making process for those considering breast surgery (Treatment) | NR | No | 21 mins | Non-digital | N/N | N/N | Synchronous/ Board |  | In person | Through telephone | T1: Baseline T2: Post consultation T3: 6 months T4: 12 months |
| Wilkens et al. (2019) | To reduce decisional conflict, depression and disabilities and improve satisfaction for those with TMS arthritis seeking surgical treatment (Treatment) | PATIENT+ | Brochure | 15-20 mins | Digital | N/N | Y/Y (ODSF) | Asynchronous/ Online computer |  | Electronic | Electronic or through telephone | T1: Baseline T2: 6 weeks T3: 6 months |
| Wilkins et al. (2006) | To evaluate the impact of an early breast cancer treatment educational video on patients’ decisional preferences and behavior (Treatment) | Early-Stage Breast Cancer: Choosing  Your Surgery | Written materials | 60 mins | Digital | N/N | N/N | Asynchronous/ Offline computer |  | In person | Through telephone | T1: Baseline  T2: Post-intevention T3: 1 week |
| Wong et al. (2006) | To evaluate the effectiveness of a decision aid to help women choose between surgical and medical methods of pregnancy termination (Treatment) | NR | No | NR | Non-digital | N/N | Y/N | Asynchronous/ Offline computer |  | In person | In person | T1: Post consultation  T2: Post procedure |
| Ye et al. (2021) | To assess the effect of a patient decision aid on the quality of decision-making for patients with age-related cataracts (Treatment) | NR | No | NR | Non-digital | N/N | Y/N | Asynchronous/ Booklet |  | In person | Through telephone | T1: Baseline T2: 2 weeks |
| ACA: Adaptive Conjoint Analysis; AI: Artificial Intelligence; BR: Breast Reconstruction; CPM: Contralateral Prophylactic Mastectomy; PDSIs: Patient decision support interventions; DCT: Decision conflict theory; DMT: Decision-making theory; DSF: O’Connor’s Decision Support Framework; EDAM: Edutainment Decision Aid Model; EECT: Entertainment Education Concepts and Theory; FAMC: Framework for the study of access to medical care; HBM: Health Belief Model; I: Intervention; LVAD: Left Ventricular Assist Device; MUT: Microeconomic Utility Theory; MVT: Multiattribute Value Theory; N: No; NA: Not Applicable NR: Not recorded; OA: Osteoarthritis; ODSF: Ottawa Decision Support Framework; OFIDM: Ottawa Framework for Informed Decision Making; OPDG: Ottawa Personal Decision Guide; PROMS: Patient-reported outcome measures; PTM: Psychological theoretical models; RIRS: Retrograde Intrarenal Surgery; SCT: Social Cognitive Theory; SWL: Shock Wave Lithotripsy; TKR: Total Knee Replacement; TMC: Trapeziometacarpal; Y: Yes | | | | | | | | | | | | |

# **Supplementary Table 9** The International Patient Decision Aid Standards Instrument “Short Form” (IPDASi-SF) Ratings.

| **Study code** | 1 | 2 | 3 | 4 | 5 | 6 | 7 | 8 | 9 | 10 | 11 | 12 | 13 | 14 | 15 | 16 | 17 | 18 | 19 | 20 | 21 | 22 | 23 | 24 | 25 | 26 | 27 | 28 | 29 |
| --- | --- | --- | --- | --- | --- | --- | --- | --- | --- | --- | --- | --- | --- | --- | --- | --- | --- | --- | --- | --- | --- | --- | --- | --- | --- | --- | --- | --- | --- |
| **Information** |  |  |  |  |  |  |  |  |  |  |  |  |  |  |  |  |  |  |  |  |  |  |  |  |  |  |  |  |  |
| Options available | + | + | + | + | + | + | + | + | + | + | + | + | + | + | + | + | + | + | + | + | + | + | + | + | + | + | + | + | + |
| Positive features | + | + | + | + | + | + | + | + | + | + | + | + | + | + | + | + | + | + | + | + | + | + | + | + | + | + | + | + | + |
| Negative features | + | + | + | + | + | + | + | + | + | + | + | + | + | + | + | + | + | + | + | + | + | + | + | + | + | + | + | + | + |
| Fair comparison | + | + | + | + | + | + | + | + | + | + | + | + | + | + | + | + | + | + | + | + | + | + | + | + | + | + | + | + | + |
| **Probabilities** |  |  |  |  |  |  |  |  |  |  |  |  |  |  |  |  |  |  |  |  |  |  |  |  |  |  |  |  |  |
| Reference class |  | + | + | + | + | + | + | + | + | + | + | + | + | + | + | + | + | + | + |  | + | + | + | + | + | + | + | + | + |
| Event rates |  | + |  | + | + |  | + | + | + | + | + | + | + |  |  | + | + | + | + |  | + | + | + | + | + | + | + | + | + |
| Compare probabilities |  | + | + | + | + | + | + | + | + | + | + | + | + | + | + | + | + | + | + |  | + | + | + | + | + | + | + | + | + |
| **Values** |  |  |  |  |  |  |  |  |  |  |  |  |  |  |  |  |  |  |  |  |  |  |  |  |  |  |  |  |  |
| Personal importance |  | + |  | + | + |  | + | + |  | + | + |  | + |  | + | + | + | + | + | + | + | + |  | + | + | + | + | + | + |
| **Development** |  |  |  |  |  |  |  |  |  |  |  |  |  |  |  |  |  |  |  |  |  |  |  |  |  |  |  |  |  |
| Patients' needs |  | + | + |  | + |  | + |  |  | + | + | + | + | + |  |  | + | + |  | + |  | + | + | + |  | + | + | + | + |
| Impartial review |  | + | + |  |  |  | + |  |  | + | + | + | + | + |  |  | + | + |  | + |  | + | + | + |  | + | + | + | + |
| Tested with patients |  | + | + |  | + |  | + |  |  | + | + | + | + |  |  |  | + | + |  | + |  | + | + | + |  | + |  | + | + |
| **Disclosure** |  |  |  |  |  |  |  |  |  |  |  |  |  |  |  |  |  |  |  |  |  |  |  |  |  |  |  |  |  |
| Funding information |  | + | + | + | + | + | + | + |  | + |  |  | + |  | + | + | + | + | + | + |  | + | + | + | + | + |  | + | + |
| **DA evaluation** |  |  |  |  |  |  |  |  |  |  |  |  |  |  |  |  |  |  |  |  |  |  |  |  |  |  |  |  |  |
| Knowledge | + | + | + |  |  | + | + |  | + |  | + | + | + | + | + |  | + | + | + | + |  | + | + | + | + | + |  | + | + |
| Decision quality | + | + | + |  | + |  | + | + |  | + | + | + | + |  | + |  | + | + |  |  |  | + | + | + |  | + | + | + | + |
| **Evidence** |  |  |  |  |  |  |  |  |  |  |  |  |  |  |  |  |  |  |  |  |  |  |  |  |  |  |  |  |  |
| Citations to studies |  | + | + |  | + | + | + | + |  | + | + | + | + |  |  |  | + | + | + | + | + | + | + | + |  | + |  |  |  |
| **Production date** |  | + |  | + | + |  | + |  |  |  | + | + | + |  | + |  |  | + |  |  | + | + | + | + |  | + |  |  |  |
| **Total '+'** | 6 | 16 | 13 | 10 | 14 | 9 | 16 | 11 | 8 | 14 | 15 | 14 | 16 | 9 | 11 | 9 | 15 | 16 | 11 | 11 | 10 | 16 | 15 | 16 | 10 | 16 | 11 | 14 | 14 |

| **Study code** | 30 | 31 | 32 | 33 | 34 | 35 | 36 | 37 | 38 | 39 | 40 | 41 | 42 | 43 | 44 | 45 | 46 | 47 | 48 | 49 | 50 | 51 | 52 | 53 | 54 | 55 | 56 | 57 | 58 | Total | % |
| --- | --- | --- | --- | --- | --- | --- | --- | --- | --- | --- | --- | --- | --- | --- | --- | --- | --- | --- | --- | --- | --- | --- | --- | --- | --- | --- | --- | --- | --- | --- | --- |
| **Information** |  |  |  |  |  |  |  |  |  |  |  |  |  |  |  |  |  |  |  |  |  |  |  |  |  |  |  |  |  |  |  |
| Options available | + | + | + | + | + | + | + | + | + | + | + | + | + | + | + | + | + | + | + | + | + | + | + | + | + | + | + | + | + | 58 | 100.0 |
| Positive features | + | + | + | + | + | + | + | + | + | + | + | + | + | + | + | + | + | + | + | + | + | + | + | + | + | + | + | + | + | 58 | 100.0 |
| Negative features | + | + | + | + | + | + | + | + | + | + | + | + | + | + | + | + | + | + | + | + | + | + | + | + | + | + | + | + | + | 58 | 100.0 |
| Fair comparison | + | + | + | + | + | + | + | + | + | + | + | + | + | + | + | + | + | + | + | + | + | + | + | + | + | + | + | + | + | 58 | 100.0 |
| **Probabilities** |  |  |  |  |  |  |  |  |  |  |  |  |  |  |  |  |  |  |  |  |  |  |  |  |  |  |  |  |  |  |  |
| Reference class | + | + | + | + | + | + | + | + | + |  | + | + |  | + | + |  | + | + | + | + |  | + | + |  | + | + | + | + | + | 51 | 87.9 |
| Event rates | + | + | + | + | + | + | + | + | + |  | + | + |  | + | + |  | + | + | + | + |  | + | + |  | + | + | + | + | + | 47 | 81.0 |
| Compare probabilities | + | + | + | + | + | + | + | + | + |  | + | + |  | + | + |  | + | + | + | + |  | + | + |  | + | + | + | + | + | 51 | 87.9 |
| **Values** |  |  |  |  |  |  |  |  |  |  |  |  |  |  |  |  |  |  |  |  |  |  |  |  |  |  |  |  |  |  |  |
| Personal importance | + | + | + | + | + | + | + | + | + |  | + | + | + | + | + | + |  | + | + |  | + | + |  |  |  | + |  | + | + | 44 | 75.9 |
| **Development** |  |  |  |  |  |  |  |  |  |  |  |  |  |  |  |  |  |  |  |  |  |  |  |  |  |  |  |  |  |  |  |
| Patients' needs | + | + | + | + | + | + | + | + | + |  |  |  |  |  |  |  | + | + |  |  |  |  | + |  | + |  | + | + | + | 35 | 60.3 |
| Impartial review | + | + | + | + | + | + | + | + | + |  |  |  |  |  |  |  | + |  |  |  |  |  | + |  | + |  | + | + | + | 33 | 56.9 |
| Tested with patients |  | + | + | + | + | + | + | + | + |  |  |  |  |  |  |  |  |  |  |  |  |  | + |  | + |  |  | + | + | 29 | 50.0 |
| **Disclosure** |  |  |  |  |  |  |  |  |  |  |  |  |  |  |  |  |  |  |  |  |  |  |  |  |  |  |  |  |  |  |  |
| Funding information |  | + | + | + | + | + | + | + | + | + | + | + | + | + | + | + | + | + | + | + |  | + | + | + | + |  | + |  | + | 47 | 81.0 |
| **DA evaluation** |  |  |  |  |  |  |  |  |  |  |  |  |  |  |  |  |  |  |  |  |  |  |  |  |  |  |  |  |  |  |  |
| Knowledge | + | + |  | + | + | + | + |  | + | + | + |  | + | + | + | + | + |  |  | + | + |  |  |  |  |  | + | + | + | 41 | 70.7 |
| Decision quality | + | + | + |  | + | + | + | + | + | + | + |  |  | + | + |  | + | + |  | + | + |  | + |  | + | + |  | + | + | 41 | 70.7 |
| **Evidence** |  |  |  |  |  |  |  |  |  |  |  |  |  |  |  |  |  |  |  |  |  |  |  |  |  |  |  |  |  |  |  |
| Citations to studies |  | + | + | + | + |  | + | + | + |  |  |  |  |  |  |  | + | + | + |  |  |  |  |  |  |  | + | + | + | 32 | 55.2 |
| **Production date** |  | + | + |  | + |  | + | + |  |  |  |  |  |  |  |  |  |  |  |  |  |  | + |  | + | + |  | + | + | 24 | 41.4 |
| **Total '+'** | 12 | 16 | 15 | 14 | 16 | 14 | 16 | 15 | 15 | 7 | 11 | 9 | 7 | 11 | 11 | 7 | 13 | 12 | 10 | 10 | 7 | 9 | 13 | 5 | 13 | 10 | 12 | 15 | 16 |  |  |

# **Supplementary Table 10** GRADE summary of evidence for PDSIs on decision-making related, patient-reported outcomes and healthcare resources outcomes.

(a) Decision-making related outcome

| **Certainty assessment** | | | | | | | **№ of patients** | | **Effect** | | **Certainty** | **Importance** |
| --- | --- | --- | --- | --- | --- | --- | --- | --- | --- | --- | --- | --- |
| **№ of studies** | **Study design** | **Risk of bias** | **Inconsistency** | **Indirectness** | **Imprecision** | **Other considerations** | **PDSIs** | **Comparator** | **Relative (95% CI)** | **Absolute (95% CI)** |
| **Invasive treatment choice (Post-intervention)** | | | | | | | | | | | | |
| 42 | randomised trials | not serious | serious^a^ | serious^b^ | serious^c^ | none | 2396/5136 (46.7%) | 2302/4802 (47.9%) | **RR 0.97** (0.90 to 1.04) | **14 fewer per 1,000** (from 48 fewer to 19 more) | ⨁◯◯◯ Very low | IMPORTANT |
| **Invasive treatment choice (follow-up: median 9 months)** | | | | | | | | | | | | |
| 6 | randomised trials | not serious | very serious^d^ | serious^b^ | not serious | none | 371/649 (57.2%) | 409/780 (52.4%) | **RR 1.05** (0.85 to 1.30) | **26 more per 1,000** (from 79 fewer to 157 more) | ⨁◯◯◯ Very low | IMPORTANT |
| **Decisional conflict (Post-intervention)** | | | | | | | | | | | | |
| 27 | randomised trials | not serious | very serious^d^ | serious^b^ | serious^e^ | publication bias strongly suspected^f^ | 2942 | 2784 | - | SMD **0.29 SD lower** (0.41 lower to 0.16 higher) | ⨁◯◯◯ Very low | IMPORTANT |
| **Decisional conflict (follow-up: median 6 months)** | | | | | | | | | | | | |
| 6 | randomised trials | not serious | not serious | serious^b^ | serious^e^ | none | 538 | 562 | - | SMD **0.11 SD lower** (0.23 lower to 0.11 higher) | ⨁⨁◯◯ Low | IMPORTANT |
| **Subscale of decisional conflict (Informed subscale)** | | | | | | | | | | | | |
| 13 | randomised trials | not serious | very serious^d^ | serious^b^ | serious^e^ | none^f^ | 1701 | 1514 | - | SMD **0.38 SD lower** (0.61 lower to 0.14 lower) | ⨁◯◯◯ Very low | IMPORTANT |
| **Subscale of decisional conflict (Values clarity subscale)** | | | | | | | | | | | | |
| 12 | randomised trials | not serious | very serious^d^ | serious^b^ | serious^e^ | none | 1547 | 1426 | - | SMD **0.25 SD lower** (0.41 lower to 0.08 lower) | ⨁◯◯◯ Very low | IMPORTANT |
| **Subscale of decisional conflict (Support subscale)** | | | | | | | | | | | | |
| 12 | randomised trials | not serious | serious^a^ | serious^b^ | serious^e^ | none | 1549 | 1354 | - | SMD **0.17 SD lower** (0.29 lower to 0.04 lower) | ⨁◯◯◯ Very low | IMPORTANT |
| **Subscale of decisional conflict (Uncertainty subscale)** | | | | | | | | | | | | |
| 13 | randomised trials | not serious | not serious | serious^b^ | serious^e^ | none | 1703 | 1515 | - | SMD **0.1 SD lower** (0.17 lower to 0.02 lower) | ⨁⨁◯◯ Low | IMPORTANT |
| **Subscale of decisional conflict (Effective decision subscale)** | | | | | | | | | | | | |
| 13 | randomised trials | not serious | not serious | serious^b^ | serious^e^ | publication bias strongly suspected^f^ | 1702 | 1510 | - | SMD **0.14 SD lower** (0.23 lower to 0.04 lower) | ⨁◯◯◯ Very low | IMPORTANT |
| **Satisfaction with decision-making (Post-intervention)** | | | | | | | | | | | | |
| 18 | randomised trials | not serious | serious^a^ | serious^b^ | serious^e^ | none | 1927 | 1817 | - | SMD **0.09 SD higher** (0.05 lower to 0.22 higher) | ⨁◯◯◯ Very low | IMPORTANT |
| **Satisfaction with decision-making (follow-up: median 6 months)** | | | | | | | | | | | | |
| 4 | randomised trials | not serious | not serious | not serious | serious^e^ | none | 323 | 326 | - | SMD **0.16 SD higher** (0.01 higher to 0.32 higher) | ⨁⨁⨁◯ Moderate | IMPORTANT |
| **Disease and Treatment Knowledge (Post-intervention)** | | | | | | | | | | | | |
| 15 | randomised trials | not serious | very serious^d^ | serious^b^ | serious^e^ | none | 2036 | 2082 | - | SMD **0.32 SD higher** (0.15 higher to 0.49 higher) | ⨁◯◯◯ Very low | IMPORTANT |
| **Disease and Treatment Knowledge (follow-up: median 4.5 months)** | | | | | | | | | | | | |
| 4 | randomised trials | not serious | not serious | not serious | serious^e^ | none | 296 | 290 | - | SMD **0.1 SD higher** (0.06 lower to 0.27 higher) | ⨁⨁⨁◯ Moderate | IMPORTANT |
| **Decisional regret (Post-intervention)** | | | | | | | | | | | | |
| 8 | randomised trials | not serious | very serious^d^ | serious^b^ | serious^e^ | none | 512 | 531 | - | SMD **0.2 SD lower** (0.53 lower to 0.13 higher) | ⨁◯◯◯ Very low | IMPORTANT |
| **Decisional regret (follow-up: median 6 months)** | | | | | | | | | | | | |
| 2 | randomised trials | not serious | very serious^d^ | not serious | not serious | none | 262 | 289 | - | SMD **0.03 SD higher** (0.36 lower to 0.42 higher) | ⨁⨁◯◯ Low | IMPORTANT |
| **Preparedness for decision-making** | | | | | | | | | | | | |
| 5 | randomised trials | not serious | not serious | serious^b^ | serious^e^ | none | 474 | 479 | - | SMD **0.22 SD higher** (0.09 higher to 0.34 higher) | ⨁⨁◯◯ Low | IMPORTANT |
| **Decision quality (Post-intervention)** | | | | | | | | | | | | |
| 3 | randomised trials | not serious | very serious^d^ | not serious | serious^e^ | none | 380 | 365 | - | SMD **0.53 SD higher** (0.02 lower to 1.09 higher) | ⨁◯◯◯ Very low | IMPORTANT |
| **Decision quality - Values-concordance** | | | | | | | | | | | | |
| 5 | randomised trials | not serious | very serious^d^ | serious^b^ | not serious | none | 323/862 (37.5%) | 178/850 (20.9%) | **RR 1.98** (1.15 to 3.39) | **240 more per 1,000** (from 15 more to 596 more) | ⨁◯◯◯ Very low | IMPORTANT |
| **Shared decision-making (Post-intervention)** | | | | | | | | | | | | |
| 3 | randomised trials | not serious | very serious^d^ | not serious | serious^e^ | none | 761 | 766 | - | SMD **0.22 SD higher** (0.31 lower to 0.75 higher) | ⨁◯◯◯ Very low | IMPORTANT |
| **Decision self-efficacy** | | | | | | | | | | | | |
| 3 | randomised trials | not serious | not serious | not serious | serious^e^ | none | 797 | 798 | - | SMD **0.02 SD higher** (0.07 lower to 0.12 higher) | ⨁⨁⨁◯ Moderate | IMPORTANT |
| **Outcome expectations** | | | | | | | | | | | | |
| 3 | randomised trials | not serious | very serious^d^ | not serious | serious^e^ | none | 258 | 268 | - | SMD **0.11 SD higher** (0.57 lower to 0.8 higher) | ⨁◯◯◯ Very low | IMPORTANT |
| **CI:** confidence interval; **RR:** risk ratio; **SMD:** standardised mean difference  **Explanations**  a.Heterogeneity is high (I2 > 40%).  b.There are variation of delivery platforms for decision aids among different elective surgical patients.  c.Trial used less than sample size of 50 per group, with wide confidence intervals.  d.Heterogeneity is high (I2 > 75%).  e.Majority of the trial (>50%) used less than sample size of 50-100 per group, with wide confidence intervals.  f.The funnel plot showed the scatter plots of the included trials were asymmetrically centred on the summary effect estimate of the analysis. In addition, the p-value for Egger's regression <0.05. | | | | | | | | | | | | |

(b) Patient-reported outcomes

| **Certainty assessment** | | | | | | | **№ of patients** | | **Effect** | | **Certainty** | **Importance** |
| --- | --- | --- | --- | --- | --- | --- | --- | --- | --- | --- | --- | --- |
| **№ of studies** | **Study design** | **Risk of bias** | **Inconsistency** | **Indirectness** | **Imprecision** | **Other considerations** | **PDSIs** | **Comparator** | **Relative (95% CI)** | **Absolute (95% CI)** |
| **General HRQoL (Post-intervention)** | | | | | | | | | | | | |
| 5 | randomised trials | not serious | serious^a^ | serious^b^ | serious^c^ | none | 414 | 448 | - | SMD **0.02 SD higher** (0.2 lower to 0.25 higher) | ⨁◯◯◯ Very low | IMPORTANT |
| **General HRQoL (follow-up: median 6 months)** | | | | | | | | | | | | |
| 3 | randomised trials | not serious | serious^a^ | not serious | serious^c^ | none | 215 | 246 | - | SMD **0.13 SD higher** (0.18 lower to 0.44 higher) | ⨁⨁◯◯ Low | IMPORTANT |
| **Physical health (Post-intervention)** | | | | | | | | | | | | |
| 2 | randomised trials | not serious | not serious | not serious | serious^c^ | none | 143 | 142 | - | SMD **0.15 SD higher** (0.08 lower to 0.38 higher) | ⨁⨁⨁◯ Moderate | IMPORTANT |
| **Mental health (Post-intervention)** | | | | | | | | | | | | |
| 2 | randomised trials | not serious | very serious^d^ | not serious | not serious | none | 180 | 183 | - | SMD **0.14 SD higher** (0.34 lower to 0.62 higher) | ⨁⨁◯◯ Low | IMPORTANT |
| **Mental Health (follow-up: postoperative)** | | | | | | | | | | | | |
| 2 | randomised trials | not serious | not serious | not serious | not serious | none | 180 | 183 | - | SMD **0.07 SD higher** (0.13 lower to 0.28 higher) | ⨁⨁⨁⨁ High | IMPORTANT |
| **Condition-specific HRQoL** | | | | | | | | | | | | |
| 5 | randomised trials | not serious | serious^a^ | serious^b^ | very serious^e^ | none | 193 | 186 | - | SMD **0.28 SD higher** (0.11 lower to 0.67 higher) | ⨁◯◯◯ Very low | IMPORTANT |
| **Anxiety (Post-intervention)** | | | | | | | | | | | | |
| 9 | randomised trials | not serious | serious^a^ | serious^b^ | serious^c^ | none | 864 | 776 | - | SMD **0.03 SD lower** (0.2 lower to 0.13 higher) | ⨁◯◯◯ Very low | IMPORTANT |
| **Anxiety (follow-up: median 6 months)** | | | | | | | | | | | | |
| 3 | randomised trials | not serious | not serious | not serious | serious^c^ | none | 251 | 262 | - | SMD **0.05 SD lower** (0.25 lower to 0.16 higher) | ⨁⨁⨁◯ Moderate | IMPORTANT |
| **Depression (Post-intervention)** | | | | | | | | | | | | |
| 6 | randomised trials | not serious | not serious | serious^b^ | not serious | none | 576 | 615 | - | SMD **0.04 SD higher** (0.1 lower to 0.17 higher) | ⨁⨁⨁◯ Moderate | IMPORTANT |
| **Depression (follow-up: median 6 months)** | | | | | | | | | | | | |
| 4 | randomised trials | not serious | not serious | not serious | not serious | none | 355 | 394 | - | SMD **0.02 SD lower** (0.19 lower to 0.14 higher) | ⨁⨁⨁⨁ High | IMPORTANT |
| **Perceived Stress (Post-intervention)** | | | | | | | | | | | | |
| 3 | randomised trials | not serious | not serious | not serious | not serious | none | 349 | 380 | - | SMD **0 SD**  (0.19 lower to 0.19 higher) | ⨁⨁⨁⨁ High | IMPORTANT |
| **CI:** confidence interval; **SMD:** standardised mean difference  **Explanations**  a.Heterogeneity is high (I2 > 40%).  b.There are variation of delivery platforms for decision aids among different elective surgical patients.  c.Majority of the trial (>50%) used less than sample size of 50-100 per group, with wide confidence intervals.  d. Heterogeneity is high (I2 > 75%).  e.Majority of the trial (>50%) used less than sample size of 50 per group, with wide confidence intervals. | | | | | | | | | | | | |

(c) Healthcare resources outcome

| **Certainty assessment** | | | | | | | **№ of patients** | | **Effect** | | **Certainty** | **Importance** |
| --- | --- | --- | --- | --- | --- | --- | --- | --- | --- | --- | --- | --- |
| **№ of studies** | **Study design** | **Risk of bias** | **Inconsistency** | **Indirectness** | **Imprecision** | **Other considerations** | **PDSIs** | **Comparator** | **Relative (95% CI)** | **Absolute (95% CI)** |
| **Consultation time (assessed with: minutes)** | | | | | | | | | | | | |
| 4 | randomised trials | not serious | not serious | not serious | serious^a^ | none | 187 | 175 | - | MD **0.04 mins higher** (0.17 lower to 0.24 higher) | ⨁⨁⨁◯ Moderate | IMPORTANT |
| **CI:** confidence interval; **MD:** mean difference  **Explanations**  a.Majority of the trial (>50%) used less than sample size of 50-100 per group, with wide confidence intervals. | | | | | | | | | | | | |

# **Supplementary Figure 1.** Risk of bias summary.

(a)
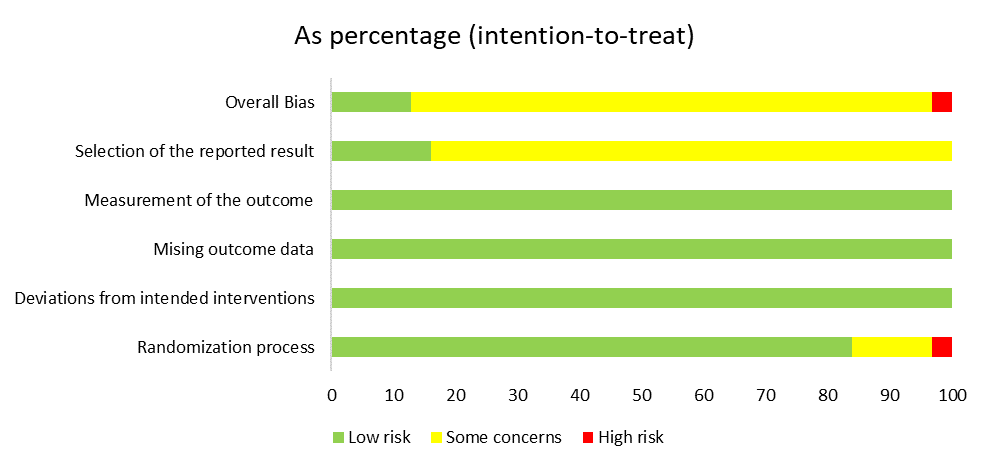


(b)
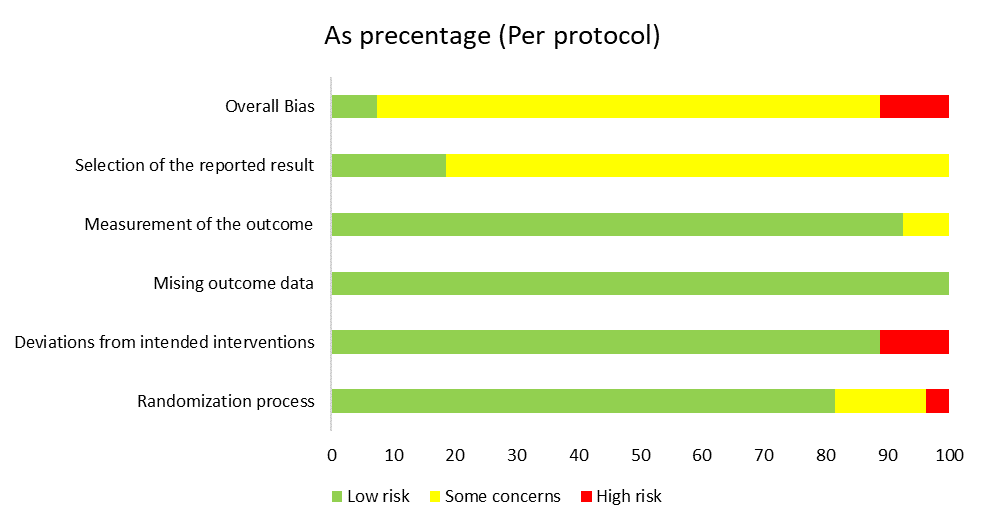


# **Supplementary Figure 2.** Risk of bias graph stratified by (a) intention-to-treat and (b) per-protocol analysis.

# **Supplementary Figure 3.** Forest plot of risk ratio (95% CI) for PDSIs and comparator groups on actual invasive treatment choice

Post-intervention

Follow-up [1 – 85 mths]

# **Supplementary Figure 4.** Forest plot of Hedges’s *g* (95% CI) for PDSIs and comparator groups on decision-making related outcomes

Decisional conflict

Post-intervention

Follow-up [1 – 6 mths]

Subscale of decisional conflict scale

Informed subscale

Values clarity subscale

Support subscale

Uncertainty subscale

Effective decision subscale

Satisfaction with decision-making

Post-intervention

Follow-up [6 mths]

Disease and treatment knowledge

Post-intervention

Follow-up [1 – 6 mths]

Decisional regret

Post-surgery [immediately – 6 mths]

Follow-up [4 – 6 mths]

Preparedness for decision-making

Decision quality

Post-intervention

Values-concordance

Shared decision-making

Decision self-efficacy

Outcome expectations

# **Supplementary Figure 5.** Forest plot of Hedges’s *g* (95% CI) for PDSIs and comparator groups on patient-reported outcomes

General HRQoL

Post-intervention

Follow-up [3 – 9 mths]

Physical Health

Mental Health

Post-intervention

Follow-up [post-op – 4 mths]

Condition-specific HRQoL

Anxiety

Post-intervention

Follow-up [4 – 9 mths]

Depression

Post-intervention

Follow-up [4 – 9 mths]

Perceived Stress

# **Supplementary Figure 6.** Forest plot of Hedges’s *g* (95% CI) for PDSIs and comparator groups on healthcare resources use outcomes

Consultation time (minutes)

# **Supplementary Figure 7.** Forest plot of risk ratio (95% CI) for PDSIs and comparator groups on actual invasive treatment choice, stratified by different modifiers

Types of elective surgery

PROM-based

Mode of delivery

Consider value

Use of theoretical framework

# **Supplementary Figure 8.** Forest plot of Hedges’s *g* (95% CI) for PDSIs and comparator groups on decisional conflict, stratified by different modifiers

Types of elective surgery

PROM-based

Mode of delivery

Consider value

Use of theoretical framework

# **Supplementary Figure 9.** Forest plot of Hedges’s *g* (95% CI) for PDSIs and comparator groups on patient satisfaction, stratified by different modifiers

Types of elective surgery

PROM-based

Mode of delivery

Consider value

Use of theoretical framework

# **Supplementary Figure 10.** Forest plot of Hedges’s *g* (95% CI) for PDSIs and comparator groups on disease and treatment knowledge, stratified by different modifiers

Types of elective surgery

Mode of delivery

Consider value

Use of theoretical framework

(a) (b)

(c) (d)

# **Supplementary Figure 11.** Egger's regression test, Begg’s test and funnel plots of precision by Hedges’s g of (a) invasive treatment choice (N = 42), (b) decisional conflict (N = 27), (c) satisfaction with decision-making (N = 18), and (d) disease and treatment knowledge outcome (N = 15).

Observed: Hedges’ *g* -0.287 (-0.414, -0.159)

Observed + Imputed: Hedges’ *g* -0.214 (-0.354, -0.073)

# **Supplementary Figure 12.** Nonparametric trim-and-fill analysis of publication bias for decisional conflict outcome.


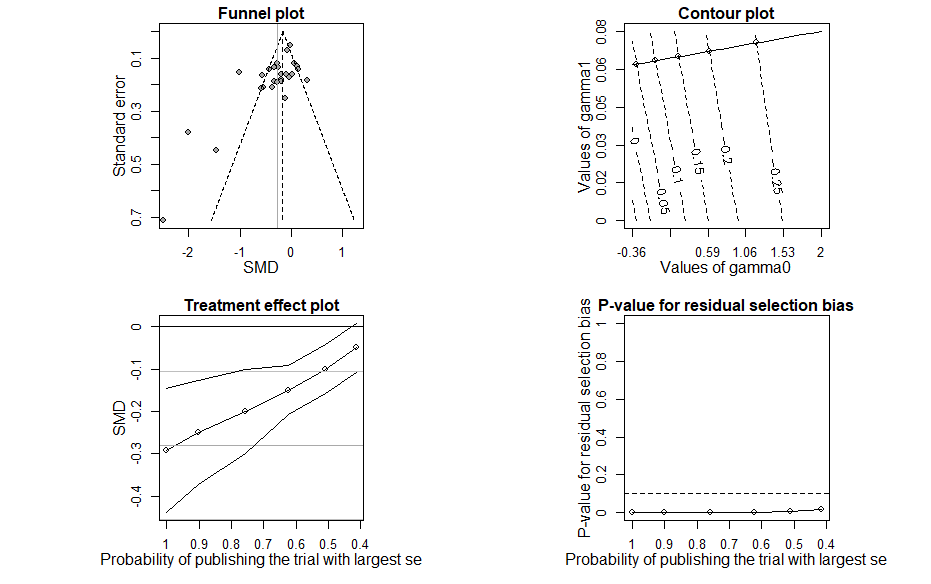


# **Supplementary Figure 13.** Funnel, contour, treatment effect and *p*-value for residual selection bias plot.
